# Supplementary material for: Molecular basis for multidrug efflux by an anaerobic-associated RND transporter
Source: Nat Commun. 2025 Dec 3;16:10601. doi: 10.1038/s41467-025-65565-7 (PMC12675537; doi:10.1038/s41467-025-65565-7)
Supplement: Supplementary file 1 — Supplementary Information [file 41467_2025_65565_MOESM1_ESM.pdf]

Molecular basis for multidrug efflux by an anaerobic-associated RND transporter

Supplementary Information

Ryan Lawrence, Mohd Athar, Muhammad R. Uddin, Christopher Adams, Joana S. Sousa, Oliver Durrant, Sophie Lellman, Lucy Sutton, C. William Keevil, Nisha Patel, Christine E. Prosser, David McMillan, Helen I. Zgurskaya, Attilio V. Vargiu, Zainab Ahdash, Eamonn Reading

Table of Contents

TABLE OF CONTENTS 2

SECTION 1 SUPPLEMENTARY DISCUSSION 4

Substrate docking at channels 1, 2 and 3 in MdtF compared to AcrB 4

SECTION 2 SUPPLEMENTARY TABLES 5

Supplementary Table 1 | Cryo-EM data collection, refinement, and validation statistics 5

Supplementary Table 2 | LC-MS lipid analysis of extracted MdtF SMALP. 6

Supplementary Table 3 | Molecular docking 7

Supplementary Table 4 | MIC calculations of CH3 mutants 8

Supplementary Table 5 | Primer table 9

Supplementary Table 6 | Structural and compositional details of MdtF^{WT}, MdtF^{V610F}, and AcrB systems 10

Supplementary Table 7 | Global RMSD (in Å) comparisons of MdtF access, binding, and extrusion states (Cα-atoms) to published RND structures 11

Supplementary Table 8 | MD simulations checklist 12

SECTION 3 SUPPLEMENTARY FIGURES 15

Supplementary Fig. 1 | Expression, purification, and characterisation of MdtF in SMALPs 15

Supplementary Fig. 2 | MdtF^{WT} cryo-EM data processing 16

Supplementary Fig. 3 | Quality of MdtF^{WT} cryo-EM map 17

Supplementary Fig. 4 | MdtF^{V610F} cryo-EM data processing 18

Supplementary Fig. 5 | Quality of MdtF^{V610F} cryo-EM map 19

Supplementary Fig. 6 | MdtF^{V610F}-R6G cryo-EM data processing 20

Supplementary Fig. 7 | Quality of MdtF^{V610F}-R6G cryo-EM map 21

Supplementary Fig. 8 | Lipidomic analysis of MdtF-SMALPs lipid belt 22

Supplementary Fig. 9 | Multiple sequence alignment of MdtF and AcrB 24

Supplementary Fig. 10 | Porter domain conformation comparison between MdtF^{WT} and AcrB 26

Supplementary Fig. 11 | Assessment of AlphaFold Prediction of MdtF Structures 27

Supplementary Fig. 12 | MdtF^{WT} demonstrates stationary transmembrane domain compared to AcrB-SMALPs 28

Supplementary Fig. 13 | Structural transitions observed in TM2 and TM8 29

Supplementary Fig. 14 | MdtF^{WT} channels of export and channel 3 entrance 30

Supplementary Fig. 15 | Docking poses in the DBP of MdtF 31

Supplementary Fig. 16 | Docking poses at the CH1 entrance of MdtF^{WT} and MdtF^{V610F} 32

Supplementary Fig. 17 | Docking poses at the CH2 entrance of MdtF^{WT} and MdtF^{V610F} 33

Supplementary Fig. 18 | Docking poses at the CH3 entrance of MdtF^{WT} and MdtF^{V610F} 34

Supplementary Fig. 19 | MdtF^{V610F} and R6G-MdtF^{V610F} structures exhibit MdtF^{WT}-like 'swung out' R2 state 35

Supplementary Fig. 20 | Monitoring MdtF thermal stability by CD and DSC 36

Supplementary Fig. 21 | Monitoring MdtF thermal stability by DSF 37

Supplementary Fig. 22 | RMSD fluctuations between MdtF and AcrB 38

Supplementary Fig. 23 | PC1-PC2 pseudo-contacts distribution analysis between monomers and proteins 39

Supplementary Fig. 24 | Conservation of proton relay network residues across MdtF structures 40

Supplementary Fig. 25 | Hydration at the proton relay site 41

Supplementary Fig. 26 | Water accessibility at the proton relay site 42

Supplementary Fig. 27 | Outer membrane pore expression differs between exponential and stationary phases 43

SECTION 4 REFERENCES 44

Section 1 Supplementary Discussion

Substrate docking at channels 1, 2 and 3 in MdtF compared to AcrB

Several residues surrounding putative drug channels in MdtF subtly differ from those in AcrB (**Supplementary Fig. 26**), which may affect substrate binding pocket access and specificity. Molecular docking for the same substrate set was performed at the putative channel entrances in MdtF and revealed an overall larger affinity to CH3 than to CH1 and CH2, (particularly for PAβN and ciprofloxacin) suggesting that CH3 may serve as a key entry route for these molecules. CH3 was identified in AcrB to preferentially export planar aromatic cations (PACs) which provides access via the central cavity[1]. Here, the compounds gain direct access to the DBP and bypass the PBP and switch loop. CH3 also exhibits channel entrance residue dissimilarities between AcrB and MdtF; AcrB (T37 and A100) and MdtF (Q37 and P100) (**Supplementary Fig. 14B**). The presence of a structurally disruptive proline and a polar molecule attracting glutamine may facilitate a better comprehension of the suggested preference of MdtF for cationic, flat, aromatic substances (such as dyes[2] and nitrosyl indoles[3]) in comparison to AcrB. To determine the difference in functionality owing to the CH3 entry gate, we tested the resistance phenotype of MdtF harbouring these mutations Q37T and P100A through implementing MIC assays against a panel of different drug types consisting of low and high molecular mass drugs (LMMD and HMMDs) and PACs (**Supplementary Fig. 14** and **Supplementary Table 4**). Although this demonstrated that the mutations did not significantly affect the MdtF resistance phenotype, the ability of MdtF to export PACs was corroborated due to its conferred resistance to crystal violet, doxorubicin, and R6G. Interestingly, both single-point mutants, $MdtF^{Q37T}$ and $MdtF^{P100A}$, displayed reduced resistance to the macrolide antibiotic erythromycin which was reversed in the presence of the double mutant $MdtF^{Q37T/P100A}$. This resistance phenotype effect arising from a combinatorial mutation indicates an epistatic interaction between these two residues. This genetic cooperativity could indicate a functional interdependency of these residues on the evolutionary trajectory of RND transporters in erythromycin-specific transport.

Section 2 Supplementary Tables

Supplementary Table 1 | Cryo-EM data collection, refinement, and validation statistics

	**Apo-MdtF^{WT}**	**Apo-MdtF^{V610F}**	**MdtF^{V610F}-R6G**
	**EMD-53281**	**EMD-53282**	**EMD-53283**
	**PDB 9QPR**	**PDB 9QPS**	**PDB 9QPT**
**Data collection and processing**			
Magnification	x 190,000	x 150,000	x 150,000
Voltage (keV)	200	200	200
Electron exposure (e^-/Å^2)	48.82	41.25	41.25
Defocus range (μm)	-0.8 to -2.2<br>(-0.2 steps)	-0.8 to -2.2<br>(-0.2 steps)	-0.8 to -2.2<br>(-0.2 steps)
Pixel size (Å)	0.946	0.947	0.947
Symmetry imposed	C1	C1	C1
Initial particle images (no.)	2,501,990	5,069,376	4,498,131
Final particle images (no.)	593,735	1,446,003	1,967,418
Map resolution (Å)	3.56	3.28	3.2
FSC threshold	0.143	0.143	0.143
**Refinement**			
Initial model used	Homology model	9QPR (this work)	9QPS (this work)
Model resolution (Å)	3.14	2.87	2.84
FSC threshold	0.143	0.143	0.143
Model resolution range (Å)	3.1-3.6	2.7-3.3	2.8-3.3
Map sharpening *B* factor (Å^2)	-194	-188	-177
Model composition			
Non-hydrogen atoms	23,906	24,004	24,062
Protein residues	3,051	3,056	3,054
Ligands	21 (PTY)<br>7 (D12)	22 (PTY)<br>11 (D12)	23 (PTY)<br>12 (D12)<br>1 (RHQ)
B factors (Å^2)			
Protein	38.22	37.30	44.01
Ligand	82.87	81.90	89.22
R.m.s. deviations			
Bond lengths (Å)	0.005	0.003	0.003
Bond angles (degrees)	0.668	0.556	0.540
Validation			
MolProbity score	2.00	1.76	1.70
Clashscore	11.25	8.47	8.62
Poor rotamers (%)	0.32	0.28	0.28
Ramachandran plot			
Favoured (%)	93.34	95.66	96.38
Allowed (%)	6.66	4.34	3.62
Disallowed (%)	0	0	0

Supplementary Table 2 | LC-MS lipid analysis of extracted MdtF SMALP.

Putative ID	*m/z*	Adduct	Mass accuracy (ppm)	RT (min)	Relative Abundance (x10^4)	ID confirmed by
PEs in MdtF SMALP						
PE 28:0	634.4472	$[M-H]^-$	3.0	7.78	28.83 ± 0.78	Accurate Mass
PE 30:1	660.4633	$[M-H]^-$	3.5	7.82	84.59 ± 2.35	Accurate Mass
PE 30:0	662.479	$[M-H]^-$	3.6	8.37	72.77 ± 3.30	PE 14:0_16:0 by HCD fragmentation
PE 31:1	674.4792	$[M-H]^-$	3.9	8.22	57.70 ± 0.41	Accurate Mass
PE 32:2	686.4793	$[M-H]^-$	3.9	7.86	61.18 ± 2.07	Accurate Mass
PE 32:1	688.4946	$[M-H]^-$	3.5	8.42	715.01 ± 8.24	PE 16:0_16:1 by HCD fragmentation
PE 33:2	700.4947	$[M-H]^-$	3.6	8.26	107.39 ± 4.55	PE 16:1_17:1 by HCD fragmentation
PE 33:1	702.5103	$[M-H]^-$	3.4	8.81	881.56 ± 16.57	PE 16:0_17:1 by HCD fragmentation
PE 34:2	714.5101	$[M-H]^-$	3.1	8.40	684.57 ± 6.26	PE 16:1_18:1 by HCD fragmentation
PE 34:1	716.5258	$[M-H]^-$	3.1	8.96	897.68 ± 11.58	PE 16:0_18:1 by HCD fragmentation
PE 35:2	728.526	$[M-H]^-$	3.3	8.76	549.19 ± 1.38	PE 17:1_18:1 by HCD fragmentation
PE 35:1	730.5416	$[M-H]^-$	3.1	9.35	284.43 ± 2.57	PE 16:0_19:1 by HCD fragmentation
PE 36:2	742.5418	$[M-H]^-$	3.5	8.92	836.89 ± 6.43	PE 17:1_19:1 by HCD fragmentation
PE 37:2	756.5574	$[M-H]^-$	3.3	9.31	213.0.2 ± 1.42	PE 18:1_19:1 by HCD fragmentation
PE 36:3	800.5479	$[M+OAc]^-$	3.9	8.27	24.02 ± 0.27	Accurate Mass
PGs in MdtF SMALP						
PG 30:1	691.4578	$[M-H]^-$	3.3	7.02	1.75 ± 0.05	Accurate Mass
PG 30:0	693.4732	$[M-H]^-$	2.9	7.62	1.63 ± 0.02	Accurate Mass
PG 31:1	705.4735	$[M-H]^-$	3.3	7.49	1.01 ± 0.003	Accurate Mass
PG 32:2	717.4738	$[M-H]^-$	3.6	7.09	1.67 ± 0.05	Accurate Mass
PG 32:1	719.4889	$[M-H]^-$	2.8	7.67	22.33 ± 0.35	Accurate Mass
PG 33:2	731.4893	$[M-H]^-$	3.3	7.54	3.31 ± 0.02	Accurate Mass
PG 33:1	733.5047	$[M-H]^-$	3.0	7.99	18.36 ± 0.16	Accurate Mass
PG 34:2	745.5045	$[M-H]^-$	2.7	7.67	26.34 ± 0.302	PG 16:1_18:1 by HCD fragmentation
PG 34:1	747.5199	$[M-H]^-$	2.4	8.13	37.44 ± 0.65	PG 16:0_18:1 by HCD fragmentation
PG 35:2	759.5201	$[M-H]^-$	2.5	7.99	14.51 ± 0.09	Accurate Mass
PG 35:1	761.5353	$[M-H]^-$	2.0	8.49	9.61 ± 0.04	Accurate Mass
PG 36:2	773.5358	$[M-H]^-$	2.6	8.12	41.44 ± 0.47	Accurate Mass
PG 37:2	787.5517	$[M-H]^-$	2.9	8.47	7.14 ± 0.05	Accurate Mass
CLs in MdtF SMALP						
CL 66:2	1375.968	$[M-H]^-$	2.4	11.33	128.91 ± 1.29	Accurate Mass
CL 68:3	1401.985	$[M-H]^-$	2.8	10.75	227.69 ± 2.63	Accurate Mass
CL 70:4	1428.001	$[M-H]^-$	3.4	11.29	140.04 ± 1.802	Accurate Mass
CL 70:3	1430.018	$[M-H]^-$	4.5	11.47	313.79 ± 2.02	Accurate Mass

Abundance values were calculated from three separate biological preparations of MdtF-SMALPs samples (*n* = 3).

Supplementary Table 3 | Molecular docking

	R6G		Linezolid		PAβN		Ciprofloxacin		Nitrosyl Indole	
	$MdtF^{WT}$	$MdtF^{V610F}$	$MdtF^{WT}$	$MdtF^{V610F}$	$MdtF^{WT}$	$MdtF^{V610F}$	$MdtF^{WT}$	$MdtF^{V610F}$	$MdtF^{WT}$	$MdtF^{V610F}$
**$DBP_{binding}$**	-8.3	-8.7	-7.5	-7.6	-10.2	-9.9	-6.0	-7.1	-6.0	-6.0
**$CH1_{binding}$**	-3.5	-3.8	-5.1	-5.3	-5.4	-5.3	-4.8	-4.8	-4.9	-4.9
**$CH2_{binding}$**	-3.6	-4.2	-5.1	-6.1	-4.2	-4.8	-4.6	-5.4	-4.3	-5.2
**$CH3_{binding}$**	-4.6	-5.4	-7.2	-6.2	-5.9	-6.2	-9.5	-7.8	-7.4	-8.0

Predicted binding affinities (kcal/mol) of various substrates to both $MdtF^{WT}$ and the $MdtF^{V610F}$ variant from the molecular docking calculations (see *Materials and Methods* for details). Binding affinities are reported for R6G, linezolid, PAβN, ciprofloxacin, and nitrosyl indole at three distinct binding sites: the distal binding pocket (DBP, based on the cryo-EM structure from this study), and the CH1/2/3 channel entrances (selected based on well-characterised corresponding AcrB residues). CH1: Channel 1; CH2: Channel 2, CH3: Channel 3, DBP: Distal binding pocket, PAβN (phenylalanine-arginine β-naphthylamide), R6G: Rhodamine 6G.

Supplementary Table 4 | MIC calculations of CH3 mutants

	Minimum Inhibitory Concentration (MIC, μg mL^{-1})											
	Low Molecular Mass Drugs (LMMDs)						High Molecular Mass Drugs (HMMDs)			Planar Aromatic Cations (PACs)		
	Ciprofloxacin	Deoxycholate	Linezolid	PAβN	SDS	Tetracycline	Erythromycin	Novobiocin	Rifampicin	Rhodamine 6G	Crystal Violet	Doxorubicin
**MdtF^{WT}**	0.0078	1250	2	125	205	0.0975	100	0.78	6.24	800	0.04	>200
**MdtF^{Q37T}**	0.0019	1250	2	125	205	0.04875	50	3.12	1.56	800	0.04	>200
**MdtF^{P100A}**	0.0019	1250	2	125	205	0.0975	25	3.12	1.56	800	0.04	>200
**MdtF$^{Q37T/P100A}$**	0.0019	1250	2	125	205	0.0975	100	3.12	1.56	800	0.04	>200
**Empty pUC19**	0.0019	<78	1	31.25	25.625	0.04875	0.78	0.39	6.24	3.125	<0.02	1.56

Supplementary Table 5 | Primer table

Name	Sequence (5' - 3')	Use
sGFP_Del_Fwd	CTC GAG CAC CAC CAC CAC	Deletion of TEV-sGFP region using Q5® mutagenesis kit from pET15b-MdtF-sGFP-6xHis plasmid; forward primer
sGFP_Del_Rev	GCT CTG GAA GTA CAG GTT TTC AC	Deletion of TEV-sGFP region using Q5® mutagenesis kit from pET15b-MdtF-sGFP-6xHis; reverse primer
MdtF_V610F_Fwd	GGT GTT TAC CTT TGG CGG CTT TG	Q5® mutagenesis of V610F in *mdtF* gene, reverse primer
MdtF_V610F_Rev	GAC TGG ACA TTA TCT TTC TCT TTA GTC	Q5® mutagenesis of V610F in *mdtF* gene, forward primer
pUC19_MdtEF_Fwd	GCC AAG CTT GCA TGC CTG CAG AAC TGT TGG CAG AAC GG	Cloning *mdtEF* genes with its natural promoter from K-12 *Escherichia coli* chromosomal DNA into a pUC19 plasmid (linearised with *Pst*I and *Bam*HI restriction enzymes); forward primer
pUC19_MdtEF_His_Rev	ATT CGA GCT CGG TAC CCG GGT CAG TGG TGG TGG TGG TGG TGC TCG AGG CTC TGG AAG TAC AGG TTT TCA CCG CTA GCC GCT TTT TTA AAG CGG GC	As delineated above, however, a 6xHistidine tag sequence was included in the reverse primer to provide a 6xHis tag at the C-terminus of MdtF; reverse primer
pUC19_MdtEF_Tag_Del_Fwd	TGA CCC GGG TAC CGA GCT	Deletion of TEV-6xHis region using Q5® mutagenesis kit from puC19-MdtEF-TEV-6xHis plasmid; forward primer
pUC19_MdtEF_Tag_Del_Rev	CGC TTT TTT AAA GCG GGC AAA GAG	Deletion of TEV-6xHis region using Q5® mutagenesis kit from puC19-MdtEF-TEV-6xHis plasmid; reverse primer
MdtF_Q37T_Fwd	GCA GTA TCC GAC GAT TGC GCC AC	Q5® mutagenesis of Q37T in *mdtF* gene, forward primer
MdtF_Q37T_Rev	GCA ACC GGT AAG TTC ATG	Q5® mutagenesis of Q37T in *mdtF* gene, reverse primer
MdtF_P100A_Fwd	TGG GAC ATC TGC GGA TAT CGC AC	Q5® mutagenesis of P100A in *mdtF* gene, forward primer
MdtF_P100A_Rev	GTC TCG AAG GTC AGA GTG	Q5® mutagenesis of P100A in *mdtF* gene, reverse primer
MdtF_D408A_Fwd	GTT GGT GGA TGC GGC CAT CGT TGT GG	Q5® mutagenesis of D408A in *mdtF* gene, forward primer
MdtF_D408A_Rev	AGG CCG ATG GCG AGC	Q5® mutagenesis of D408A in *mdtF* gene, reverse primer
Q37T_Seq_Rev	TGC TCG ACT TAT CGA CGC TAA TC	Sequencing of Q37T mutation; reverse primer
P100A_Seq_Fwd	AGG TGG TCT GGC GAT CAT GAA C	Sequencing of P100A mutation; forward primer
V610F_Seq_Fwd	ATC GCC ATC AAA CTG GCT GC	Sequencing of V610F mutation; forward primer
MdtF_Mid_Seq_Fwd	GAA CTG AAC CGC TTA TCA GC	Sequencing of MdtF middle region; forward primer
MdtF_Mid_Seq_Rev	AAC CAA CCT GGA AGT AGA CG	Sequencing of MdtF middle region; reverse primer
T7 _Seq_Fwd	TAA TAC GAC TCA CTA TAG GG	Sequencing downstream from T7 promoter for pET15b plasmids; forward primer
T7_term_Seq_Rev	CTA GTT ATT GCT CAG CGG T	Sequencing upstream from T7 terminator for pET15b plasmids; reverse primer
M13_Seq_Fwd	GTT TTC CCA GTC ACG AC	Sequencing N-terminus of inserts for pUC19 plasmids; forward primer
M40_Seq_Rev	CGG ATA ACA ATT TCA CAC AG	Sequencing N-terminus of inserts for pUC19 plasmids; reverse primer

Supplementary Table 6 | Structural and compositional details of MdtF^{WT}, MdtF^{V610F}, and AcrB systems

System	POPE:POPG	Water Molecules	Box Dimensions (Å^3)	Total Atoms
MdtF^{WT}	463:231	270861	168.7 × 168.5 × 174.3	405,380
MdtF^{V610F}	503:252	305736	179.5 × 181.5 × 182.8	447,955
AcrB	433:217	248208	168.7 × 168.5 × 174.3	377,728

Supplementary Table 7 | Global RMSD (in Å) comparisons of MdtF access, binding, and extrusion states (Cα-atoms) to published RND structures

Protein (membrane mimetic)	PDB ID	Reference	Conformation	RMSD MdtF (Å)		
				Access	Binding	Extrusion
AcrB (DDM micelle)	4DX5	Eicher *et al.* (2012)[4]	Access	1.6	2.4	2.8
			Binding	1.9	1.1	3.0
			Extrusion	2.5	2.6	1.0
AcrB (SMALPs)	6BAJ	Qiu *et al.* (2018)[5]	Access	2.3	3.1	3.1
			Binding	2.0	1.3	3.5
			Extrusion	2.7	3.3	1.5
AcrD (MSP *E. coli* lipid nanodisc)	8F4R	Zhang *et al.* (2023)[6]	Resting	1.8	2.5	2.6
			Binding	2.3	1.5	3.7
			Extrusion	3.2	3.7	1.8
OqxB (DDM micelle)	7CZ9	Bharatham *et al.* (2021)[7]	Binding (A)	2.4	2.1	2.9
			Binding (B)	2.4	2.2	2.6
			Binding (C)	2.3	2.2	2.6
AdeB (MSP *E. coli* lipid nanodisc)	7KGI	Morgan *et al.* (2021)[8]	Access	1.8	2.2	2.6
			Binding	2.6	2.2	3.4
			Extrusion	3.3	3.6	2.1
MexB (LMNG or C7NG micelle)	6IIA	Sakurai *et al.* (2019)[9]	Access	2.0	2.7	2.7
			Binding	2.4	1.8	3.4
			Extrusion	2.8	3.3	1.4
CusA (CYMAL-6 micelle)	3K0I	Long *et al.* (2010)[10]	Access	4.6	4.2	5.7
	3K07	Long *et al.* (2010)[10]	Extrusion	4.3	4.8	3.9

Supplementary Table 8 | MD simulations checklist

**Reliability and reproducibility checklist for molecular dynamics simulations** ***All boxes must be marked YES by acceptance unless “Response not needed if No”.**	**Yes**	**No**	**Response (Please state where this information can be found in the text)**
**1. Convergence of simulations and analysis**			
1a. Is an evaluation presented in the text to show that the property being measured has equilibrated in the simulations (*e.g.* time-course analysis)?	☒	☐	Yes. The measured properties, including pseudo-contacts, were calculated from well-equilibrated trajectories. The protein backbone RMSD remained stable and plateaued around 3 Å throughout the production phase, with no significant fluctuations, indicating convergence and reliable sampling of the relevant conformational space (Supplementary Fig. 22).
1b. Then, is it described in the text how simulations are split into equilibration and production runs and how much data were analyzed from production runs?	☒	☐	Simulation details are described in detail in sections ‘Molecular Docking’ and Molecular Dynamics Simulations’ of the Methods.
1c. Are there at least 3 simulations per simulation condition with statistical analysis?	☒	☐	3 independent replicas, each of 1 μs in length, were run for each system.
1d. Is evidence provided in the text that the simulation results presented are independent of initial configuration?	☒	☐	Yes, the details are described in sections ‘Molecular Docking’ and Molecular Dynamics Simulations’ of the Methods.
**2. Connection to experiments**			
2a. Are calculations provided that can connect to experiments (*e.g.* loss or gain in function from mutagenesis, binding assays, NMR chemical shifts, J-couplings, SAXS curves, interaction distances or FRET distances, structure factors, diffusion coefficients, bulk modulus and other mechanical properties, *etc.*)?	☒	☐	Yes. Docking and MD simulations were used to interpret the experimentally observed functional changes in MdtF due to

			the V610F mutation. Simulations of AcrB, $MdtF^{WT}$, and $MdtF^{V610F}$ revealed differences in cleft dynamics linked to susceptibility. Docking supported plausible ligand entry routes consistent with prior experimental findings.
**3. Method choice**			
3a. Do simulations contain membranes, membrane proteins, intrinsically disordered proteins, glycans, nucleic acids, polymers, or cryptic ligand binding?	☒	☐	Inner Membrane (POPE/POPG) with membrane protein of bacterial RND efflux pump.
3b. Is it described in the text whether the accuracy of the chosen model(s) is sufficient to address the question(s) under investigation (e.g. all-atom vs. coarse-grained models, fixed charge vs. polarizable force fields, implicit vs. explicit solvent or membrane, force field and water model, etc.)?	☒	☐	Yes, an all-atom model employing the AMBER ff19SB force field, OPC water model, and explicit lipid membrane model (lipid21 force field) were chosen to describe with high accuracy protein dynamics.
3c. Is the timescale of the event(s) under investigation beyond the brute-force MD simulation timescale in this study that enhanced sampling methods are needed?	☒	☐	The conformational dynamics explored in this study are within the timescale accessible to conventional molecular dynamics simulations. Our focus is on comparing the structural flexibility of MdtF wildtype, its V610F mutant, and AcrB under equilibrium conditions and at specific domains or domain interfaces.
If **YES**, are the parameters and convergence criteria for the enhanced sampling method clearly stated?	☐	☒	
If **NO,** is the evidence provided in the text?	☒	☐	Computational details are described in detail in the methods

				according to the rationale outlined above.
**4. Code and reproducibility**				
4a. Is a table provided describing the system setup that includes simulation box dimensions, total number of atoms, total number of water molecules, salt concentration, lipid composition (number of molecules and type)?		☒	☐	Yes, it is described in computational methods (Supplementary Table 6).
4b. Is it described in the text what simulation and analysis software and which versions are used?		☒	☐	Computational details are described in detail in the methods.
4c. Are other parameters for the system setup described in the text, such as protonation state, type of structural restraints if applied, nonbonded cutoff, thermostat and barostat, etc.?		☒	☐	All this information is reported in the methods.
4d. Are initial coordinate and simulation input files and a coordinate file of the final output provided as supplementary files or in a public repository?		☒	☐	MD trajectories have been made available in zenodo.
4e. Is there custom code or custom force field parameters?		☐	☒	Response not needed if **No**
	If **YES**, are they provided as supplementary files or in a public repository?	☐	☐	

Section 3 Supplementary Figures

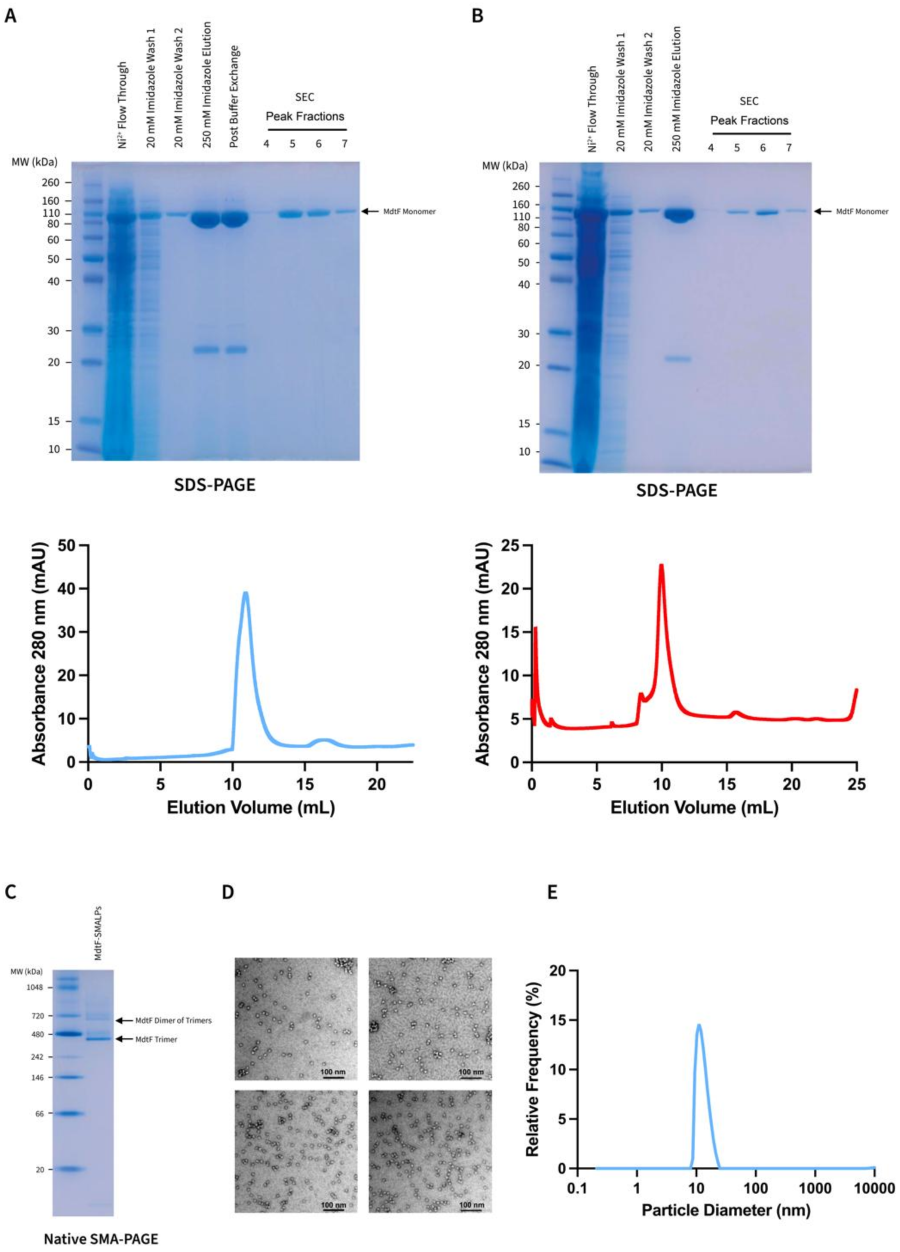

Supplementary Fig. 1 | Expression, purification, and characterisation of MdtF in SMALPs. a and b, Representative SDS-PAGE gels and corresponding SEC traces of MdtF^{WT} **(a)** and MdtF^{V610F} **(b)**. The MdtF monomer at ~111 kDa is indicated on the SDS-PAGE gel images. **c,** Native SMA-PAGE gel demonstrating the purification of the MdtF homo-trimer within SMALPs, indicated on the gel image. A dimer of the MdtF homo-trimer is also observed due to electrostatic interactions between the SMA nanodisc. **d,** Negative stain EM analysis of MdtF-SMALPs, demonstrating particle monodispersity and homogeneity. Here, the particle size is observed to be ~13 nm, corresponding to the size of AcrB. **e,** Representative DLS trace of MdtF-SMALPs, showing particle homogeneity at approximately 13 nm in accordance with the size observed by negative stain EM analysis.

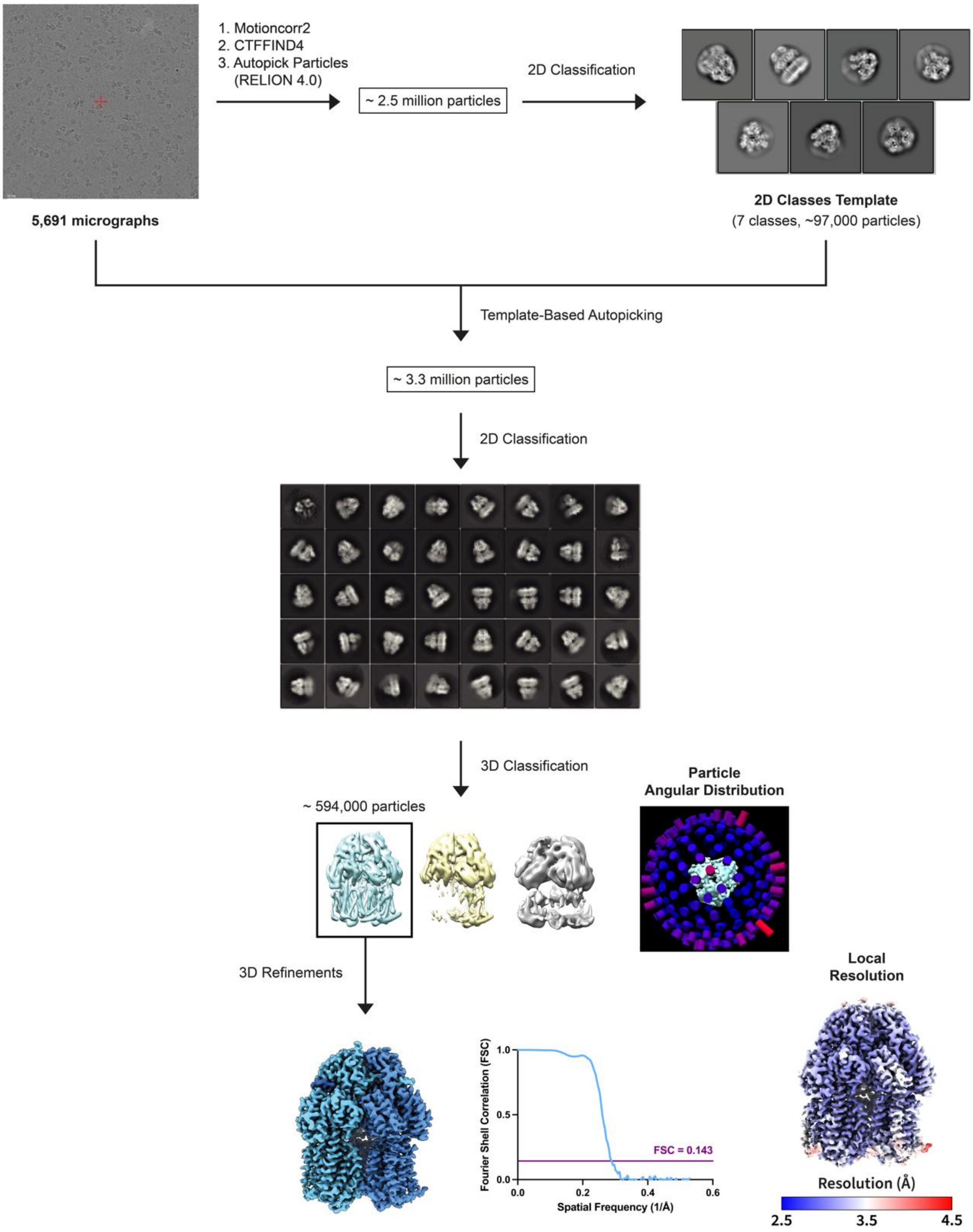

Supplementary Fig. 2 | MdtF^{WT} cryo-EM data processing. An outline of the cryo-EM data processing workflows for MdtF^{WT}-SMALPs that are described within the Methods. In brief, 2 rounds of 2D classification were undertaken and the classes which best represented the MdtF^{WT}-SMALP particle were taken forward for 3D classification. Here, the class harbouring the best-looking particle stack were then refined and polished in RELION. The final map was then obtained by refinement of the polished particles within RELION which were used for model building. FSC curves of the final 3D reconstruction calculated in Relion-4.0 is also demonstrated (3.56 Å). A representative micrograph with the physical pixel size (50 nm) is displayed. FSC: Fourier shell correlation. The representative angular distribution of particles and the final map coloured by local resolution is also displayed.

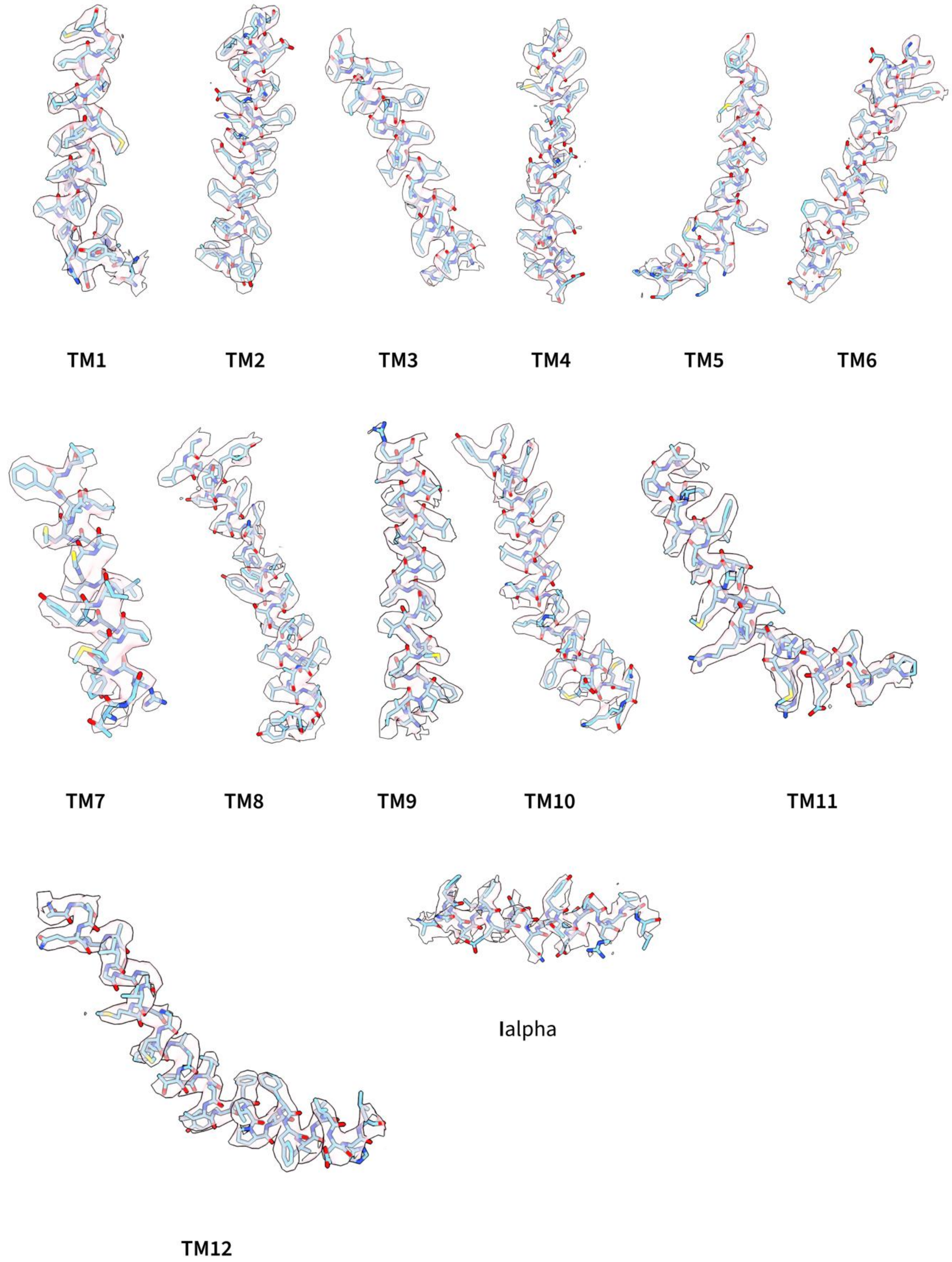

Supplementary Fig. 3 | Quality of MdtF^{WT} cryo-EM map. Representative cryo-EM densities (surface form, light pink) for the TM helices of MdtF^{WT}. The MdtF^{WT} structure is depicted in atom representation (light blue). TM: transmembrane helix.

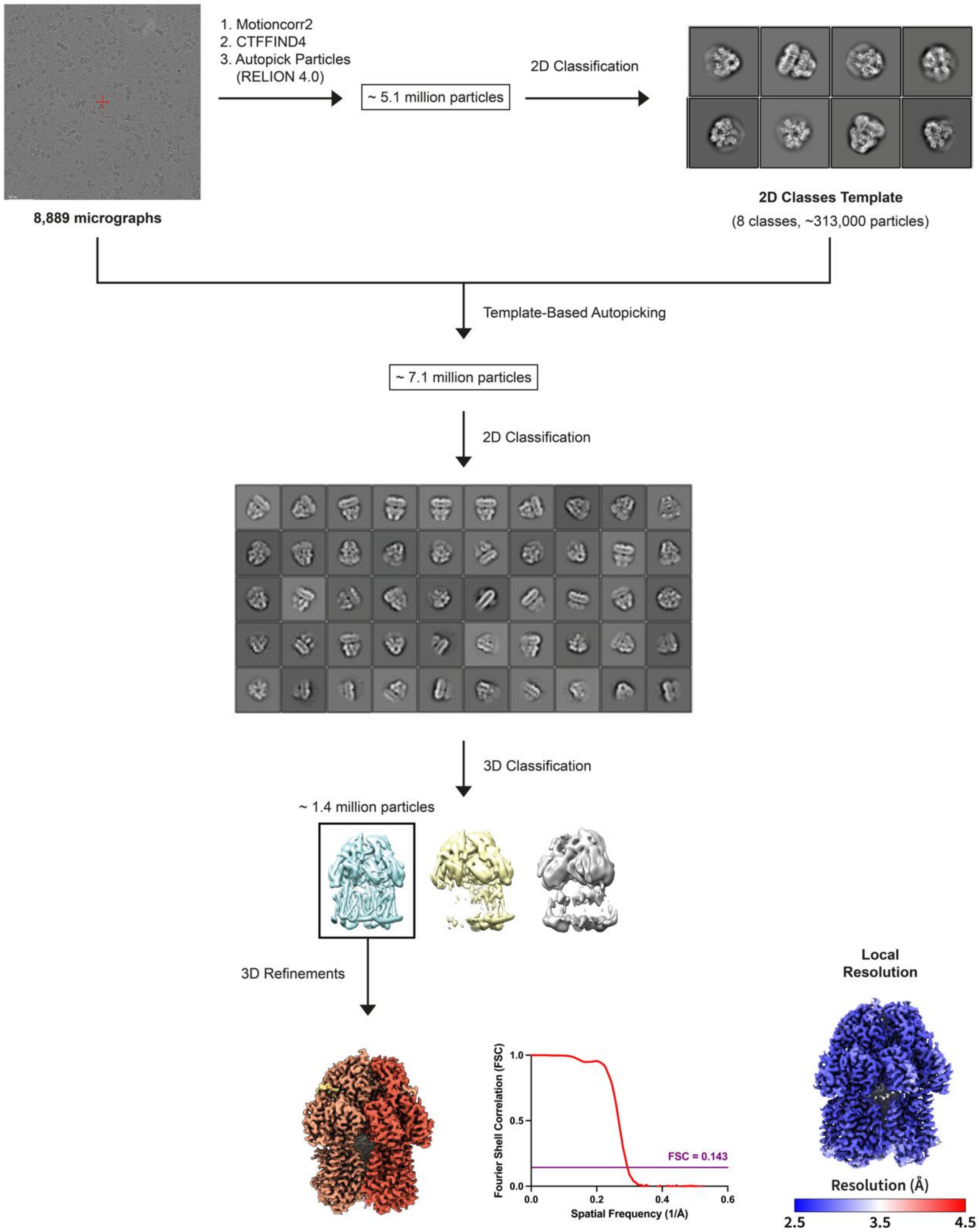

Supplementary Fig. 4 | MdtF^{V610F} cryo-EM data processing. An outline of the cryo-EM data processing workflows for MdtF^{V610F}-SMALPs that are described within the Methods. In brief, 2 rounds of 2D classification were undertaken and the classes which best represented the MdtF^{V610F}-SMALP particle were taken forward for 3D classification. Here, the class harbouring the best-looking particle stack were then refined and polished in RELION. The final map was then obtained by refinement of the polished particles within RELION which were used for model building. FSC curves of the final 3D reconstruction calculated in Relion-4.0 is also demonstrated (3.28 Å). A representative micrograph with the physical pixel size (50 nm) is displayed. FSC: Fourier shell correlation. The final map coloured by local resolution is also displayed.

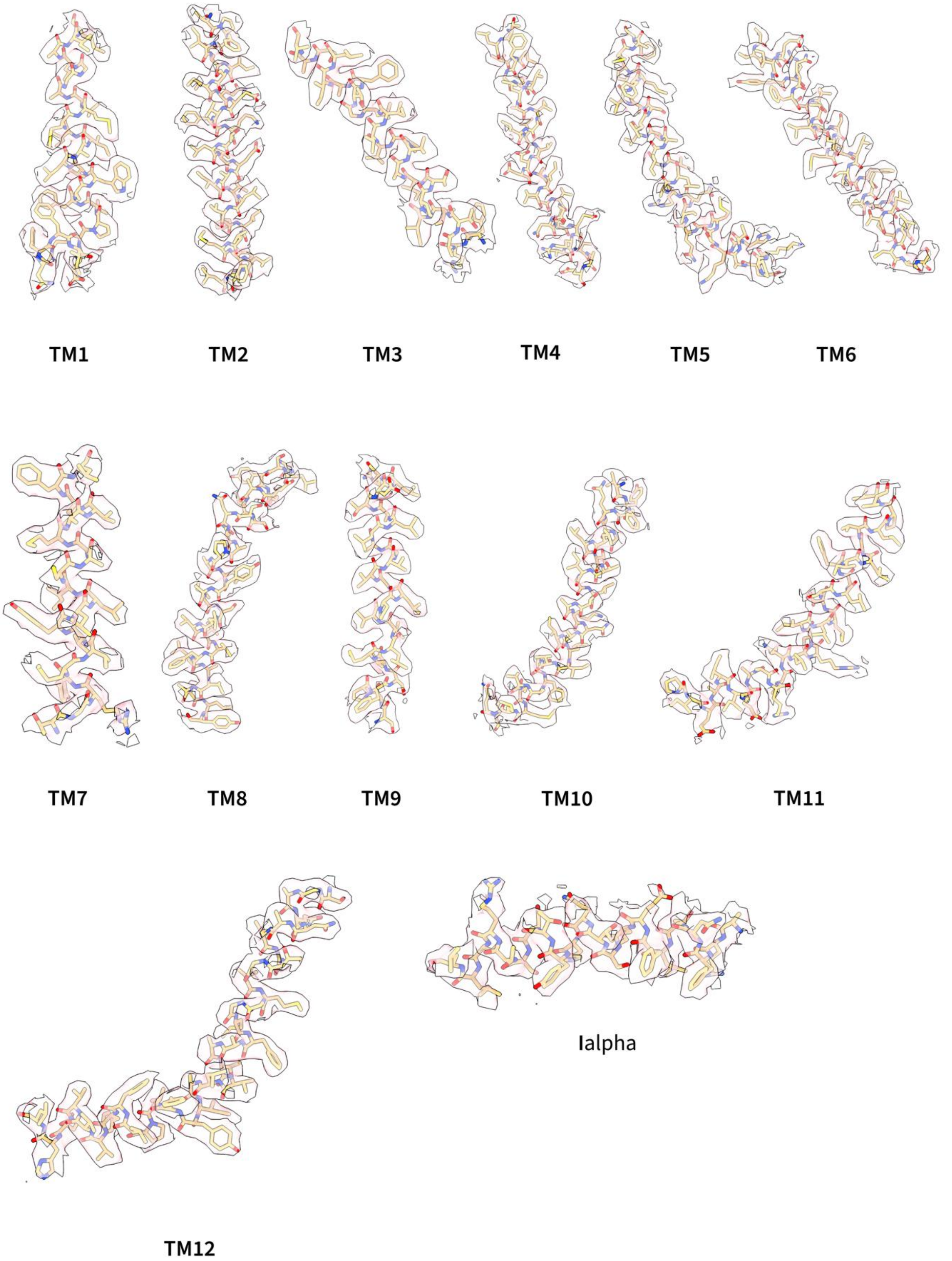

Supplementary Fig. 5 | Quality of MdtF^{V610F} cryo-EM map. Representative cryo-EM densities (surface form, light pink) for the TM helices of MdtF^{V610F}. The MdtF^{V610F} structure is depicted in atom representation (light orange). TM: transmembrane helix.

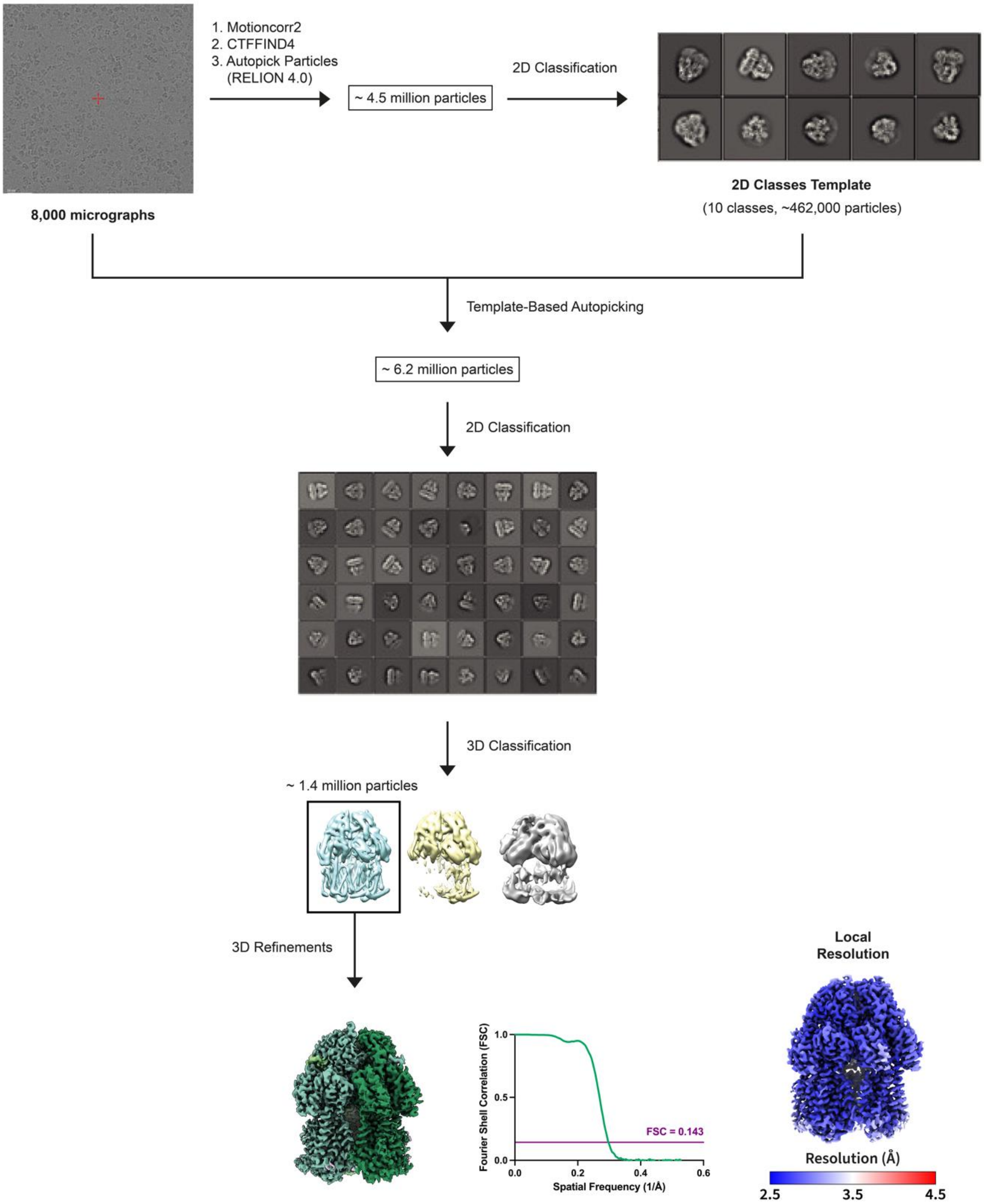

Supplementary Fig. 6 | MdtF^{V610F}-R6G cryo-EM data processing. An outline of the cryo-EM data processing workflows for R6G-bound MdtF^{V610F}-SMALPs that are described within the Methods. In brief, 2 rounds of 2D classification were undertaken and the classes which best represented the MdtF^{V610F}-SMALP particle were taken forward for 3D classification. Here, the class harbouring the best-looking particle stack were then refined and polished in RELION. The final map was then obtained by refinement of the polished particles within RELION which were used for model building. FSC curves of the final 3D reconstruction calculated in Relion-4.0 is also demonstrated (3.20 Å). A representative micrograph with the physical pixel size (50 nm) is displayed. FSC: Fourier shell correlation. The final map coloured by local resolution is also displayed.

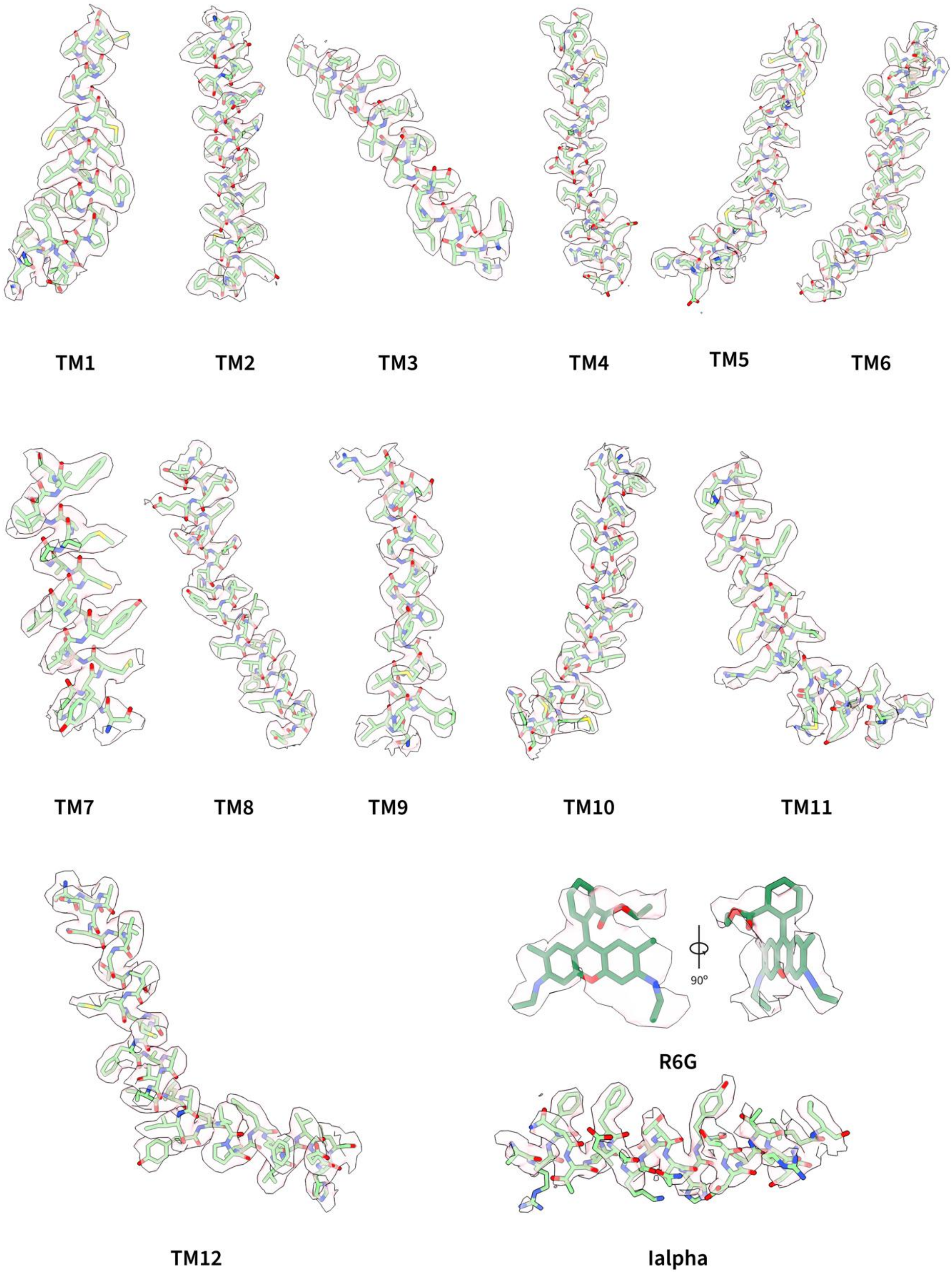

Supplementary Fig. 7 | Quality of MdtF^{V610F}-R6G cryo-EM map. Representative cryo-EM densities (surface form, light pink) for the TM helices of MdtF^{V610F}-R6G. The MdtF^{V610F}-R6G structure is depicted in atom representation (light green). TM: transmembrane helix.

A

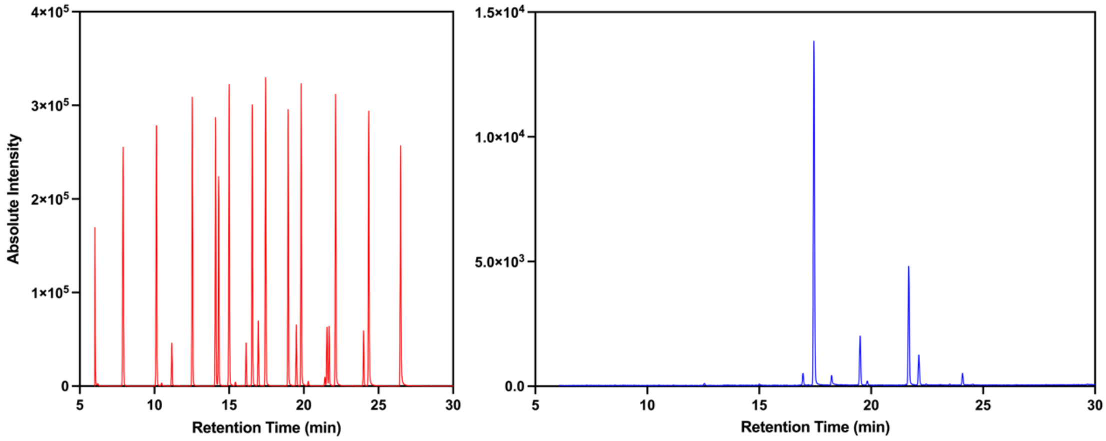

B

Lipid number	Fatty acid methyl ester	Fatty acid type	Carbon number	Percentage abundance (%)	Retention time (min)
14	Methyl hexadecenoate (*cis*-9) (palmitoleate)	UFA	C16:1	1.67 ± 0.48	16.95
15	Methyl hexadecanoate (palmitate)	SFA	C16:0	23.16 ± 0.89	17.45
17	Methyl cis-9,10-methylenehexadecanoate	CFA	C17:0	18.43 ± 0.26	19.83
18	Methyl heptadecanoate (margarate)	SFA	C17:0	0.25 ± 0.05	20.30
22	Methyl octadecenoate (*trans*-9) (elaidate)	UFA	C18:1	48.75 ± 1.43	21.70
23	Methyl octadecanoate (stearate)	SFA	C18:0	2.22 ± 0.32	22.13
24	Methyl cis-9,10-methyleneoctadecanoate	CFA	C19:0	5.51 ± 0.05	24.35

C

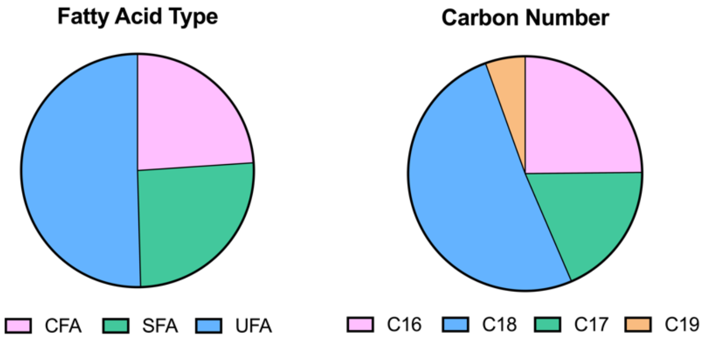

Supplementary Fig. 8 | Lipidomic analysis of MdtF-SMALPs lipid belt. a, Representative chromatograms obtained for the Bacterial Acid Methyl Ester (BAME) Mix standard (Sigma Aldrich) (top panel) and lipids extracted from the SMALP-extracted MdtF sample. Composition of the BAME mix is: Methyl undecanoate, Methyl (±)-2-hydroxydecanoate, Methyl dodecanoate, Methyl tridecanoate, Methyl 2-hydroxydodecanoate, Methyl (±)-3-hydroxydodecanoate, Methyl myristate, Methyl 13-methyltetradecanoate, Methyl 12-methyltetradecanoate, Methyl pentadecanoate, Methyl 2-hydroxytetradecanoate, Methyl 3-hydroxytetradecanoate, Methyl 14-methylpentadecanoate, Methyl cis-9-hexadecenoate, Methyl palmitate, Methyl 15-methylhexadecanoate, Methyl cis-9,10-methylenehexadecanoate, Methyl heptadecanoate, Methyl 2-hydroxyhexadecanoate, Methyl linoleate, Methyl oleate, Methyl trans-9-octadecenoate, Methyl stearate, Methyl methyleneoctadecanoate (all cis-9,10), Methyl nonadecanoate, and Methyl eicosenoate. **c,** Lipidomic analysis of lipid head group composition within the purified MdtF-SMALP nanodics. **b,** Lipids identified by GC-mS analysis within the SMALP nanodisc. The associated fatty acyl chain type, carbon number, percentage abundance, and retention time are also

displayed. **c,** GC-MS analysis of fatty acyl chain and carbon number composition within the purified MdtF-SMALP nanodisc. Pie charts represent mean values from three independent measurements (n = 3). CFA: cyclopropanated fatty acid, CL: cardiolipin, FAME: fatty acid methyl ester, GC-MS: gas-chromatography mass spectrometry, PE: phosphatidylethanolamine, PG: phosphatidylglycerol, SFA: saturated fatty acid, SMALP: styrene maleic acid lipid polymer, UFA: unsaturated fatty acid.

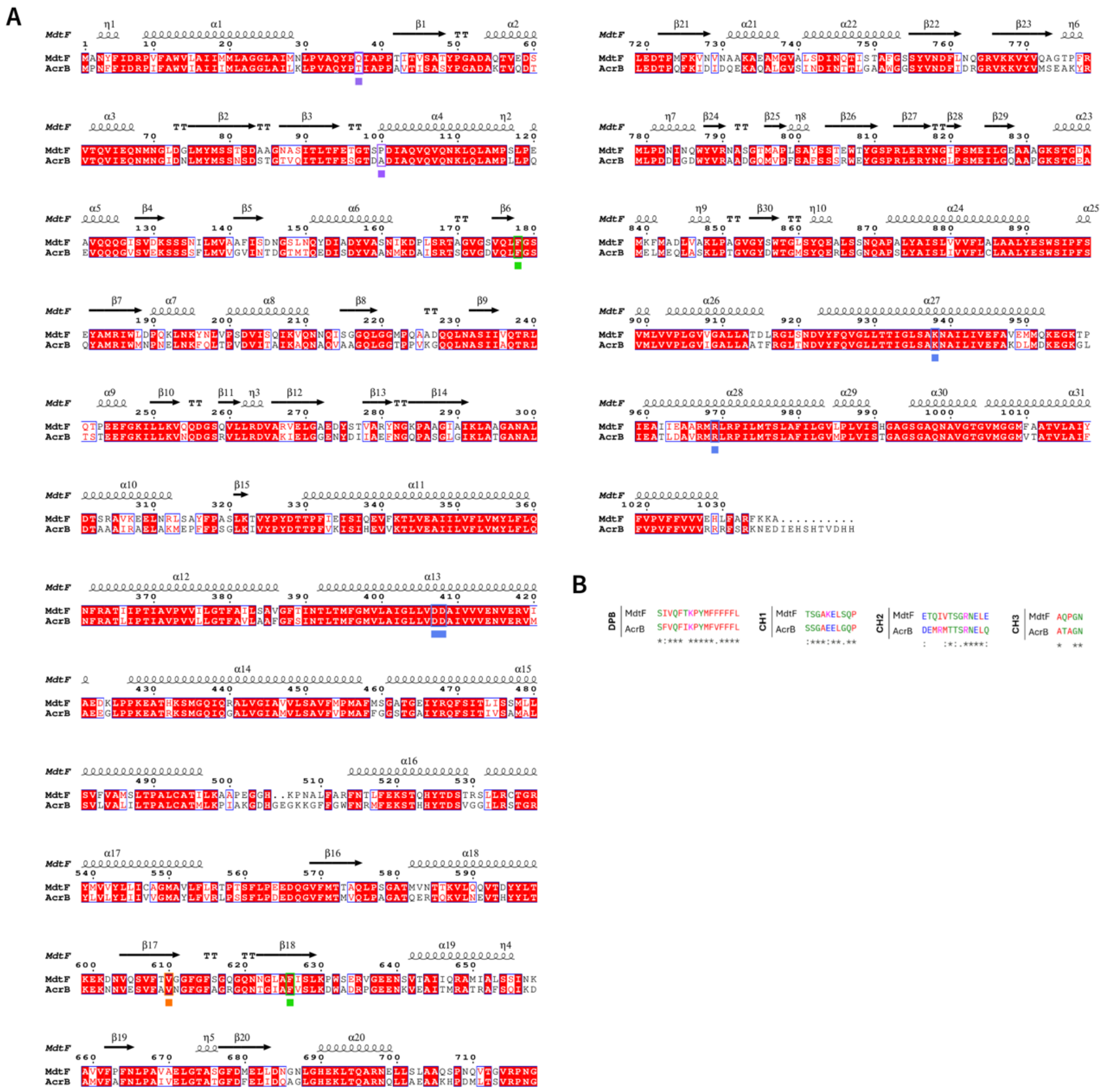

Supplementary Fig. 9 | Multiple sequence alignment of MdtF and AcrB. a, The sequence alignment was performed using ESPript (ENDscript)[11]. The residue numbering is shown for both MdtF. MdtF-related secondary structure specific to particular amino acid residues is also demonstrated. The location of residues which have been observed to be important in the proton relay network, R6G-binding, and gate the channel 3 entrance are indicated with a box with the colours blue, green, and purple, respectively. The residue V610 is also indicated with a box with the colour orange. Red framed boxes enclosing both residues of MdtF and AcrB indicate sequence conservation. Dots indicate a gap in the sequence to highlight residue discrepancies between the two proteins. Sequences were obtained from UniProt, Accession codes: P31224 (AcrB) and P37637 (MdtF). **b,** Sequence alignment of the binding site and putative channel entrance regions for MdtF and AcrB. ***DPB_MdtF (DPB_AcrB)*** residues include S134 (S134), I136 (F136), V139 (V139), Q176 (Q176), F178 (F178), T277 (I277), K292 (K292), P326 (P326), Y327 (Y327), M571 (M573), F608 (F610), F610 (V612), F613 (F615), F615 (F617), F626 (F628), L666 (L668). ***CH1_MdtF (CH1_AcrB)*** residues include T559 (S561), S834 (S836), G836 (G838), A838 (A840), K840 (E842), E864 (E866), L866

(L868), S868 (G870), Q870 (Q872), P872 (P874); ***CH2_MdtF (CH2_AcrB)*** residues include E564 (D566), T643 (E645), Q647 (M649), I651 (R653), V660 (M662), T674 (T676), S676 (T678), G713 (S715), R715 (R717), N717 (N719), E720 (E722), L826 (L828), E828 (Q830); and ***CH3_MdtF (CH3_AcrB)*** residues include A33 (A33), Q37 (T37), P100 (A100), G296 (G296), N298 (N298). Sequence alignment was performed using Clustal Omega[12].

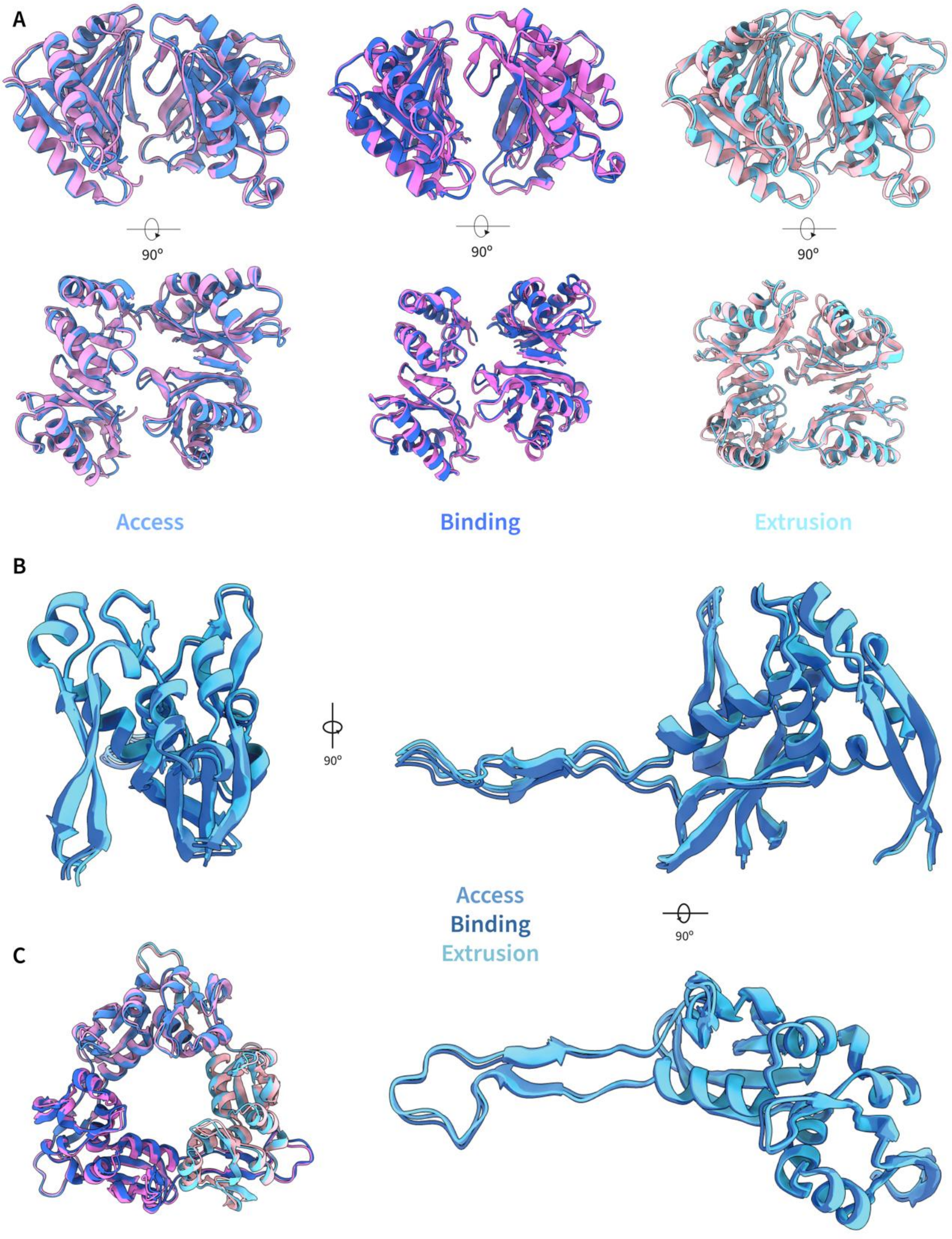

Supplementary Fig. 10 | Porter domain conformation comparison between MdtF^{WT} and AcrB. **a,** MdtF^{WT} (blue) and AcrB (PDB: 2HRT[13], pink) alignment of porter domain in each of the access, binding, and extrusion states occupied during a drug binding event. A similar conformational change is observed between states in both structures. **b,** The funnel domain structure within each of the monomeric states of MdtF^{WT} were aligned and demonstrate an overall structural conservation between each protomer. **c,** In comparison to AcrB (pink), MdtF^{WT} (blue) adopts a similar funnel domain structure.

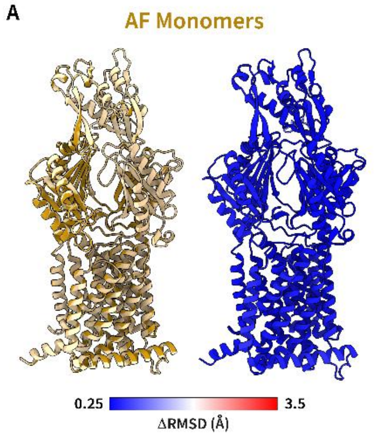

B

ΔRMSD (Å)		
AF Monomer		
1 vs 2	2 vs 3	3 vs 1
0.10	0.11	0.10

C

	ΔRMSD (Å)		
	Access	Binding	Extrusion
AF Monomer	1.6	1.2	2.5

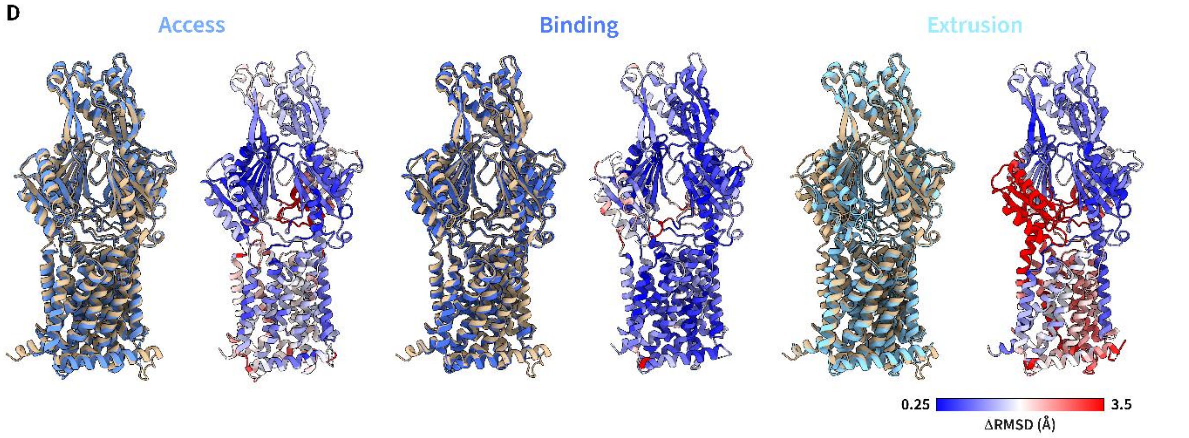

Supplementary Fig. 11 | Assessment of AlphaFold Prediction of MdtF Structures. a, Overlaying the 3 monomers of the homo-trimeric MdtF predicted by AlphaFold 3[14] demonstrates the absence of asymmetry which is functionally important for RND-based transporters. **b,** Calculations of ΔRMSD measurements between each monomer of the AlphaFold-predicted MdtF (namely, AF monomer 1, 2, and 3) structure further highlights the similarity between each of the monomers (~0.1). **c,** Comparisons of the AlphaFold-predicted structure of MdtF to each of the protomers identified within our cryo-EM structure of $MdtF^{WT}$ shows the disparity between these predicted and experimentally derived models. **d,** ΔRMSD measurements between each protomeric state of $MdtF^{WT}$ and the AlphaFold-predicted states consolidates the inability to predict this asymmetric structure observed experimentally.

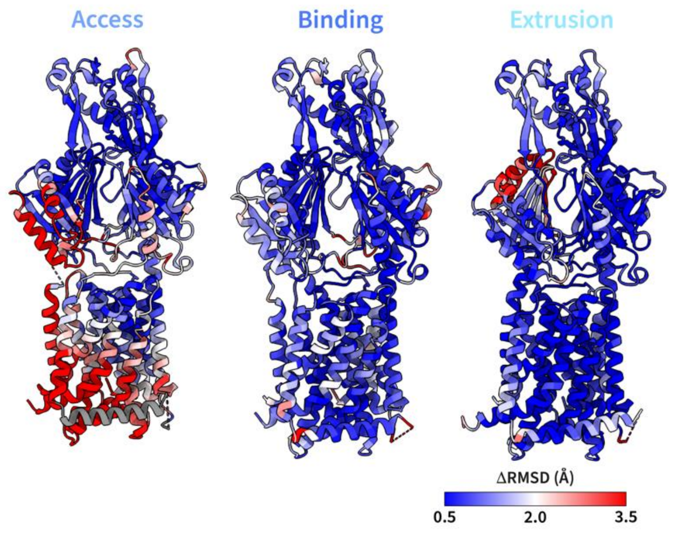

Supplementary Fig. 12 | MdtF^{WT} demonstrates stationary transmembrane domain compared to AcrB-SMALPs. ΔRMSD measurements between each protomeric state of MdtF^{WT} and AcrB purified in SMALPs corroborates differences localised to the transmembrane domain within the access protomer. The Iα helix of the AcrB-SMALPs structure (6BAJ)[5] was not resolved and therefore represented in grey.

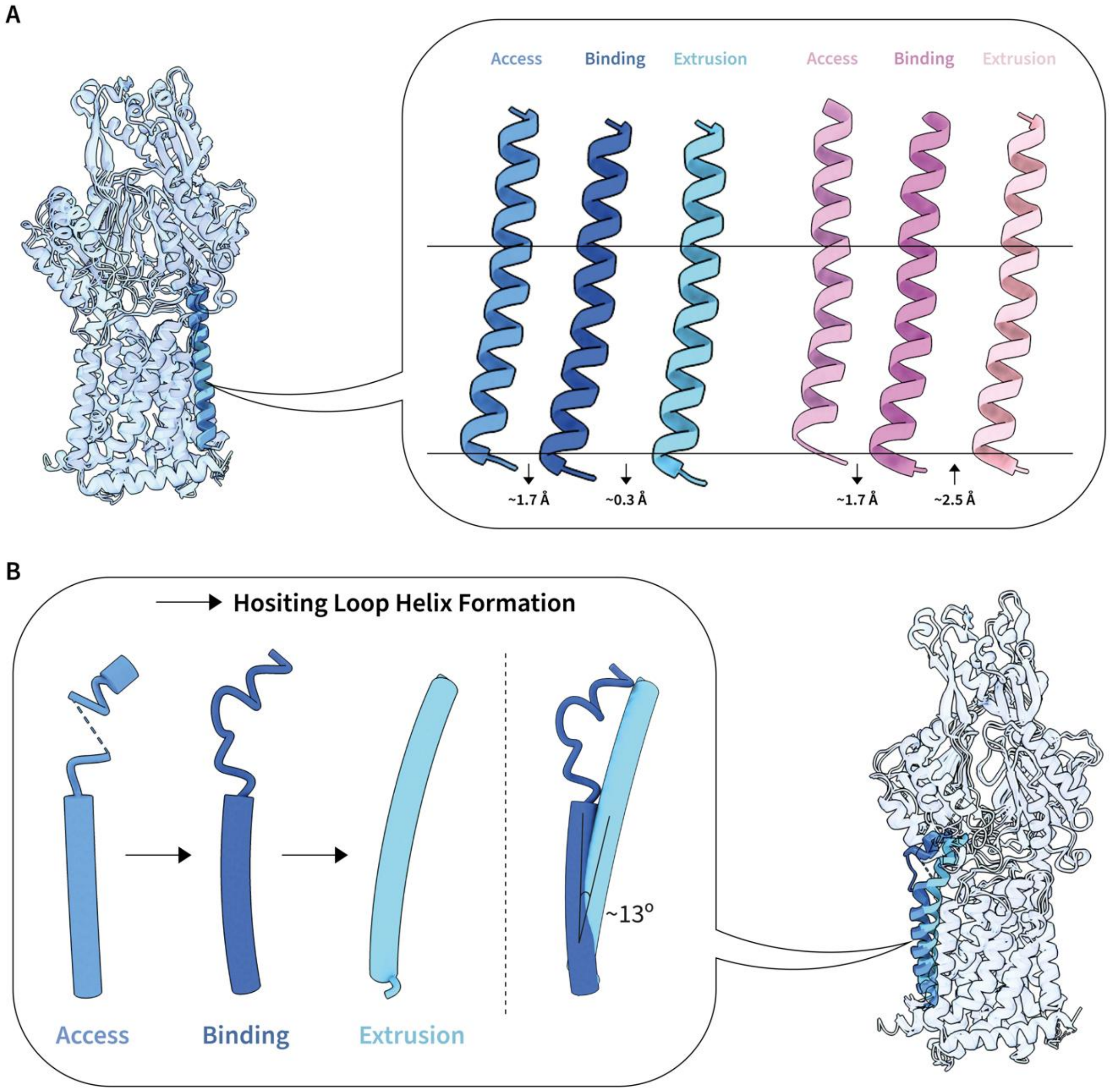

Supplementary Fig. 13 | Structural transitions observed in TM2 and TM8. a, The vertical transitions between access, binding, and extrusion states in AcrB and MdtF^{WT} as measured from its distance from the inner membrane. MdtF^{WT} undergoes a reduced vertical transition between the binding and extrusion states. **b,** The formation of a helical structure in the hoisting loop of TM2 from access to binding to extrusion is conserved within our MdtF^{WT} structure.

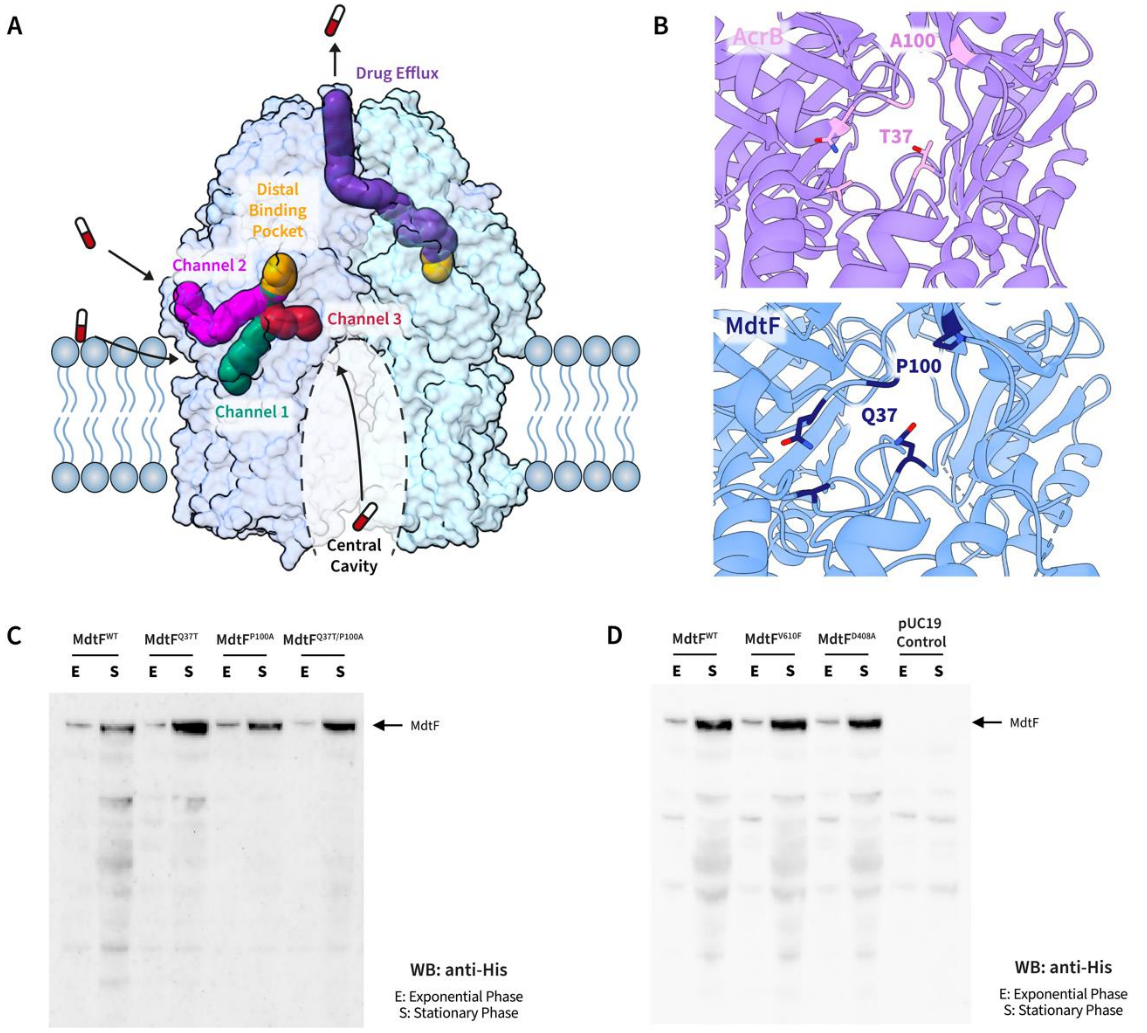

Supplementary Fig. 14 | MdtF^{WT} channels of export and channel 3 entrance. a, The RND-related channels of MdtF^{WT} as calculated by MOLE[15]. **b,** The residues gating the channel entrance exhibit variations between MdtF^{WT} (Q37 and P100, blue) and AcrB (T37 and A100, pink). **c and d,** MdtF mutant expression tests for MIC and Nile Red efflux assays. Here, cell lysates of *E. coli* Δ9-Pore cells expressing MdtF mutants at exponential and stationary phase were separated by SDS-PAGE. Gels were transferred to a nitrocellulose membrane and subsequently immunoblotted with anti-His antibodies. CH3: Channel 3, WB: Western blot.

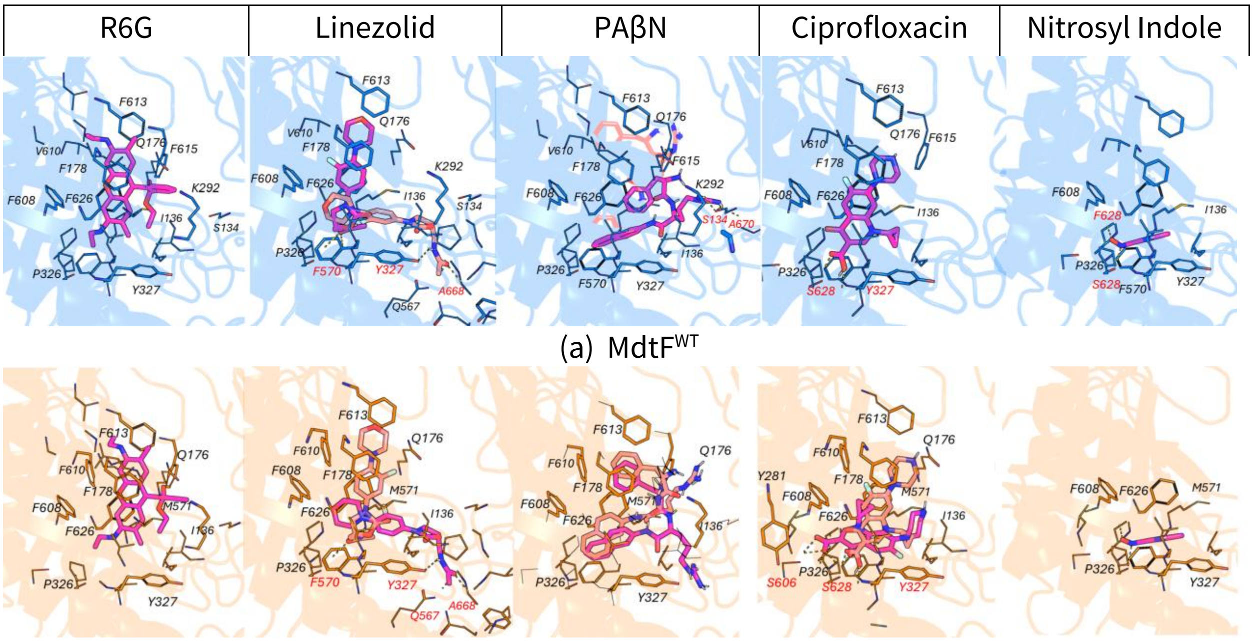

(a) MdtF^{WT}

(b) MdtF^{V610F}

Supplementary Fig. 15 | Docking poses in the DBP of MdtF. Key residues (side chains) are shown in line representation, while ligands are depicted in stick representation. When an alternative pose with comparable binding affinity is identified, the second-best ranked pose is also displayed. The best-ranked ligand pose is coloured dark pink, while the alternative pose is shown in light pink. Hydrogen bonding interactions are represented by dotted lines and labelled in red. The protein is rendered as a transparent cartoon, with MdtF^{WT} and MdtF^{V610F} structures shown in blue and gold, respectively. Panels **(a)** and **(b)** are the complexes for MdtF^{WT} and MdtF^{V610F}, respectively. DBP: Distal binding pocket, PAβN (phenylalanine-arginine β-naphthylamide), R6G: Rhodamine 6G.

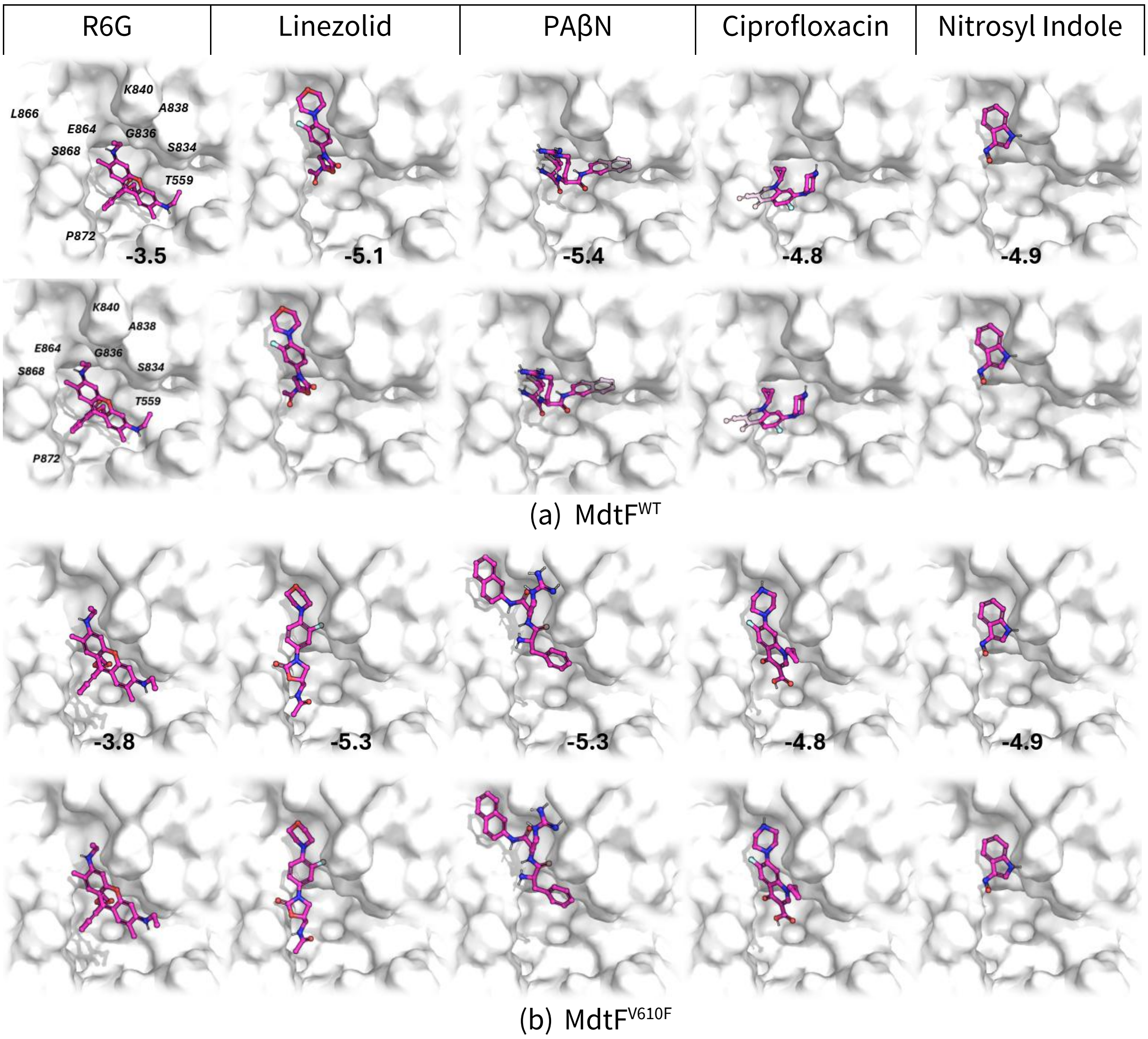

Supplementary Fig. 16 | Docking poses at the CH1 entrance of MdtF^{WT} and MdtF^{V610F}. Protein is represented as white transparent surface for clarity. CH1: Channel 1, PAβN (phenylalanine-arginine β-naphthylamide), R6G: Rhodamine 6G.

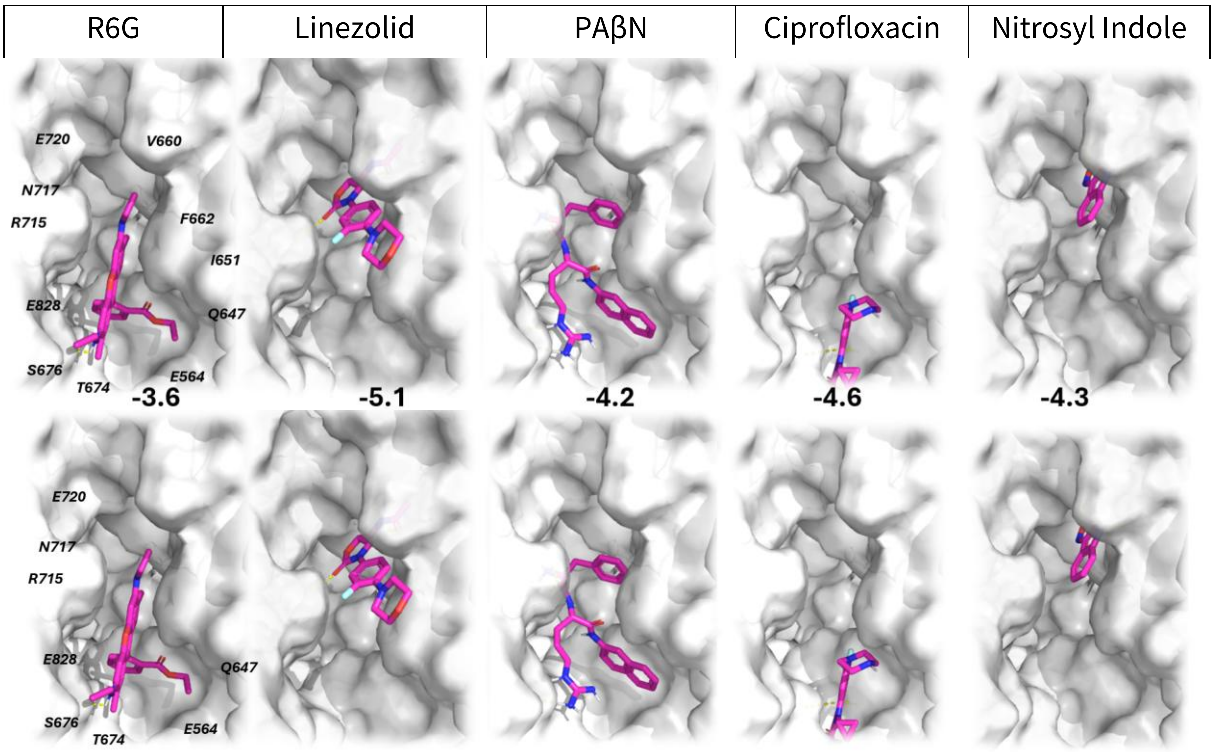

(c) MdtF^{WT}

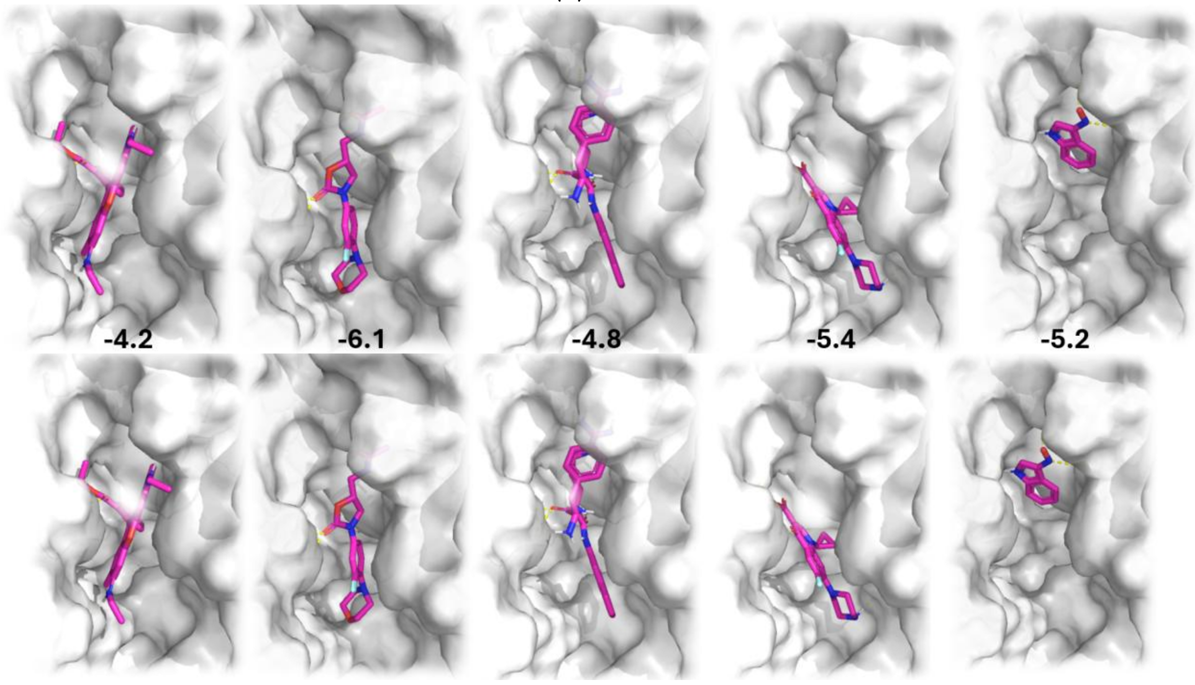

(d) MdtF^{V610F}

Supplementary Fig. 17 | Docking poses at the CH2 entrance of MdtF^{WT} and MdtF^{V610F}. Protein is represented as white transparent surface for clarity. CH2: Channel 2, PAβN (phenylalanine-arginine β-naphthylamide), R6G: Rhodamine 6G.

R6G	Linezolid	PAβN	Ciprofloxacin	Nitrosyl Indole

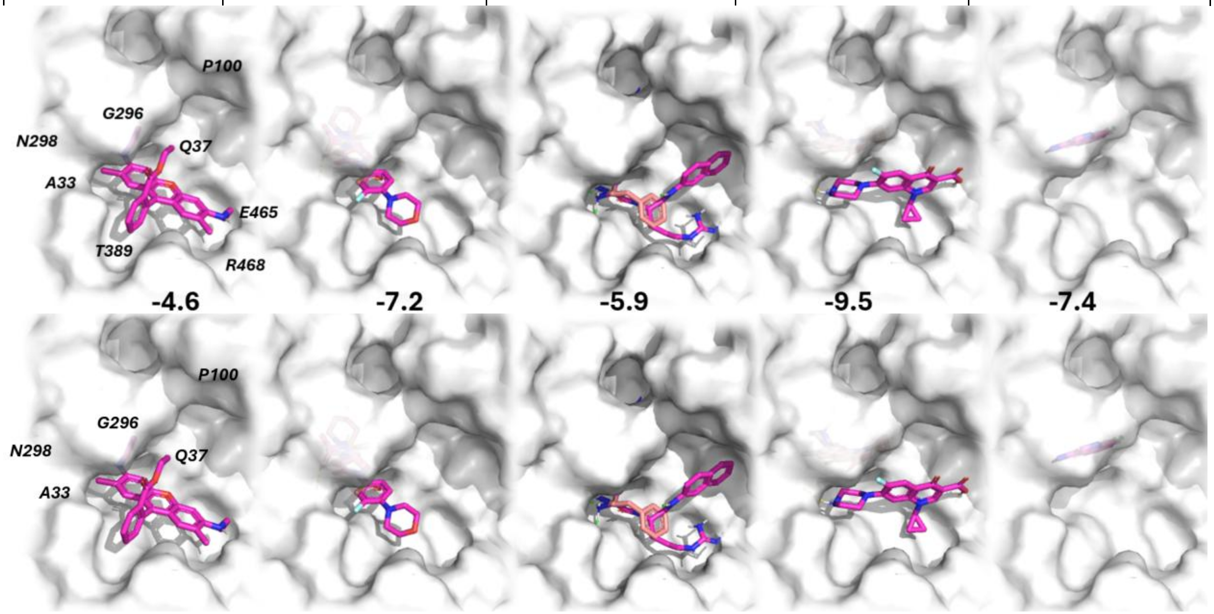

(a) $MdtF^{WT}$

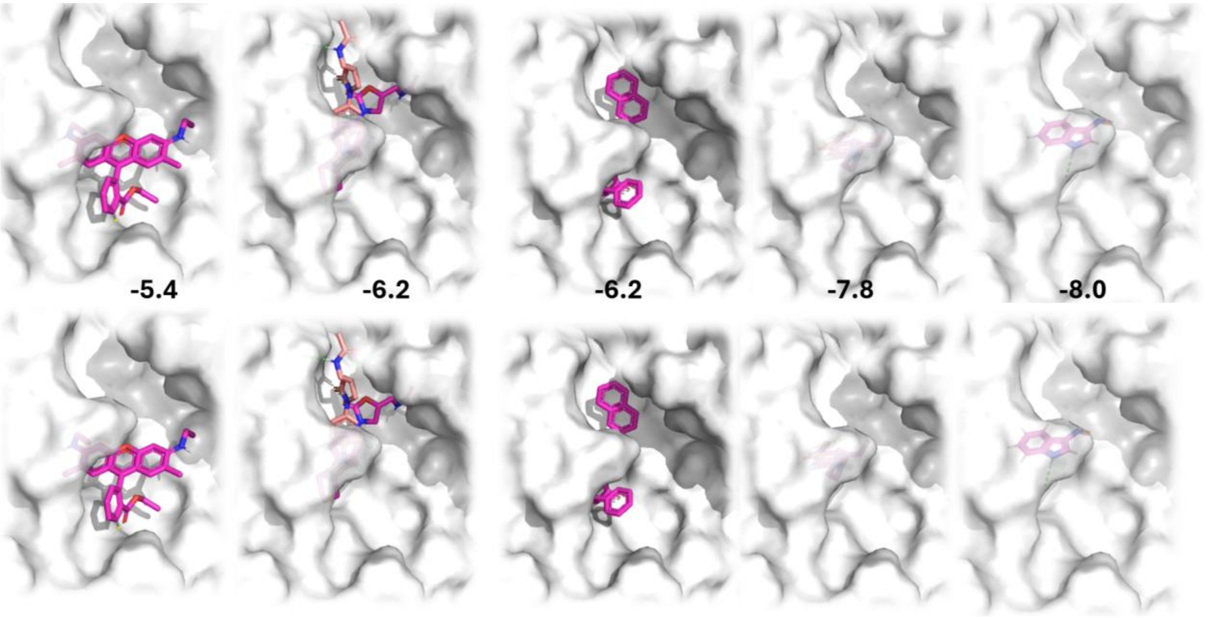

(b) $MdtF^{V610F}$

Supplementary Fig. 18 | Docking poses at the CH3 entrance of $MdtF^{WT}$ and $MdtF^{V610F}$. Protein is represented as white transparent surface for clarity. When an alternative pose with similar binding affinity is found, it is shown in light pink alongside the top-ranked pose in dark pink. CH3: Channel 3, PAβN (phenylalanine-arginine β-naphthylamide), R6G: Rhodamine 6G.

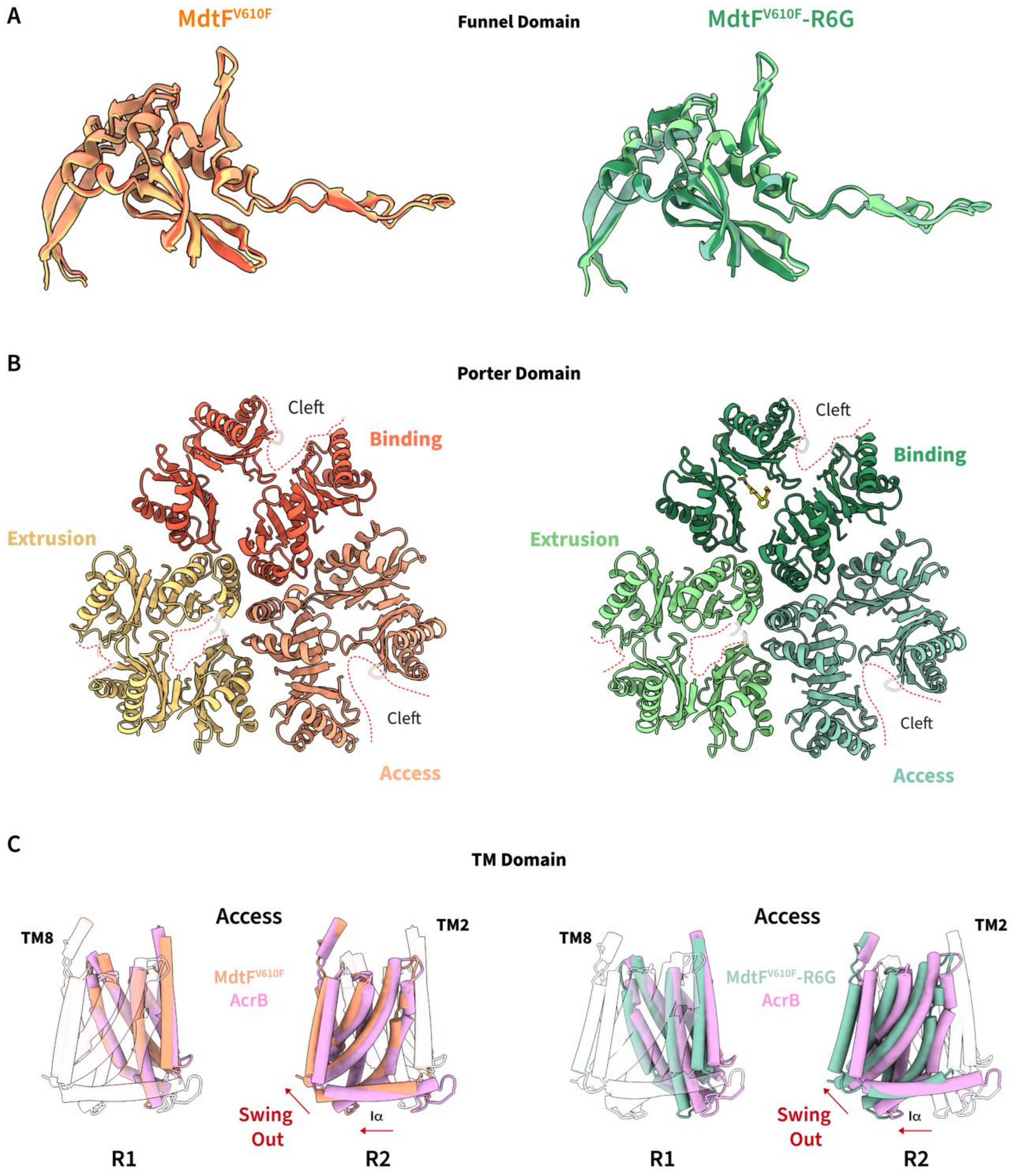

Supplementary Fig. 19 | MdtF^{V610F} and R6G-MdtF^{V610F} structures exhibit MdtF^{WT}-like 'swung out' R2 state. a, Alignment of the funnel domain from each of the three protomeric states of MdtF^{V610F} and R6G-MdtF^{V610F} demonstrate the conserved asymmetry within this domain. **b,** The RND-typical functional asymmetry of the porter domain can be observed within both the MdtF^{V610F} and R6G-MdtF^{V610F} structures. **c,** Alignment of TM domains of both MdtF^{V610F} (orange) and R6G-MdtF^{V610F} (green) with AcrB (pink) indicate that the structural differences between its helical arrangements as it cycles through the protomeric states as observed within MdtF^{WT} (**Fig. 2C**). MdtF^{V610F} and R6G-MdtF^{V610F} structures are demonstrated on the left and right of the figures, respectively. TM: transmembrane.

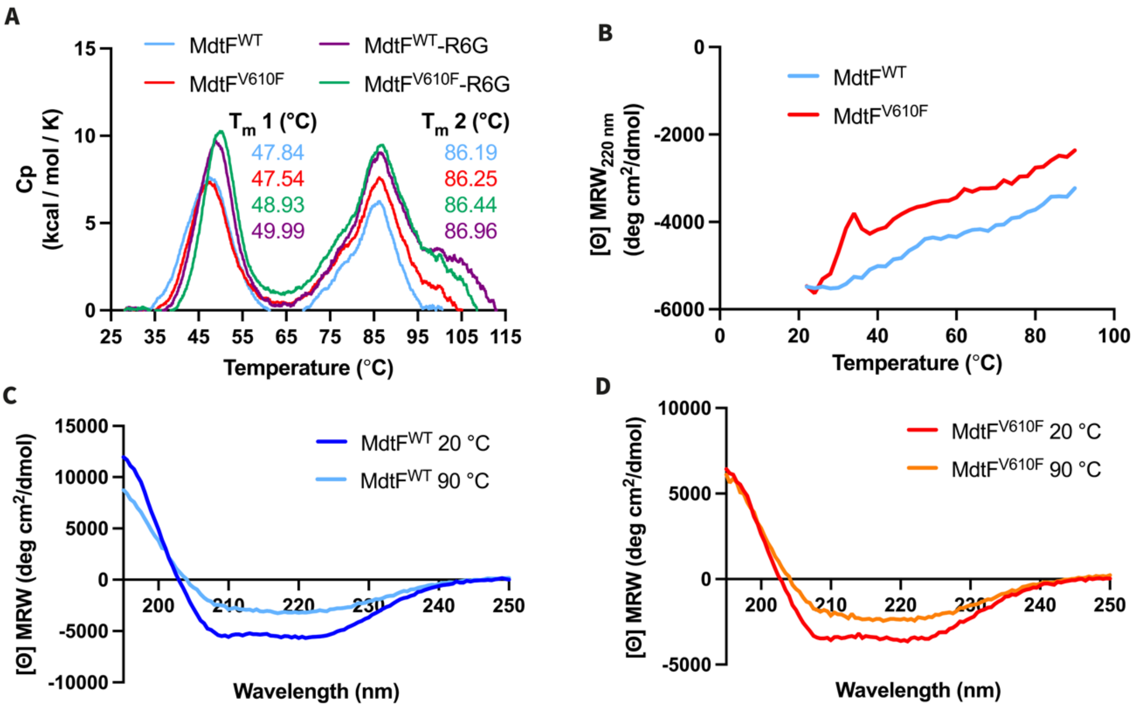

Supplementary Fig. 20 | Monitoring MdtF thermal stability by CD and DSC. a, DSC demonstrated two thermal transitions. The two melting temperatures for each protein (± R6G) are indicated on the graphs. The first peak likely corresponds to the periplasmic domain of the protein and undergoes a stabilising upon R6G binding; however, the second peak likely corresponds to the highly stable transmembrane helices which are maintained within the native nanodiscs and remain unaffected by R6G binding. **b,** To further explore this, we utilised CD experiments to demonstrate the secondary structure melting. Here, CD displayed the periplasmic protein domain transition in accordance with DSC, however, the spectra show the protein does not fully unfold up to 90°C (**c and d**). Again, this suggests that the second transition corresponds to the stable, buried TM a-helical bundle. CD: Circular dichroism, Cp: Specific heat, DSC: Differential scanning calorimetry, MRW: Mean residue ellipticity, R6G: Rhodamine 6G, T_m: Melting temperature, TM: transmembrane helix.

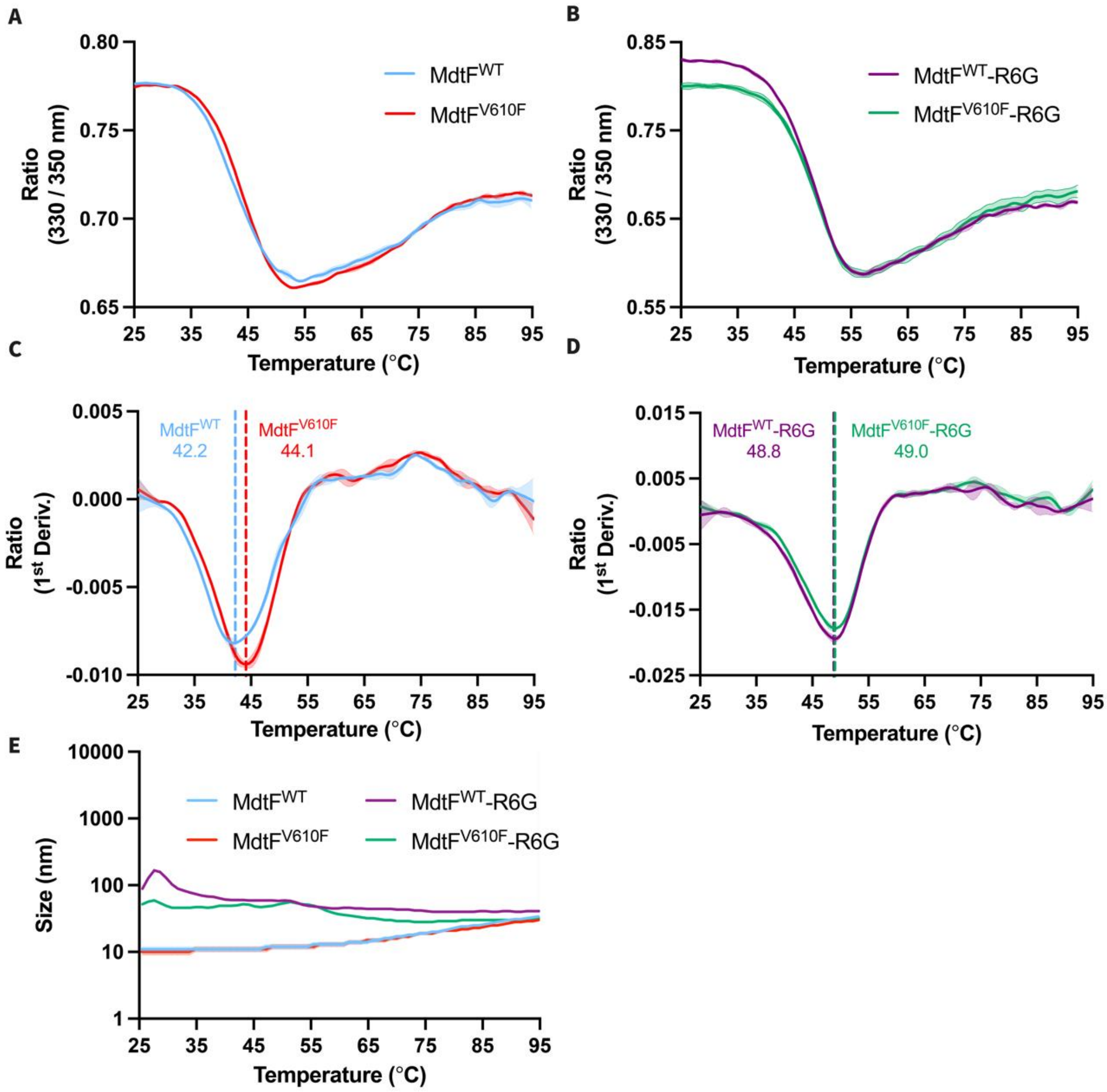

Supplementary Fig. 21 | Monitoring MdtF thermal stability by DSF. a-d, Finally, the stabilising effect of R6G binding to both $MdtF^{WT}$ and $MdtF^{V610F}$ structure of ~5°C was corroborated through monitoring the specific probing of the tertiary environment using DSF. **e,** Here, we also revealed that the self-assembled lipid nanodiscs remain intact during thermal MdtF unfolding which consolidates that this response is not due to possible artefacts arising from particle or oligomer disassembly. Collectively, this supports the thermal stability of MdtF within the SMALP nanodisc in addition to the stabilising effect arising from R6G binding. DSF: differential scanning fluorimetry, SMALP: Styrene maleic acid lipid particle, R6G: Rhodamine 6G.

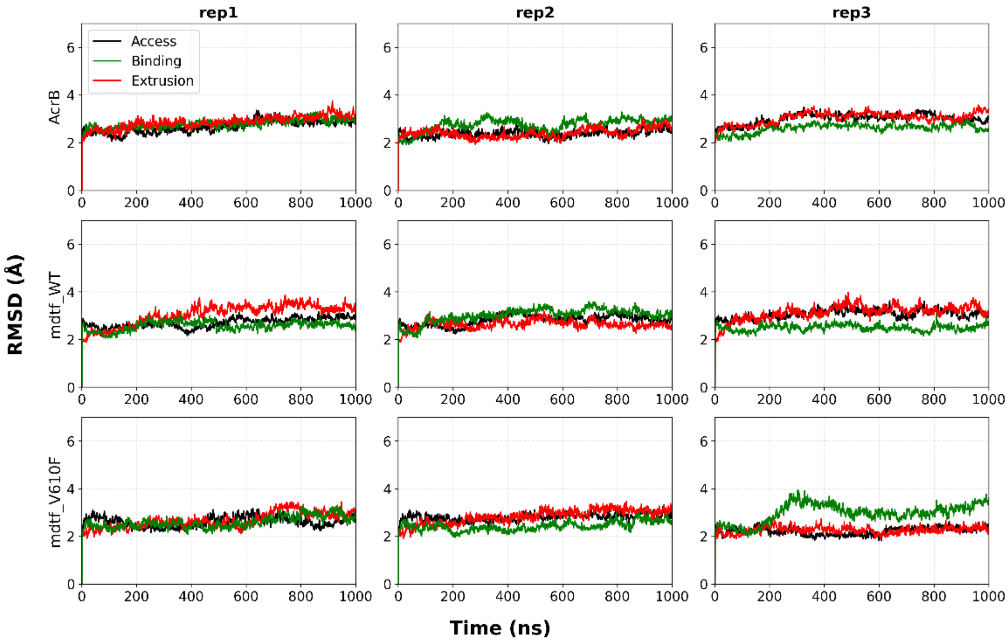

Supplementary Fig. 22 | RMSD fluctuations between MdtF and AcrB. RMSD profiles of Cα atoms computed from the initial experimental structures of AcrB (PDB ID: 4DX5) and MdtF (wild-type and V610F mutant cryo-EM structures reported in this work) across three functional states (Access, Binding, and Extrusion) from three independent 1 ms replicas.

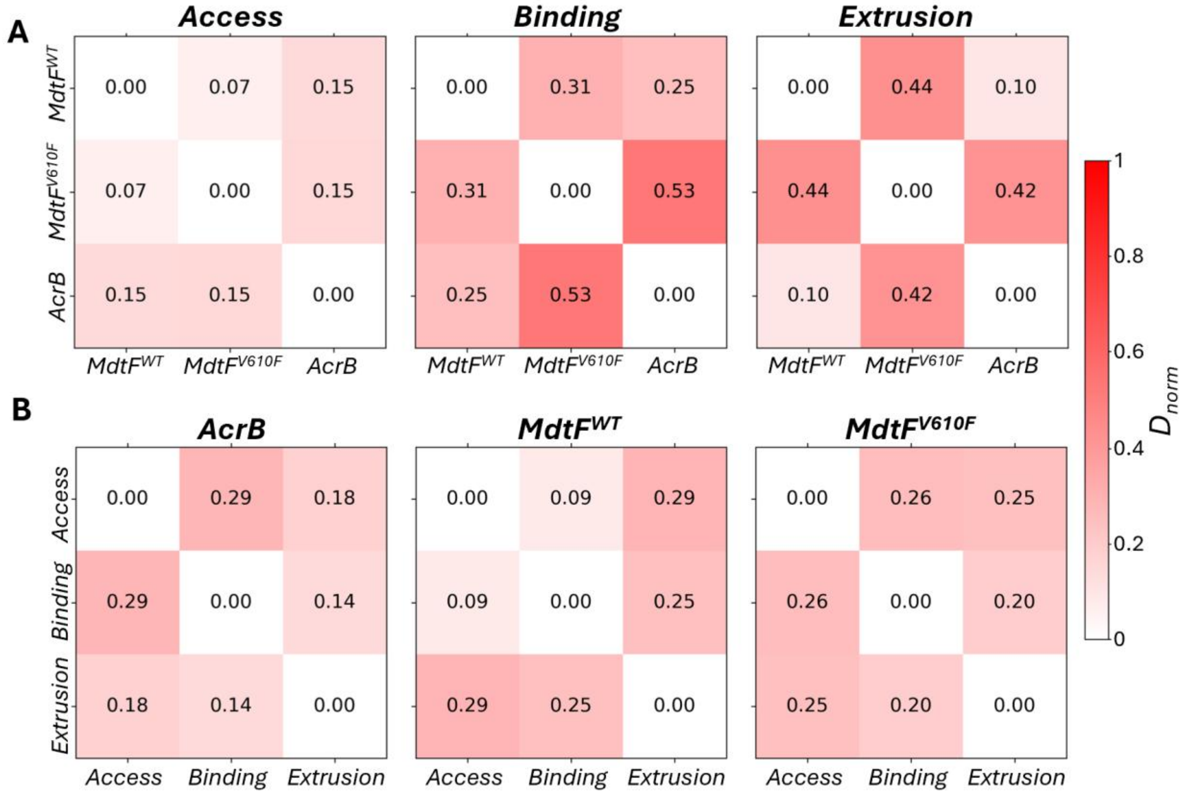

Supplementary Fig. 23 | PC1-PC2 pseudo-contacts distribution analysis between monomers and proteins. The matrices show the Kolmogorov-Smirnov (*Dnorm*) statistic (calculated using ks_2samp from SciPy), which quantifies the maximum difference between the empirical cumulative distribution functions (ECDFs) of PC1-PC2 pseudo-contacts. **(A)** Comparison within access, binding, and extrusion states of different proteins ($MdtF^{WT}$, $MdtF^{V610F}$, and AcrB), and **(B)** comparison within each protein across different functional states (access, binding, extrusion). The KS statistic is calculated as: $D = \max|F_1(x) - F_2(x)|$, where: $F_1(x)$ and $F_2(x)$ are the ECDFs of two monomer or protein dataset. The colour intensity represents the magnitude of normalised D statistics (Dnorm) differences in pseudo-contact distributions, with white (*D = 0*) indicating perfect overlap between the two distributions (similar) and red (high, *D = 1*) indicating no overlap between the two distributions (dissimilar).

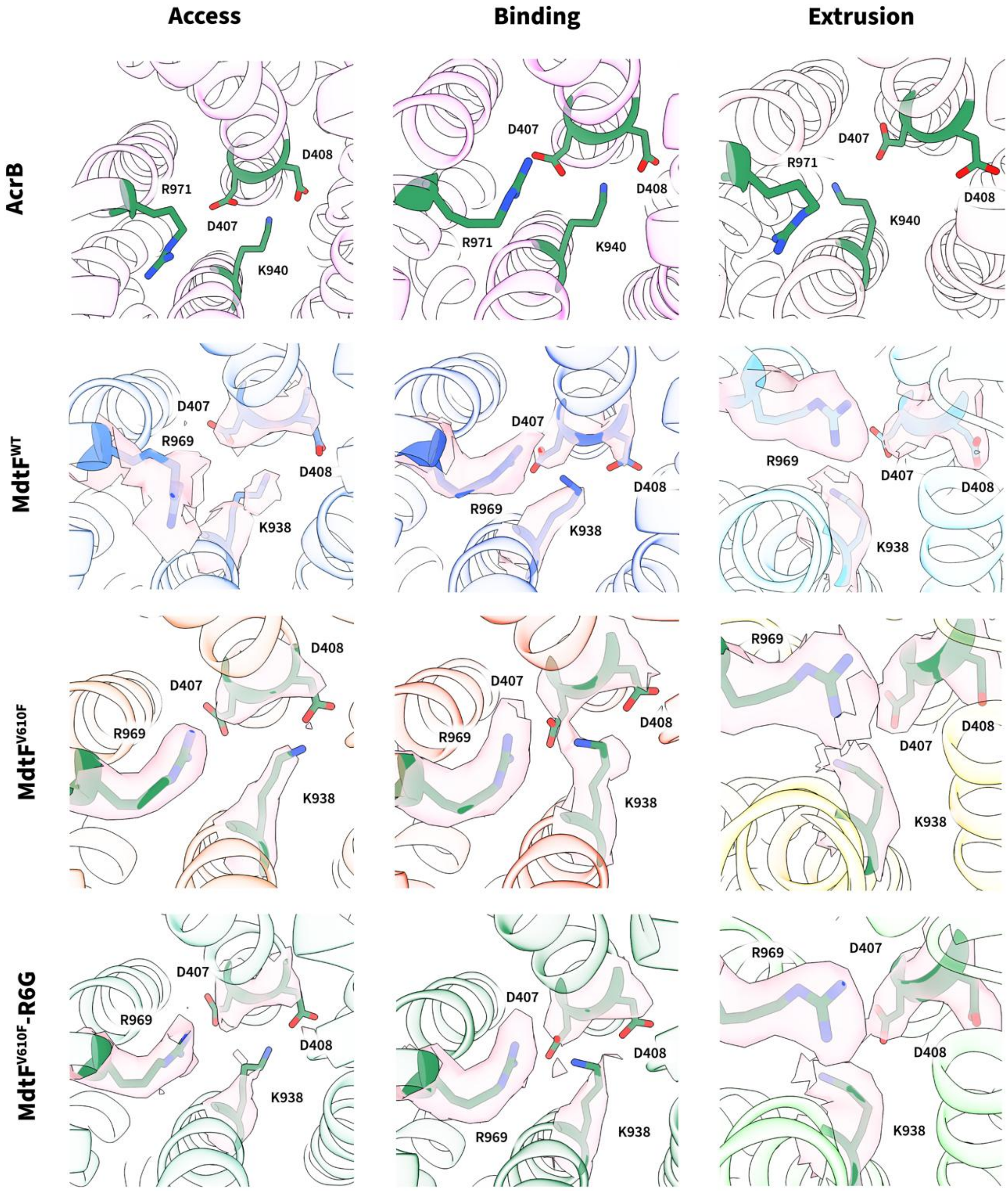

Supplementary Fig. 24 | Conservation of proton relay network residues across MdtF structures. The proton relay residues D407, D408, K938, and R969 across MdtF^{WT}, MdtF^{V610F}, and MdtF^{V610F}-R6G and their respective electron density is shown. The residues and their respective conformational transitions between monomeric states are conserved, including the K938 transition. Density maps are represented in surface form (light pink) and are visualised at a contour level of 0.025. R6G: Rhodamine 6G.

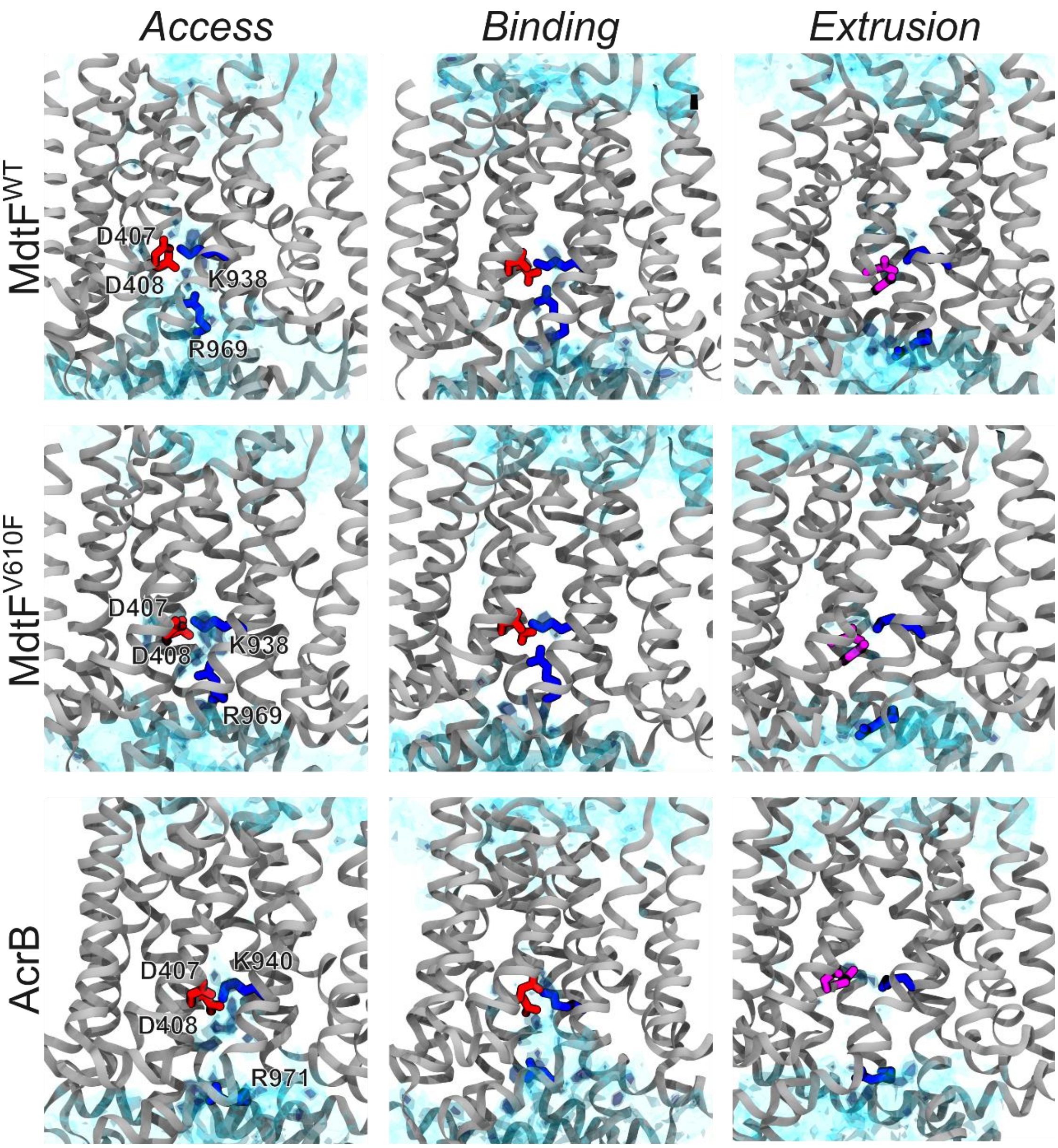

Supplementary Fig. 25 | Hydration at the proton relay site. Figure shows the preferred cumulative location of hydration sites (cyan surfaces) calculated along the MD simulations of the three systems discussed here. Surface transparency reflects water density, with higher density appearing more opaque and lower density more transparent. Residues D407 and D408 are shown in red liquorice for the access and binding states and in pink for the extrusion state, to reflect their different preferred protonation state. K938 (K940) and R969 (R971) are depicted in blue liquorice for MdtF (AcrB). MD: Molecular dynamics.

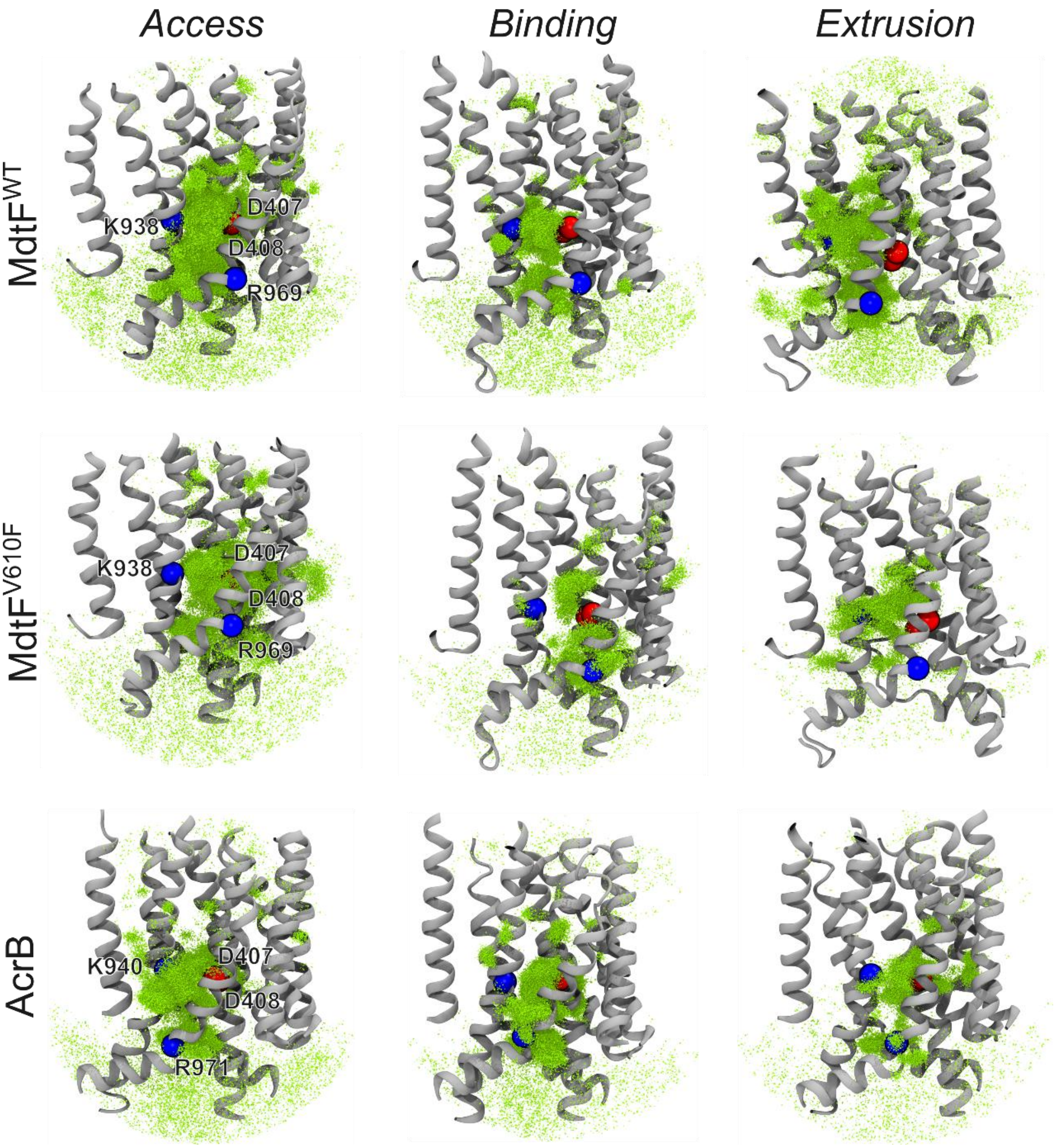

Supplementary Fig. 26 | Water accessibility at the proton relay site. Dynamic water exchange showing the locations where water molecules are exchanged to the proton relay site (within 5 Å) in the transmembrane region. It shows the accessibility of water in AcrB and MdtF^{WT} and MdtF^{V610F}.

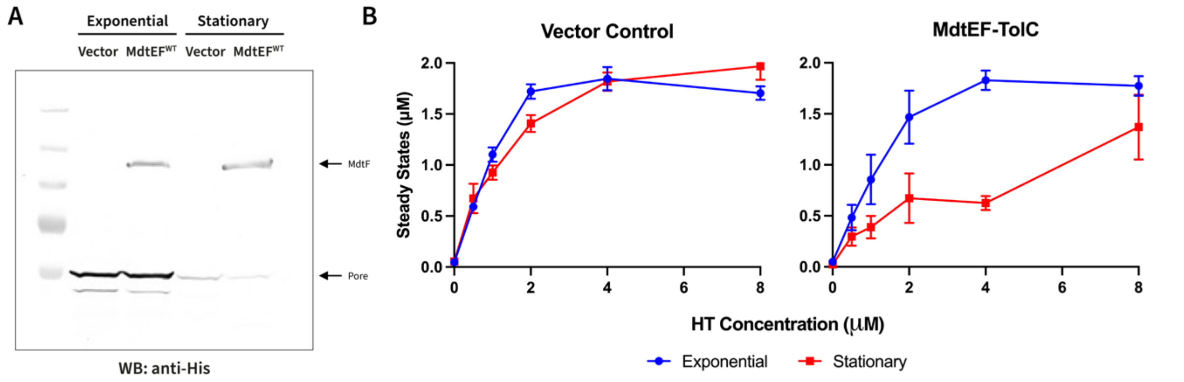

Supplementary Fig. 27 | Outer membrane pore expression differs between exponential and stationary phases. a, MdtEF and Pore expression test through anti-His immunoblotting. Although MdtEF^{WT} expression was observed at both exponential and stationary phases, there was a lower expression of the outer membrane pore observed at stationary phases which is likely a consequence of arabinose metabolism within the *E. coli* Δ9-pore cells. **b,** Steady state accumulation levels of HT were measured in *E. coli* Δ9-pore cells in HMG buffer (50 mM HEPES-KOH, 0.4 % (w/v) glucose, 1 mM $MgSO_4$, pH 7.0), harbouring an empty vector or pUC19-MdtEF^{WT} plasmid, as a function of external HT concentration. Cellular expression of an outer membrane pore was induced with 0.1% L-arabinose. Cells were grown to exponential (4h of growth) and stationary (20 h of growth) phases of growth. Individual data points represent mean values from three independent measurements and error bars are indicative of the standard deviation (n = 3). Due to the differential expression of the outer membrane pore in the stationary phase, there is not convincing evidence that MdtF has a different efficiency in stationary phase non-growing cells. HT: Hoechst 33342, PVDF: Polyvinylidene fluoride, WB: Western blot.

Section 4 References

1 Zwama M, Yamasaki S, Nakashima R, Sakurai K, Nishino K, Yamaguchi A. Multiple entry pathways within the efflux transporter AcrB contribute to multidrug recognition. *Nature Communications 2018 9:1* 2018; **9**: 1–9.

2 Schuster S, Vavra M, Greim L, Kern W V. Exploring the contribution of the AcrB homolog MdtF to drug resistance and dye efflux in a multidrug resistant E. coli isolate. *Antibiotics* 2021; **10**: 503.

3 Zhang Y, Xiao M, Horiyama T, Zhang Y, Li X, Nishino K *et al.* The Multidrug Efflux Pump MdtEF Protects against Nitrosative Damage during the Anaerobic Respiration in Escherichia coli. *Journal of Biological Chemistry* 2011; **286**: 26576–26584.

4 Eicher T, Cha HJ, Seeger MA, Brandstätter L, El-Delik J, Bohnert JA *et al.* Transport of drugs by the multidrug transporter AcrB involves an access and a deep binding pocket that are separated by a switch-loop. *Proc Natl Acad Sci U S A* 2012; **109**: 5687–5692.

5 Qiu W, Fu Z, Xu GG, Grassucci RA, Zhang Y, Frank J *et al.* Structure and activity of lipid bilayer within a membrane-protein transporter. *Proc Natl Acad Sci U S A* 2018; **115**: 12985–12990.

6 Zhang Z, Morgan CE, Cui M, Yu EW. Cryo-EM Structures of AcrD Illuminate a Mechanism for Capturing Aminoglycosides from Its Central Cavity. *mBio* 2023; **14**. doi:10.1128/MBIO.03383-22/SUPPL_FILE/MBIO.03383-22-S0010.PDF.

7 Bharatham N, Bhowmik P, Aoki M, Okada U, Sharma S, Yamashita E *et al.* Structure and function relationship of OqxB efflux pump from Klebsiella pneumoniae. *Nat Commun* 2021; **12**. doi:10.1038/S41467-021-25679-0.

8 Morgan CE, Glaza P, Leus I V., Trinh A, Su CC, Cui M *et al.* Cryoelectron microscopy structures of adeb illuminate mechanisms of simultaneous binding and exporting of substrates. *mBio* 2021; **12**: 1–15.

9 Sakurai K, Yamasaki S, Nakao K, Nishino K, Yamaguchi A, Nakashima R. Crystal structures of multidrug efflux pump MexB bound with high-molecular-mass compounds. *Scientific Reports 2019 9:1* 2019; **9**: 1–9.

10 Long F, Su CC, Zimmermann MT, Boyken SE, Rajashankar KR, Jernigan RL *et al.* Crystal structures of the CusA efflux pump suggest methionine-mediated metal transport. *Nature* 2010; **467**: 484–488.

11 Robert X, Gouet P. Deciphering key features in protein structures with the new ENDscript server. *Nucleic Acids Res* 2014; **42**: W320–W324.

12 Madeira F, Madhusoodanan N, Lee J, Eusebi A, Niewielska A, Tivey ARN *et al.* The EMBL-EBI Job Dispatcher sequence analysis tools framework in 2024. *Nucleic Acids Res* 2024; **52**: W521–W525.

13 Seeger MA, Schiefner A, Eicher T, Verrey F, Diederichs K, Pos KM. Structural asymmetry of AcrB trimer suggests a peristaltic pump mechanism. *Science (1979)* 2006; **313**: 1295–1298.

14 Abramson J, Adler J, Dunger J, Evans R, Green T, Pritzel A *et al.* Accurate structure prediction of biomolecular interactions with AlphaFold 3. *Nature 2024 630:8016* 2024; **630**: 493–500.

15 Luk ́ L, Luká ̌ L, Pravda L, Sehnal D, Toušek D, Toušek T *et al.* MOLEonline: a web-based tool for analyzing channels, tunnels and pores (2018 update). *Nucleic Acids Res* 2018; **46**: W368–W373.
