# Supplementary material for: Molecular basis for multidrug efflux by an anaerobic-associated RND transporter
Source: Nat Commun. 2025 Dec 3;16:10601. doi: 10.1038/s41467-025-65565-7 (PMC12675537; doi:10.1038/s41467-025-65565-7)
Supplement: Supplementary file 2 — Reporting Summary [file 41467_2025_65565_MOESM2_ESM.pdf]

nature portfolio

Corresponding author(s): Eamonn Reading
Zainab Ahdash

Last updated by author(s): Sep 30, 2025

Reporting Summary

Nature Portfolio wishes to improve the reproducibility of the work that we publish. This form provides structure for consistency and transparency in reporting. For further information on Nature Portfolio policies, see our Editorial Policies and the Editorial Policy Checklist.

Statistics

For all statistical analyses, confirm that the following items are present in the figure legend, table legend, main text, or Methods section.

n/a	Confirmed	
☐	☒	The exact sample size (n) for each experimental group/condition, given as a discrete number and unit of measurement
☐	☒	A statement on whether measurements were taken from distinct samples or whether the same sample was measured repeatedly
☒	☐	The statistical test(s) used AND whether they are one- or two-sided<br>*Only common tests should be described solely by name; describe more complex techniques in the Methods section.*
☒	☐	A description of all covariates tested
☐	☒	A description of any assumptions or corrections, such as tests of normality and adjustment for multiple comparisons
☐	☒	A full description of the statistical parameters including central tendency (e.g. means) or other basic estimates (e.g. regression coefficient) AND variation (e.g. standard deviation) or associated estimates of uncertainty (e.g. confidence intervals)
☐	☒	For null hypothesis testing, the test statistic (e.g. F, t, r) with confidence intervals, effect sizes, degrees of freedom and P value noted<br>*Give P values as exact values whenever suitable.*
☒	☐	For Bayesian analysis, information on the choice of priors and Markov chain Monte Carlo settings
☒	☐	For hierarchical and complex designs, identification of the appropriate level for tests and full reporting of outcomes
☒	☐	Estimates of effect sizes (e.g. Cohen's d, Pearson's r), indicating how they were calculated

Our web collection on statistics for biologists contains articles on many of the points above.

Software and code

Policy information about availability of computer code

Data collection	Cryo-EM data collection: EPU software for the Glacios TEM (Thermofisher) equipped with either a Falcon III (Thermofisher) or Falcon 4 (Thermofisher) detector. GC-MS data was collected on a Shimadzu QP2020 NX. LC-MS data was collected on a Vanquish Flex LC coupled to a Q Exactive Plus mass spectrometer.
Data analysis	Relion-4.0 was used for cryo-EM data analysis. Atomic models were built in Coot (version 0.9.8.3). Atomic coordinates were refined using PHENIX (version 1.20). ChimeraX-1.4 was used for visualisation of cryo-EM maps and models and preparation of figures. GC-MS data was analysed using GCMSsolution software (version 4.52, Shimadzu Scientific Instruments, Kyoto, Japan). LC-MS data analysis was conducted using Expressionist software (version 15, Genedata, Basel) SigmaPlot-15.0 and GraphPasd Prism 10 was used for analysis and visualisation of functional assay data.

For manuscripts utilizing custom algorithms or software that are central to the research but not yet described in published literature, software must be made available to editors and reviewers. We strongly encourage code deposition in a community repository (e.g. GitHub). See the Nature Portfolio guidelines for submitting code & software for further information.

Data

Policy information about availability of data

All manuscripts must include a data availability statement. This statement should provide the following information, where applicable:

- Accession codes, unique identifiers, or web links for publicly available datasets
- A description of any restrictions on data availability
- For clinical datasets or third party data, please ensure that the statement adheres to our policy

Cryo-EM density maps and structure coordinates have been deposited in the Electron Microscopy Data Bank (EMDB) and the Protein Data Bank (PDB) with the following accession codes: WT apo-MdtF (EMD-53281 and PDB 9QPR), V610F apo-MdtF (EMD-53282 and PDB 9QPS), R6G-bound V610F MdtF (EMD-53283 and PDB 9QPT). Source data are provided with this paper. MD simulation trajectories and the docking poses at the DBP, CH1, CH2 and CH3 are available at zenodo: 10.5281/zenodo.15038634. GC-MS data is available at the NIH Common Fund's National Metabolomics Data Repository (NMDR) website, the Metabolomics Workbench, https://www.metabolomicsworkbench.org where it has been assigned Project ID (PR002568). The data can be accessed directly via its Project DOI: https://doi.org/10.21228/M8Q54N. The LC-MS data generated in this study have been deposited in the MetaboLights database under accession codes MTBLS13050 (doi: https://www.ebi.ac.uk/metabolights/reviewere5199fba-9710-43ba-8993-49a231a65e2a).

Research involving human participants, their data, or biological material

Policy information about studies with human participants or human data. See also policy information about sex, gender (identity/presentation), and sexual orientation and race, ethnicity and racism.

Reporting on sex and gender	This information has not been collected
Reporting on race, ethnicity, or other socially relevant groupings	See above
Population characteristics	See above
Recruitment	See above
Ethics oversight	See above

Note that full information on the approval of the study protocol must also be provided in the manuscript.

Field-specific reporting

Please select the one below that is the best fit for your research. If you are not sure, read the appropriate sections before making your selection.

☒ Life sciences ☐ Behavioural & social sciences ☐ Ecological, evolutionary & environmental sciences

For a reference copy of the document with all sections, see nature.com/documents/nr-reporting-summary-flat.pdf

Life sciences study design

All studies must disclose on these points even when the disclosure is negative.

Sample size	For cryo-EM data sets: 5,675 movies were collected for WT apo-MdtF and 8,000 movies were collected for both apo and R6G-bound V610F MdtF. For functional assays (including fluorescence polarisation, Nile Red efflux, HT and NPN accumulation, and minimum inhibitory concentration), all measurements were performed in at least triplicates (n=3).
Data exclusions	Data exclusion during cryo-EM data collection, processing, and analysis was performed in accordance with established standards in the field. Micrographs of poor quality were excluded based on crystalline ice contamination, ice thickness, high specimen motion which resulted in poor CTF fit estimates. Cryo-EM particle images were classified using 2D and 3D classification steps in order to remove poor quality particles.
Replication	All functional assays were repeated independently and all attempts at replication were successful with number of replications detailed in text. GC-MS and LC-MS data were performed in triplicate on the same day.
Randomization	The nature of this study does not require randomisation because it does not involve a clinical trial or treatment allocation.
Blinding	Blinding was not relevant to this study. No live subjects were involved and the sample preparations were performed in an experimental laboratory.

Reporting for specific materials, systems and methods

We require information from authors about some types of materials, experimental systems and methods used in many studies. Here, indicate whether each material, system or method listed is relevant to your study. If you are not sure if a list item applies to your research, read the appropriate section before selecting a response.

Materials & experimental systems

n/a	Involved in the study
☒	☐ Antibodies
☒	☐ Eukaryotic cell lines
☒	☐ Palaeontology and archaeology
☒	☐ Animals and other organisms
☒	☐ Clinical data
☒	☐ Dual use research of concern
☒	☐ Plants

Methods

n/a	Involved in the study
☒	☐ ChIP-seq
☒	☐ Flow cytometry
☒	☐ MRI-based neuroimaging

Plants

Seed stocks	No plant materials were used in this study.
Novel plant genotypes	No plant materials were used in this study.
Authentication	No plant materials were used in this study.
