# Supplementary material for: Molecular basis for multidrug efflux by an anaerobic-associated RND transporter
Source: Nat Commun. 2025 Dec 3;16:10601. doi: 10.1038/s41467-025-65565-7 (PMC12675537; doi:10.1038/s41467-025-65565-7)
Supplement: Supplementary file 3 — Description of Additional Supplementary Files [file 41467_2025_65565_MOESM3_ESM.pdf]

Description of Additional Supplementary Files

File name: Supplementary Data 1

File description: Sequences of plasmids and single point mutants used.
