# Supplementary material for: Molecular basis for multidrug efflux by an anaerobic-associated RND transporter
Source: Nat Commun. 2025 Dec 3;16:10601. doi: 10.1038/s41467-025-65565-7 (PMC12675537; doi:10.1038/s41467-025-65565-7)
Supplement: Supplementary file 4 — Supplementary Data 1 [file 41467_2025_65565_MOESM4_ESM.docx]

**Plasmids:**

P151A-AcrAB

ATTTTTTCGCCAACTTCGATTGCGTGATGCTTGATGTCTGCCCTATTCGCATCGGTGATAACTGTATGTTGGCACCAGGCGTTCATATCTACACGGCAACACATCCCATCGACCCTGTAGCACGTAATAGCGGTGCTGAACTGGGGAAACCCGTCACCATCGGTAATAACGTCTGGATTGGCGGACGCGCGGTCATTAACCCTGGTGTGACCATTGGTGATAACGTCGTGGTAGCCTCAGGTGCAGTTGTCACAAAAGATGTCCCGGACAACGTTGTCGTGGGCGGTAATCCAGCCAGAATAATTAAAAAATTGTAATCGGTTTTTCGCAACTGTTATGCAAAATTGTGGTAGATCCTCTAGAGTCGACCTGCAGGCATGCAAGCTTGGCGTAATCATGGTCATAGCTGTTTCCTGTGTGAAATTGTTATCCGCTCACAATTCCACACAACATACGAGCCGGAAGCATAAAGTGTAAAGCCTGGGGTGCCTAATGAGTGAGCTAACTCACATTAATTGCGTTGCGCTCACTGCCCGCTTTCCAGTCGGGAAACCTGTCGTGCCAGCTGCATTAATGAATCGGCCAACGCGCGGGGAGAGGCGGTTTGCGTATTGGGCGCTCTTCCGCTTCCTCGCTCACTGACTCGCTGCGCTCGGTCGTTCGGCTGCGGCGAGCGGTATCAGCTCACTCAAAGGCGGTAATACGGTTATCCACAGAATCAGGGGATAACGCAGGAAAGAACATGTGAGCAAAAGGCCAGCAAAAGGCCAGGAACCGTAAAAAGGCCGCGTTGCTGGCGTTTTTCCATAGGCTCCGCCCCCCTGACGAGCATCACAAAAATCGACGCTCAAGTCAGAGGTGGCGAAACCCGACAGGACTATAAAGATACCAGGCGTTTCCCCCTGGAAGCTCCCTCGTGCGCTCTCCTGTTCCGACCCTGCCGCTTACCGGATACCTGTCCGCCTTTCTCCCTTCGGGAAGCGTGGCGCTTTCTCATAGCTCACGCTGTAGGTATCTCAGTTCGGTGTAGGTCGTTCGCTCCAAGCTGGGCTGTGTGCACGAACCCCCCGTTCAGCCCGACCGCTGCGCCTTATCCGGTAACTATCGTCTTGAGTCCAACCCGGTAAGACACGACTTATCGCCACTGGCAGCAGCCACTGGTAACAGGATTAGCAGAGCGAGGTATGTAGGCGGTGCTACAGAGTTCTTGAAGTGGTGGCCTAACTACGGCTACACTAGAAGAACAGTATTTGGTATCTGCGCTCTGCTGAAGCCAGTTACCTTCGGAAAAAGAGTTGGTAGCTCTTGATCCGGCAAACAAACCACCGCTGGTAGCGGTGGTTTTTTTGTTTGCAAGCAGCAGATTACGCGCAGAAAAAAAGGATCTCAAGAAGATCCTTTGATCTTTTCTACGGGGTCTGACGCTCAGTGGAACGAAAACTCACGTTAAGGGATTTTGGTCATGAGATTATCAAAAAGGATCTTCACCTAGATCCTTTTAAATTAAAAATGAAGTTTTAAATCAATCTAAAGTATATATGAGTAAACTTGGTCTGACAGTTACCAATGCTTAATCAGTGAGGCACCTATCTCAGCGATCTGTCTATTTCGTTCATCCATAGTTGCCTGACTCCCCGTCGTGTAGATAACTACGATACGGGAGGGCTTACCATCTGGCCCCAGTGCTGCAATGATACCGCGAGACCCACGCTCACCGGCTCCAGATTTATCAGCAATAAACCAGCCAGCCGGAAGGGCCGAGCGCAGAAGTGGTCCTGCAACTTTATCCGCCTCCATCCAGTCTATTAATTGTTGCCGGGAAGCTAGAGTAAGTAGTTCGCCAGTTAATAGTTTGCGCAACGTTGTTGCCATTGCTACAGGCATCGTGGTGTCACGCTCGTCGTTTGGTATGGCTTCATTCAGCTCCGGTTCCCAACGATCAAGGCGAGTTACATGATCCCCCATGTTGTGCAAAAAAGCGGTTAGCTCCTTCGGTCCTCCGATCGTTGTCAGAAGTAAGTTGGCCGCAGTGTTATCACTCATGGTTATGGCAGCACTGCATAATTCTCTTACTGTCATGCCATCCGTAAGATGCTTTTCTGTGACTGGTGAGTACTCAACCAAGTCATTCTGAGAATAGTGTATGCGGCGACCGAGTTGCTCTTGCCCGGCGTCAATACGGGATAATACCGCGCCACATAGCAGAACTTTAAAAGTGCTCATCATTGGAAAACGTTCTTCGGGGCGAAAACTCTCAAGGATCTTACCGCTGTTGAGATCCAGTTCGATGTAACCCACTCGTGCACCCAACTGATCTTCAGCATCTTTTACTTTCACCAGCGTTTCTGGGTGAGCAAAAACAGGAAGGCAAAATGCCGCAAAAAAGGGAATAAGGGCGACACGGAAATGTTGAATACTCATACTCTTCCTTTTTCAATATTATTGAAGCATTTATCAGGGTTATTGTCTCATGAGCGGATACATATTTGAATGTATTTAGAAAAATAAACAAATAGGGGTTCCGCGCACATTTCCCCGAAAAGTGCCACCTGACGTCTAAGAAACCATTATTATCATGACATTAACCTATAAAAATAGGCGTATCACGAGGCCCTTTCGTCTCGCGCGTTTCGGTGATGACGGTGAAAACCTCTGACACATGCAGCTCCCGGAGACGGTCACAGCTTGTCTGTAAGCGGATGCCGGGAGCAGACAAGCCCGTCAGGGCGCGTCAGCGGGTGTTGGCGGGTGTCGGGGCTGGCTTAACTATGCGGCATCAGAGCAGATTGTACTGAGAGTGCACCATATGCGGTGTGAAATACCGCACAGATGCGTAAGGAGAAAATACCGCATCAGGCGCCATTCGCCATTCAGGCTGCGCAACTGTTGGGAAGGGCGATCGGTGCGGGCCTCTTCGCTATTACGCCAGCTGGCGAAAGGGGGATGTGCTGCAAGGCGATTAAGTTGGGTAACGCCAGGGTTTTCCCAGTCACGACGTTGTAAAACGACGGCCAGTGAATTCGAGCTCGGTACCCGGGGATCTCACTGAACAAATCCGACTTGTCTTTAAAATGCCAGTAGATTGCACCGCGCGTAACGCCAGCTGCTTTTGCAATCTCGCCCAGCGAGGTGGATGATACCCCCTGCTGTGAGAAAAGACGTAGAGCCACATCGAGGATGTGTTGGCGCGTTTCTTGCGCTTCTTGTTTGGTTTTTCGTGCCATATGTTCGTGAATTTACAGGCGTTAGATTTACATACATTTGTGAATGTATGTACCATAGCACGACGATAATATAAACGCAGCAATGGGTTTATTAACTTTTGACCATTGACCAATTTGAAATCGGACACTCGAGGTTTACATATGAACAAAAACAGAGGGTTTACGCCTCTGGCGGTCGTTCTGATGCTCTCAGGCAGCTTAGCCCTAACAGGATGTGACGACAAACAGGCCCAACAAGGTGGCCAGCAGATGCCCGCCGTTGGCGTAGTAACAGTCAAAACTGAACCTCTGCAGATCACAACCGAGCTTCCGGGTCGCACCAGTGCCTACCGGATCGCAGAAGTTCGTCCTCAAGTTAGCGGGATTATCCTGAAGCGTAATTTCAAAGAAGGTAGCGACATCGAAGCAGGTGTCTCTCTCTATCAGATTGATCCTGCGACCTATCAGGCGACATACGACAGTGCGAAAGGTGATCTGGCGAAAGCCCAGGCTGCAGCCAATATCGCGCAATTGACGGTGAATCGTTATCAGAAACTGCTCGGTACTCAGTACATCAGTAAGCAAGAGTACGATCAGGCTCTGGCTGATGCGCAACAGGCGAATGCTGCGGTAACTGCGGCGAAAGCTGCCGTTGAAACTGCGCGGATCAATCTGGCTTACACCAAAGTCACCTCTCCGATTAGCGGTCGCATTGGTAAGTCGAACGTGACGGAAGGCGCATTGGTACAGAACGGTCAGGCGACTGCGCTGGCAACCGTGCAGCAACTTGATCCGATCTACGTTGATGTGACCCAGTCCAGCAACGACTTCCTGCGCCTGAAACAGGAACTGGCGAATGGCACGCTGAAACAAGAGAACGGCAAAGCCAAAGTGTCACTGATCACCAGTGACGGCATTAAGTTCCCGCAGGACGGTACGCTGGAATTCTCTGACGTTACCGTTGATCAGACCACTGGGTCTATCACCCTACGCGCTATCTTCCCGAACCCGGATCACACTCTGCTGCCGGGTATGTTCGTGCGCGCACGTCTGGAAGAAGGGCTTAATCCAAACGCTATTTTAGTCCCGCAACAGGGCGTAACCCGTACGCCGCGTGGCGATGCCACCGTACTGGTAGTTGGCGCGGATGACAAAGTGGAAACCCGTCCGATCGTTGCAAGCCAGGCTATTGGCGATAAGTGGCTGGTGACAGAAGGTCTGAAAGCAGGCGATCGCGTAGTAATAAGTGGGCTGCAGAAAGTGCGTCCTGGTGTCCAGGTAAAAGCACAAGAAGTTACCGCTGATAATAACCAGCAAGCCGCAAGCGGTGCTCAGCCTGAACAGTCCAAGTCTTAACTTAAACAGGAGCCGTTAAGACATGCCTAATTTCTTTATCGATCGCCCGATTTTTGCGTGGGTGATCGCCATTATCATCATGTTGGCAGGGGGGCTGGCGATCCTCAAACTGCCGGTGGCGCAATATCCTACGATTGCACCGCCGGCAGTAACGATCTCCGCCTCCTACCCCGGCGCTGATGCGAAAACAGTGCAGGACACGGTGACACAGGTTATCGAACAGAATATGAACGGTATCGATAACCTGATGTACATGTCCTCTAACAGTGACTCCACGGGTACCGTGCAGATCACCCTGACCTTTGAGTCTGGTACTGATGCGGATATCGCGCAGGTTCAGGTGCAGAACAAACTGCAGCTGGCGATGCCGTTGCTGCCGCAAGAAGTTCAGCAGCAAGGGGTGAGCGTTGAGAAATCATCCAGCAGCTTCCTGATGGTTGTCGGCGTTATCAACACCGATGGCACCATGACGCAGGAGGATATCTCCGACTACGTGGCGGCGAATATGAAAGATGCCATCAGCCGTACGTCGGGCGTGGGTGATGTTCAGTTGTTCGGTTCACAGTACGCGATGCGTATCTGGATGAACCCGAATGAGCTGAACAAATTCCAGCTAACGCCGGTTGATGTCATTACCGCCATCAAAGCGCAGAACGCCCAGGTTGCGGCGGGTCAGCTCGGTGGTACGCCGCCGGTGAAAGGCCAACAGCTTAACGCCTCTATTATTGCTCAGACGCGTCTGACCTCTACTGAAGAGTTCGGCAAAATCCTGCTGAAAGTGAATCAGGATGGTTCCCGCGTGCTGCTGCGTGACGTCGCGAAGATTGAGCTGGGTGGTGAGAACTACGACATCATCGCAGAGTTTAACGGCCAACCGGCTTCCGGTCTGGGGATCAAGCTGGCGACCGGTGCAAACGCGCTGGATACCGCTGCGGCAATCCGTGCTGAACTGGCGAAGATGGAACCGTTCTTCCCGTCGGGTCTGAAAATTGTTTACCCATACGACACCACGCCGTTCGTGAAAATCTCTATTCACGAAGTGGTTAAAACGCTGGTCGAAGCGATCATCCTCGTGTTCCTGGTTATGTATCTGTTCCTGCAGAACTTCCGCGCGACGTTGATTCCGACCATTGCCGTACCGGTGGTATTGCTCGGGACCTTTGCCGTCCTTGCCGCCTTTGGCTTCTCGATAAACACGCTAACAATGTTCGGGATGGTGCTCGCCATCGGCCTGTTGGTGGATGACGCCATCGTTGTGGTAGAAAACGTTGAGCGTGTTATGGCGGAAGAAGGTTTGCCGCCAAAAGAAGCTACCCGTAAGTCGATGGGGCAGATTCAGGGCGCTCTGGTCGGTATCGCGATGGTACTGTCGGCGGTATTCGTACCGATGGCCTTCTTTGGCGGTTCTACTGGTGCTATCTATCGTCAGTTCTCTATTACCATTGTTTCAGCAATGGCGCTGTCGGTACTGGTGGCGTTGATCCTGACTCCAGCTCTTTGTGCCACCATGCTGAAACCGATTGCCAAAGGCGATCACGGGGAAGGTAAAAAAGGCTTCTTCGGCTGGTTTAACCGCATGTTCGAGAAGAGCACGCACCACTACACCGACAGCGTAGGCGGTATTCTGCGCAGTACGGGGCGTTACCTGGTGCTGTATCTGATCATCGTGGTCGGCATGGCCTATCTGTTCGTGCGTCTGCCAAGCTCCTTCTTGCCAGATGAGGACCAGGGCGTGTTTATGACCATGGTTCAGCTGCCAGCAGGTGCAACGCAGGAACGTACACAGAAAGTGCTCAATGAGGTAACGCATTACTATCTGACCAAAGAAAAGAACAACGTTGAGTCGGTGTTCGCCGTTAACGGCTTCGGCTTTGCGGGACGTGGTCAGAATACCGGTATTGCGTTCGTTTCCTTGAAGGACTGGGCCGATCGTCCGGGCGAAGAAAACAAAGTTGAAGCGATTACCATGCGTGCAACACGCGCTTTCTCGCAAATCAAAGATGCGATGGTTTTCGCCTTTAACCTGCCCGCAATCGTGGAACTGGGTACTGCAACCGGCTTTGACTTTGAGCTGATTGACCAGGCTGGCCTTGGTCACGAAAAACTGACTCAGGCGCGTAACCAGTTGCTTGCAGAAGCAGCGAAGCACCCTGATATGTTGACCAGCGTACGTCCAAACGGTCTGGAAGATACCCCGCAGTTTAAGATTGATATCGACCAGGAAAAAGCGCAGGCGCTGGGTGTTTCTATCAACGACATTAACACCACTCTGGGCGCTGCATGGGGCGGCAGCTATGTGAACGACTTTATCGACCGCGGTCGTGTGAAGAAAGTTTATGTCATGTCAGAAGCGAAATACCGTATGCTGCCGGATGATATCGGCGACTGGTATGTTCGTGCTGCTGATGGTCAGATGGTGCCATTCTCGGCGTTCTCCTCTTCTCGTTGGGAGTACGGTTCGCCGCGTCTGGAACGTTACAACGGCCTGCCATCCATGGAAATCTTAGGCCAGGCGGCACCGGGTAAAAGTACCGGTGAAGCAATGGAGCTGATGGAACAACTGGCGAGCAAACTGCCTACCGGTGTTGGCTATGACTGGACGGGGATGTCCTATCAGGAACGTCTCTCCGGCAACCAGGCACCTTCACTGTACGCGATTTCGTTGATTGTCGTGTTCCTGTGTCTGGCGGCGCTGTACGAGAGCTGGTCGATTCCGTTCTCCGTTATGCTGGTCGTTCCGCTGGGGGTTATCGGTGCGTTGCTGGCTGCCACCTTCCGTGGCCTGACCAATGACGTTTACTTCCAGGTAGGCCTGCTCACAACCATTGGGTTGTCGGCGAAGAACGCGATCCTTATCGTCGAATTCGCCAAAGACTTGATGGATAAAGAAGGTAAAGGTCTGATTGAAGCGACGCTTGATGCGGTGCGGATGCGTTTACGTCCGATCCTGATGACCTCGCTGGCGTTTATCCTCGGCGTTATGCCGCTGGTTATCAGTACTGGTGCTGGTTCCGGCGCGCAGAACGCAGTAGGTACCGGTGTAATGGGCGGGATGGTGACCGCAACGGTACTGGCAATCTTCTTCGTTCCGGTATTCTTTGTGGTGGTTCGCCGCCGCTTTAGCCGCAAGAATGAAGATATCGAGCACAGCCATACTGTCGATCATCATCATCATCATCATTGAATGCATAACCTACGAACATTAAGGAGTAATTGAACCACCAACTCAGGATCTCATACGAAAACCAGTATTAACCACGGATAAAATTCATAAAAAATACTGATTGTTAGTTAATTTATATTAAGTAGCGCTAATAGATTTAATAATCCATAATCATTTAGAGGCTATTCTTAATTATTTGCGGTAATTCTTTATTCATTCCTCGGTTATTACGTCATATTCAGAGCAATCCTGGTATTAGTGTCACCAATTTCATCTGGCGATAATCCTGAAATGTTATGAATAGTTCGAGCAAACTGCTTTTACCTGCTGCGGGTTAGTGCTAGTATGAAAAAGTGAGTCCTGTCCCGCTTCCTTCCTAATTGTAATTTTTCGTAATAATGCGATGAAAACCTGCAAAGAGTGGCTTATAGTTAAGCTAACAAACGAGAGGGCAAGTCCAGGTCAGTAAGTTTTTTCCATCCCGAAAGGTGTCCGTTAGTTCAACCGCTAAGAAGGGGACGCGTTATGGATGAATACTCACCCAAAAGACATGATATCGCACAGCTTAAGTTTCTCTGTGAAACCCTGTATCATGACTGCCTTGCAAACCTTGAAGAAAGCAATCATGGCTGGGTAAACGACCCAACCTCGGCGATCAACCTCCAGTTGAATGAACTGATTGAGCATATTGCGACCTTCGCACTTAATTACAAAATTAAGTATAATGAAGACAATAAGCTCATTGAGCAGATCGACGAATATCTGGATGACACCTTTATGTTGTTCAGTAGTTATGGTATTAATATGCAGGATCTTCAGAAATGGCGGAAGTCAGGTAATCGACTATTCCGTTGTTTTGTCAATGCGACGAAAGAGAATCCTGCGAGTTTATCTTGTTAGAATTATTACAACCATAGGTAGAAGTATGTCCGAAAAACCTTTAACGAAAACCGATTATTTAATGCGTTTACGTCGTTGCCAGACAATTGACACGCTGGAGCGTGTTATCGAGAAAAATAAATACGAATTATCAGATAATGAACTGGCGGTATTTTACTCAGCCGCAGATCACCGCCTCGCCGAATTGACCATGAATAAACTGTACGACAAGATCCCTTCCTCAGTATGGAAATTTATTCGCTAATAAATAATTCGCTTTCGGAGCTATAACCGGCTGTTTATTAAGAATTTTATACTTTTTCGCCATGAAGACATACCCTATGTGATCTTTATCACACAGATGTAATGGGAACGTTCTCTTCACTGACTTTTCGTCTTACTGTGTTGCCGCATTTTCAGCAACCGGAGGTCAGTAATGAGCACAGAAAAAGAAAAGATGATTGCTGGTGAGTTGTATCGCTCGGCAGATGAGACGTTATCTCGCGATCGCCTGCGCGCTCGTCAGCTTATTCACCGATACAATCATTCCCTGGCGGAAGAGCACACATTACGCCAGCAAATTCTCGCTGATCTATTCGGTCAGGTGACAGAGGCTTATATTGAGCCAACGTTTCGCTGTGACTATGGCTATAACATTTTTCTCGGTAATA

pUC19-MdtEF

AACTCTGTAGCACCGCCTACATACCTCGCTCTGCTAATCCTGTTACCAGTGGCTGCTGCCAGTGGCGATAAGTCGTGTCTTACCGGGTTGGACTCAAGACGATAGTTACCGGATAAGGCGCAGCGGTCGGGCTGAACGGGGGGTTCGTGCACACAGCCCAGCTTGGAGCGAACGACCTACACCGAACTGAGATACCTACAGCGTGAGCTATGAGAAAGCGCCACGCTTCCCGAAGGGAGAAAGGCGGACAGGTATCCGGTAAGCGGCAGGGTCGGAACAGGAGAGCGCACGAGGGAGCTTCCAGGGGGAAACGCCTGGTATCTTTATAGTCCTGTCGGGTTTCGCCACCTCTGACTTGAGCGTCGATTTTTGTGATGCTCGTCAGGGGGGCGGAGCCTATGGAAAAACGCCAGCAACGCGGCCTTTTTACGGTTCCTGGCCTTTTGCTGGCCTTTTGCTCACATGTTCTTTCCTGCGTTATCCCCTGATTCTGTGGATAACCGTATTACCGCCTTTGAGTGAGCTGATACCGCTCGCCGCAGCCGAACGACCGAGCGCAGCGAGTCAGTGAGCGAGGAAGCGGAAGAGCGCCCAATACGCAAACCGCCTCTCCCCGCGCGTTGGCCGATTCATTAATGCAGCTGGCACGACAGGTTTCCCGACTGGAAAGCGGGCAGTGAGCGCAACGCAATTAATGTGAGTTAGCTCACTCATTAGGCACCCCAGGCTTTACACTTTATGCTTCCGGCTCGTATGTTGTGTGGAATTGTGAGCGGATAACAATTTCACACAGGAAACAGCTATGACCATGATTACGCCAAGCTTGCATGCCTGCAGAACTGTTGGAGAACGGCAACATTTTTTTTGTCGTTGACCTCACCATGTCGATCACTGTGCCTGTATCCCACCTTACTGGCTGACAACCCCACTATGCCGCTGGTCTGTAAATCCCTCATATCTCTCCTCGCGCGCAATTTAAAGAACCGTTATTTCTCAAGAATTTTCAGGGACTAAAATGAACAGAAGAAGAAAGCTGTTAATACCGTTGTTATTCTGCGGCGCGATGCTCACCGCCTGCGATGACAAATCGGCGGAAAACGCCGCCGCCATGACGCCTGAGGTCGGTGTCGTCACACTCTCCCCCGGTTCGGTCAATGTGTTGAGCGAATTGCCCGGTAGAACCGTTCCTTATGAAGTTGCCGAGATACGTCCCCAGGTGGGCGGTATTATCATTAAACGCAACTTTATCGAAGGCGATAAAGTGAACCAGGGCGATTCGCTGTATCAGATTGATCCTGCACCTTTACAGGCCGAGCTAAACTCCGCCAAAGGCTCGCTGGCGAAAGCGCTCTCTACCGCCAGCAATGCCCGCATCACCTTTAACCGCCAGGCATCGTTGCTGAAGACCAACTACGTTAGCCGTCAGGATTACGACACCGCGCGCACCCAGTTGAATGAAGCAGAAGCCAATGTCACCGTCGCCAAAGCGGCTGTTGAACAGGCGACGATCAATCTGCAATACGCGAATGTCACCTCGCCGATTACGGGCGTCAGCGGGAAATCGTCGGTGACCGTCGGCGCACTCGTTACCGCTAATCAGGCAGATTCGCTGGTTACCGTACAACGTCTGGACCCGATTTATGTCGATCTCACGCAGTCGGTGCAAGATTTCTTACGCATGAAAGAAGAGGTCGCCAGTGGGCAAATCAAACAGGTTCAGGGCAGTACGCCAGTACAGCTCAATCTGGAAAATGGTAAACGCTACAGCCAGACCGGCACGCTGAAATTCTCCGACCCGACAGTGGATGAAACCACGGGCTCCGTGACGTTACGGGCGATTTTCCCCAACCCAAATGGTGACTTGCTGCCTGGCATGTACGTCACGGCATTAGTGGATGAAGGTAGCCGCCAGAATGTATTACTGGTGCCGCAGGAAGGCGTCACCCACAACGCCCAGGGTAAAGCAACGGCGCTCATTCTGGATAAAGACGATGTCGTGCAGCTACGCGAAATTGAAGCCAGCAAAGCCATCGGCGACCAGTGGGTCGTCACCTCTGGCTTGCAGGCTGGCGATCGGGTGATCGTTTCCGGTTTGCAACGCATTCGTCCGGGTATCAAAGCACGAGCAATTTCCTCCAGCCAGGAAAACGCCAGCACCGAATCGAAACAATAACGTTGCAGGCTTAAGGGGACTTTCATGGCTAACTATTTTATTGATCGCCCGGTTTTTGCCTGGGTACTTGCCATTATTATGATGCTTGCAGGTGGTCTGGCGATCATGAACTTACCGGTTGCGCAGTATCCGCAGATTGCGCCACCGACCATTACCGTCAGCGCTACCTATCCAGGTGCCGATGCGCAAACGGTAGAAGACTCGGTCACTCAGGTGATTGAGCAAAATATGAATGGGCTTGATGGCCTGATGTACATGTCTTCAACCAGTGATGCGGCGGGCAATGCCTCTATCACTCTGACCTTCGAGACTGGGACATCTCCTGATATCGCACAGGTTCAAGTGCAAAATAAACTGCAACTCGCTATGCCTTCATTACCTGAAGCAGTGCAGCAGCAGGGGATTAGCGTCGATAAGTCGAGCAGTAATATCCTGATGGTAGCGGCGTTTATTTCTGATAACGGCAGCCTCAACCAGTACGATATCGCGGACTATGTAGCGTCTAATATCAAAGACCCGCTAAGCCGTACCGCGGGCGTTGGTAGCGTACAACTCTTTGGTTCCGAGTATGCCATGCGTATCTGGCTGGACCCGCAAAAACTCAATAAATATAACCTGGTACCTTCCGATGTTATTTCCCAGATTAAGGTGCAAAACAACCAGATTTCCGGTGGTCAACTGGGTGGCATGCCACAGGCGGCAGACCAGCAGCTAAACGCCTCGATCATTGTGCAGACGCGTCTGCAAACGCCGGAAGAATTTGGCAAAATCCTGTTGAAAGTTCAGCAAGATGGTTCGCAAGTGCTGCTGCGTGATGTCGCTCGCGTCGAACTTGGGGCGGAAGATTATTCCACCGTGGCACGCTATAACGGCAAACCTGCTGCCGGGATCGCCATCAAACTGGCTGCCGGAGCAAACGCCCTGGATACCTCGCGGGCAGTCAAAGAGGAACTGAACCGCTTATCAGCCTATTTCCCGGCAAGTCTGAAGACGGTTTATCCTTACGACACCACGCCGTTTATCGAAATTTCTATTCAGGAAGTTTTCAAAACACTGGTTGAGGCTATCATCCTAGTCTTCCTGGTCATGTATCTGTTTTTGCAGAATTTCCGTGCCACAATCATCCCGACGATTGCCGTACCGGTGGTTATTCTCGGGACGTTTGCGATCTTGTCGGCGGTCGGTTTCACCATCAACACGTTGACTATGTTCGGGATGGTGCTGGCGATAGGGTTACTGGTGGATGACGCCATCGTGGTGGTGGAGAACGTCGAGCGTGTCATTGCGGAAGATAAGCTACCGCCGAAGGAAGCGACGCATAAATCGATGGGGCAGATCCAACGTGCGCTGGTCGGTATTGCCGTTGTTCTTTCCGCAGTGTTTATGCCGATGGCCTTTATGAGCGGTGCAACCGGGGAGATCTACCGCCAGTTCTCCATCACGCTGATCTCCTCCATGCTGCTTTCAGTATTTGTGGCAATGAGCCTGACCCCTGCCCTGTGCGCCACCATTCTGAAAGCCGCGCCGGAAGGCGGTCACAAACCTAACGCCCTGTTCGCACGCTTCAACACGCTGTTTGAAAAATCAACTCAACACTATACCGATAGCACCCGCTCGCTGTTGCGTTGTACCGGTCGCTACATGGTGGTCTACCTGCTGATTTGCGCCGGGATGGCGGTGCTGTTCCTGCGCACGCCGACCTCTTTCTTACCAGAAGAGGATCAGGGGGTATTTATGACCACCGCGCAGTTACCTTCCGGTGCCACCATGGTTAACACCACGAAAGTGCTGCAACAGGTGACGGATTATTATCTGACTAAAGAGAAAGATAATGTCCAGTCGGTGTTTACCGTTGGCGGCTTTGGCTTCAGCGGTCAGGGGCAAAACAACGGCCTGGCGTTTATCAGTCTCAAGCCGTGGTCTGAACGTGTCGGTGAGGAAAACTCGGTTACCGCGATCATTCAGCGGGCAATGATTGCGTTAAGCAGTATCAATAAAGCCGTCGTCTTCCCGTTCAACTTACCCGCGGTGGCTGAACTGGGTACCGCGTCAGGTTTTGATATGGAACTGCTGGACAACGGTAACCTGGGGCACGAAAAACTAACCCAGGCGCGAAACGAGCTGTTATCACTGGCAGCGCAATCACCGAATCAGGTCACCGGGGTACGCCCGAACGGCCTGGAAGATACGCCGATGTTCAAAGTGAACGTCAACGCTGCGAAAGCTGAAGCGATGGGCGTGGCGCTGTCTGATATCAACCAGACAATTTCCACCGCCTTCGGCAGCAGCTACGTGAACGACTTCCTCAACCAGGGGCGGGTGAAAAAAGTGTATGTCCAGGCAGGCACGCCGTTCCGTATGTTGCCGGATAACATCAACCAATGGTATGTACGCAACGCCTCTGGCACGATGGCACCGCTTTCTGCCTACTCGTCTACCGAATGGACCTATGGTTCACCGCGACTGGAACGCTACAACGGCATCCCGTCAATGGAGATTTTAGGTGAAGCGGCGGCCGGGAAAAGTACCGGTGACGCCATGAAATTTATGGCAGACCTGGTCGCTAAACTTCCGGCAGGCGTCGGCTACTCATGGACCGGACTATCGTATCAGGAAGCGTTATCCTCAAATCAGGCTCCTGCGCTGTATGCGATTTCACTGGTCGTGGTGTTCCTCGCCCTCGCCGCACTCTATGAGAGCTGGTCAATTCCGTTCTCGGTGATGTTGGTTGTTCCGTTAGGCGTCGTTGGCGCATTACTGGCCACCGATCTGCGCGGCTTAAGTAATGACGTCTACTTCCAGGTTGGTTTGCTGACCACCATCGGGCTTTCCGCCAAAAACGCCATCCTGATTGTCGAATTTGCCGTTGAGATGATGCAGAAAGAAGGGAAAACGCCGATAGAGGCAATCATCGAAGCGGCGCGGATGCGTTTACGCCCAATCCTGATGACCTCTCTGGCCTTTATTCTCGGCGTGCTGCCGCTGGTTATCAGTCATGGTGCCGGTTCTGGCGCGCAAAACGCGGTAGGTACCGGCGTGATGGGCGGGATGTTTGCCGCAACAGTGCTGGCAATTTACTTCGTTCCGGTCTTTTTCGTTGTAGTGGAACATCTCTTTGCCCGCTTTAAAAAAGCGGCTAGCGGTGAAAACCTGTACTTCCAGAGCCTCGAGCACCACCACCACCACCACTGACCCGGGTACCGAGCTCGAATTCACTGGCCGTCGTTTTACAACGTCGTGACTGGGAAAACCCTGGCGTTACCCAACTTAATCGCCTTGCAGCACATCCCCCTTTCGCCAGCTGGCGTAATAGCGAAGAGGCCCGCACCGATCGCCCTTCCCAACAGTTGCGCAGCCTGAATGGCGAATGGCGCCTGATGCGGTATTTTCTCCTTACGCATCTGTGCGGTATTTCACACCGCATATGGTGCACTCTCAGTACAATCTGCTCTGATGCCGCATAGTTAAGCCAGCCCCGACACCCGCCAACACCCGCTGACGCGCCCTGACGGGCTTGTCTGCTCCCGGCATCCGCTTACAGACAAGCTGTGACCGTCTCCGGGAGCTGCATGTGTCAGAGGTTTTCACCGTCATCACCGAAACGCGCGAGACGAAAGGGCCTCGTGATACGCCTATTTTTATAGGTTAATGTCATGATAATAATGGTTTCTTAGACGTCAGGTGGCACTTTTCGGGGAAATGTGCGCGGAACCCCTATTTGTTTATTTTTCTAAATACATTCAAATATGTATCCGCTCATGAGACAATAACCCTGATAAATGCTTCAATAATATTGAAAAAGGAAGAGTATGAGTATTCAACATTTCCGTGTCGCCCTTATTCCCTTTTTTGCGGCATTTTGCCTTCCTGTTTTTGCTCACCCAGAAACGCTGGTGAAAGTAAAAGATGCTGAAGATCAGTTGGGTGCACGAGTGGGTTACATCGAACTGGATCTCAACAGCGGTAAGATCCTTGAGAGTTTTCGCCCCGAAGAACGTTTTCCAATGATGAGCACTTTTAAAGTTCTGCTATGTGGCGCGGTATTATCCCGTATTGACGCCGGGCAAGAGCAACTCGGTCGCCGCATACACTATTCTCAGAATGACTTGGTTGAGTACTCACCAGTCACAGAAAAGCATCTTACGGATGGCATGACAGTAAGAGAATTATGCAGTGCTGCCATAACCATGAGTGATAACACTGCGGCCAACTTACTTCTGACAACGATCGGAGGACCGAAGGAGCTAACCGCTTTTTTGCACAACATGGGGGATCATGTAACTCGCCTTGATCGTTGGGAACCGGAGCTGAATGAAGCCATACCAAACGACGAGCGTGACACCACGATGCCTGTAGCAATGGCAACAACGTTGCGCAAACTATTAACTGGCGAACTACTTACTCTAGCTTCCCGGCAACAATTAATAGACTGGATGGAGGCGGATAAAGTTGCAGGACCACTTCTGCGCTCGGCCCTTCCGGCTGGCTGGTTTATTGCTGATAAATCTGGAGCCGGTGAGCGTGGGTCTCGCGGTATCATTGCAGCACTGGGGCCAGATGGTAAGCCCTCCCGTATCGTAGTTATCTACACGACGGGGAGTCAGGCAACTATGGATGAACGAAATAGACAGATCGCTGAGATAGGTGCCTCACTGATTAAGCATTGGTAACTGTCAGACCAAGTTTACTCATATATACTTTAGATTGATTTAAAACTTCATTTTTAATTTAAAAGGATCTAGGTGAAGATCCTTTTTGATAATCTCATGACCAAAATCCCTTAACGTGAGTTTTCGTTCCACTGAGCGTCAGACCCCGTAGAAAAGATCAAAGGATCTTCTTGAGATCCTTTTTTTCTGCGCGTAATCTGCTGCTTGCAAACAAAAAAACCACCGCTACCAGCGGTGGTTTGTTTGCCGGATCAAGAGCTACCAACTCTTTTTCCGAAGGTAACTGGCTTCAGCAGAGCGCAGATACCAAATACTGTTCTTCTAGTGTAGCCGTAGTTAGGCCACCACTTCAAG

pET15b-MdtF

TCGGGGCGCAGCCATGACCCAGTCACGTAGCGATAGCGGAGTGTATACTGGCTTAACTATGCGGCATCAGAGCAGATTGTACTGAGAGTGCACCATTGCGGTGTGAAATACCGCACAGATGCGTAAGGAGAAAATACCGCATCAGGCGCTCTTCCGCTTCCTCGCTCACTGACTCGCTGCGCTCGGTCGTTCGGCTGCGGCGAGCGGTATCAGCTCACTCAAAGGCGGTAATACGGTTATCCACAGAATCAGGGGATAACGCAGGAAAGAACATGTGAGCAAAAGGCCAGCAAAAGGCCAGGAACCGTAAAAAGGCCGCGTTGCTGGCGTTTTTCCATAGGCTCCGCCCCCCTGACGAGCATCACAAAAATCGACGCTCAAGTCAGAGGTGGCGAAACCCGACAGGACTATAAAGATACCAGGCGTTTCCCCCTGGAAGCTCCCTCGTGCGCTCTCCTGTTCCGACCCTGCCGCTTACCGGATACCTGTCCGCCTTTCTCCCTTCGGGAAGCGTGGCGCTTTCTCATAGCTCACGCTGTAGGTATCTCAGTTCGGTGTAGGTCGTTCGCTCCAAGCTGGGCTGTGTGCACGAACCCCCCGTTCAGCCCGACCGCTGCGCCTTATCCGGTAACTATCGTCTTGAGTCCAACCCGGTAAGACACGACTTATCGCCACTGGCAGCAGCCACTGGTAACAGGATTAGCAGAGCGAGGTATGTAGGCGGTGCTACAGAGTTCTTGAAGTGGTGGCCTAACTACGGCTACACTAGAAGGACAGTATTTGGTATCTGCGCTCTGCTGAAGCCAGTTACCTTCGGAAAAAGAGTTGGTAGCTCTTGATCCGGCAAACAAACCACCGCTGGTAGCGGTGGTTTTTTTGTTTGCAAGCAGCAGATTACGCGCAGAAAAAAAGGATCTCAAGAAGATCCTTTGATCTTTTCTACGGGGTCTGACGCTCAGTGGAACGAAAACTCACGTTAAGGGATTTTGGTCATGAGATTATCAAAAAGGATCTTCACCTAGATCCTTTTAAATTAAAAATGAAGTTTTAAATCAATCTAAAGTATATATGAGTAAACTTGGTCTGACAGTTACCAATGCTTAATCAGTGAGGCACCTATCTCAGCGATCTGTCTATTTCGTTCATCCATAGTTGCCTGACTCCCCGTCGTGTAGATAACTACGATACGGGAGGGCTTACCATCTGGCCCCAGTGCTGCAATGATACCGCGAGACCCACGCTCACCGGCTCCAGATTTATCAGCAATAAACCAGCCAGCCGGAAGGGCCGAGCGCAGAAGTGGTCCTGCAACTTTATCCGCCTCCATCCAGTCTATTAATTGTTGCCGGGAAGCTAGAGTAAGTAGTTCGCCAGTTAATAGTTTGCGCAACGTTGTTGCCATTGCTGCAGGCATCGTGGTGTCACGCTCGTCGTTTGGTATGGCTTCATTCAGCTCCGGTTCCCAACGATCAAGGCGAGTTACATGATCCCCCATGTTGTGCAAAAAAGCGGTTAGCTCCTTCGGTCCTCCGATCGTTGTCAGAAGTAAGTTGGCCGCAGTGTTATCACTCATGGTTATGGCAGCACTGCATAATTCTCTTACTGTCATGCCATCCGTAAGATGCTTTTCTGTGACTGGTGAGTACTCAACCAAGTCATTCTGAGAATAGTGTATGCGGCGACCGAGTTGCTCTTGCCCGGCGTCAACACGGGATAATACCGCGCCACATAGCAGAACTTTAAAAGTGCTCATCATTGGAAAACGTTCTTCGGGGCGAAAACTCTCAAGGATCTTACCGCTGTTGAGATCCAGTTCGATGTAACCCACTCGTGCACCCAACTGATCTTCAGCATCTTTTACTTTCACCAGCGTTTCTGGGTGAGCAAAAACAGGAAGGCAAAATGCCGCAAAAAAGGGAATAAGGGCGACACGGAAATGTTGAATACTCATACTCTTCCTTTTTCAATATTATTGAAGCATTTATCAGGGTTATTGTCTCATGAGCGGATACATATTTGAATGTATTTAGAAAAATAAACAAATAGGGGTTCCGCGCACATTTCCCCGAAAAGTGCCACCTGACGTCTAAGAAACCATTATTATCATGACATTAACCTATAAAAATAGGCGTATCACGAGGCCCTTTCGTCTTCAAGAATTCTCATGTTTGACAGCTTATCATCGATAAGCTTTAATGCGGTAGTTTATCACAGTTAAATTGCTAACGCAGTCAGGCACCGTGTATGAAATCTAACAATGCGCTCATCGTCATCCTCGGCACCGTCACCCTGGATGCTGTAGGCATAGGCTTGGTTATGCCGGTACTGCCGGGCCTCTTGCGGGATATCCGGATATAGTTCCTCCTTTCAGCAAAAAACCCCTCAAGACCCGTTTAGAGGCCCCAAGGGGTTATGCTAGTTATTGCTCAGCGGTGGCAGCAGCCAACTCAGCTTCCTTTCGGGCTTTGTTAGCAGCCGGATCTCAGTGGTGGTGGTGGTGGTGCTCGAGGCTCTGGAAGTACAGGTTTTCACCGCTAGCCGCTTTTTTAAAGCGGGCAAAGAGATGTTCCACTACAACGAAAAAGACCGGAACGAAGTAAATTGCCAGCACTGTTGCGGCAAACATCCCGCCCATCACGCCGGTACCTACCGCGTTTTGCGCGCCAGAACCGGCACCATGACTGATAACCAGCGGCAGCACGCCGAGAATAAAGGCCAGAGAGGTCATCAGGATTGGGCGTAAACGCATCCGCGCCGCTTCGATGATTGCCTCTATCGGCGTTTTCCCTTCTTTCTGCATCATCTCAACGGCAAATTCGACAATCAGGATGGCGTTTTTGGCGGAAAGCCCGATGGTGGTCAGCAAACCAACCTGGAAGTAGACGTCATTACTTAAGCCGCGCAGATCGGTGGCCAGTAATGCGCCAACGACGCCTAACGGAACAACCAACATCACCGAGAACGGAATTGACCAGCTCTCATAGAGTGCGGCGAGGGCGAGGAACACCACGACCAGTGAAATCGCATACAGCGCAGGAGCCTGATTTGAGGATAACGCTTCCTGATACGATAGTCCGGTCCATGAGTAGCCGACGCCTGCCGGAAGTTTAGCGACCAGGTCTGCCATAAATTTCATGGCGTCACCGGTACTTTTCCCGGCCGCCGCTTCACCTAAAATCTCCATTGACGGGATGCCGTTGTAGCGTTCCAGTCGCGGTGAACCATAGGTCCATTCGGTAGACGAGTAGGCAGAAAGCGGTGCCATCGTGCCAGAGGCGTTGCGTACATACCATTGGTTGATGTTATCCGGCAACATACGGAACGGCGTGCCTGCCTGGACATACACTTTTTTCACCCGCCCCTGGTTGAGGAAGTCGTTCACGTAGCTGCTGCCGAAGGCGGTGGAAATTGTCTGGTTGATATCAGACAGCGCCACGCCCATCGCTTCAGCTTTCGCAGCGTTGACGTTCACTTTGAACATCGGCGTATCTTCCAGGCCGTTCGGGCGTACCCCGGTGACCTGATTCGGTGATTGCGCTGCCAGTGATAACAGCTCGTTTCGCGCCTGGGTTAGTTTTTCGTGCCCCAGGTTACCGTTGTCCAGCAGTTCCATATCAAAACCTGACGCGGTACCCAGTTCAGCCACCGCGGGTAAGTTGAACGGGAAGACGACGGCTTTATTGATACTGCTTAACGCAATCATTGCCCGCTGAATGATCGCGGTAACCGAGTTTTCCTCACCGACACGTTCAGACCACGGCTTGAGACTGATAAACGCCAGGCCGTTGTTTTGCCCCTGACCGCTGAAGCCAAAGCCGCCAACGGTAAACACCGACTGGACATTATCTTTCTCTTTAGTCAGATAATAATCCGTCACCTGTTGCAGCACTTTCGTGGTGTTAACCATGGTGGCACCGGAAGGTAACTGCGCGGTGGTCATAAATACCCCCTGATCCTCTTCTGGTAAGAAAGAGGTCGGCGTGCGCAGGAACAGCACCGCCATCCCGGCGCAAATCAGCAGGTAGACCACCATGTAGCGACCGGTACAACGCAACAGCGAGCGGGTGCTATCGGTATAGTGTTGAGTTGATTTTTCAAACAGCGTGTTGAAGCGTGCGAACAGGGCGTTAGGTTTGTGACCGCCTTCCGGCGCGGCTTTCAGAATGGTGGCGCACAGGGCAGGGGTCAGGCTCATTGCCACAAATACTGAAAGCAGCATGGAGGAGATCAGCGTGATGGAGAACTGGCGGTAGATCTCCCCGGTTGCACCGCTCATAAAGGCCATCGGCATAAACACTGCGGAAAGAACAACGGCAATACCGACCAGCGCACGTTGGATCTGCCCCATCGATTTATGCGTCGCTTCCTTCGGCGGTAGCTTATCTTCCGCAATGACACGCTCGACGTTCTCCACCACCACGATGGCGTCATCCACCAGTAACCCTATCGCCAGCACCATCCCGAACATAGTCAACGTGTTGATGGTGAAACCGACCGCCGACAAGATCGCAAACGTCCCGAGAATAACCACCGGTACGGCAATCGTCGGGATGATTGTGGCACGGAAATTCTGCAAAAACAGATACATGACCAGGAAGACTAGGATGATAGCCTCAACCAGTGTTTTGAAAACTTCCTGAATAGAAATTTCGATAAACGGCGTGGTGTCGTAAGGATAAACCGTCTTCAGACTTGCCGGGAAATAGGCTGATAAGCGGTTCAGTTCCTCTTTGACTGCCCGCGAGGTATCCAGGGCGTTTGCTCCGGCAGCCAGTTTGATGGCGATCCCGGCAGCAGGTTTGCCGTTATAGCGTGCCACGGTGGAATAATCTTCCGCCCCAAGTTCGACGCGAGCGACATCACGCAGCAGCACTTGCGAACCATCTTGCTGAACTTTCAACAGGATTTTGCCAAATTCTTCCGGCGTTTGCAGACGCGTCTGCACAATGATCGAGGCGTTTAGCTGCTGGTCTGCCGCCTGTGGCATGCCACCCAGTTGACCACCGGAAATCTGGTTGTTTTGCACCTTAATCTGGGAAATAACATCGGAAGGTACCAGGTTATATTTATTGAGTTTTTGCGGGTCCAGCCAGATACGCATGGCATACTCGGAACCAAAGAGTTGTACGCTACCAACGCCCGCGGTACGGCTTAGCGGGTCTTTGATATTAGACGCTACATAGTCCGCGATATCGTACTGGTTGAGGCTGCCGTTATCAGAAATAAACGCCGCTACCATCAGGATATTACTGCTCGACTTATCGACGCTAATCCCCTGCTGCTGCACTGCTTCAGGTAATGAAGGCATAGCGAGTTGCAGTTTATTTTGCACTTGAACCTGTGCGATATCAGGAGATGTCCCAGTCTCGAAGGTCAGAGTGATAGAGGCATTGCCCGCCGCATCACTGGTTGAAGACATGTACATCAGGCCATCAAGCCCATTCATATTTTGCTCAATCACCTGAGTGACCGAGTCTTCTACCGTTTGCGCATCGGCACCTGGATAGGTAGCGCTGACGGTAATGGTCGGTGGCGCAATCTGCGGATACTGCGCAACCGGTAAGTTCATGATCGCCAGACCACCTGCAAGCATCATAATAATGGCAAGTACCCAGGCAAAAACCGGGCGATCAATAAAATAGTTAGCCATATGTATATCTCCTTCTTAAAGTTAAACAAAATTATTTCTAGAGGGGAATTGTTATCCGCTCACAATTCCCCTATAGTGAGTCGTATTAATTTCGCGGGATCGAGATCTCGATCCTCTACGCCGGACGCATCGTGGCCGGCATCACCGGCGCCACAGGTGCGGTTGCTGGCGCCTATATCGCCGACATCACCGATGGGGAAGATCGGGCTCGCCACTTCGGGCTCATGAGCGCTTGTTTCGGCGTGGGTATGGTGGCAGGCCCCGTGGCCGGGGGACTGTTGGGCGCCATCTCCTTGCATGCACCATTCCTTGCGGCGGCGGTGCTCAACGGCCTCAACCTACTACTGGGCTGCTTCCTAATGCAGGAGTCGCATAAGGGAGAGCGTCGAGATCCCGGACACCATCGAATGGCGCAAAACCTTTCGCGGTATGGCATGATAGCGCCCGGAAGAGAGTCAATTCAGGGTGGTGAATGTGAAACCAGTAACGTTATACGATGTCGCAGAGTATGCCGGTGTCTCTTATCAGACCGTTTCCCGCGTGGTGAACCAGGCCAGCCACGTTTCTGCGAAAACGCGGGAAAAAGTGGAAGCGGCGATGGCGGAGCTGAATTACATTCCCAACCGCGTGGCACAACAACTGGCGGGCAAACAGTCGTTGCTGATTGGCGTTGCCACCTCCAGTCTGGCCCTGCACGCGCCGTCGCAAATTGTCGCGGCGATTAAATCTCGCGCCGATCAACTGGGTGCCAGCGTGGTGGTGTCGATGGTAGAACGAAGCGGCGTCGAAGCCTGTAAAGCGGCGGTGCACAATCTTCTCGCGCAACGCGTCAGTGGGCTGATCATTAACTATCCGCTGGATGACCAGGATGCCATTGCTGTGGAAGCTGCCTGCACTAATGTTCCGGCGTTATTTCTTGATGTCTCTGACCAGACACCCATCAACAGTATTATTTTCTCCCATGAAGACGGTACGCGACTGGGCGTGGAGCATCTGGTCGCATTGGGTCACCAGCAAATCGCGCTGTTAGCGGGCCCATTAAGTTCTGTCTCGGCGCGTCTGCGTCTGGCTGGCTGGCATAAATATCTCACTCGCAATCAAATTCAGCCGATAGCGGAACGGGAAGGCGACTGGAGTGCCATGTCCGGTTTTCAACAAACCATGCAAATGCTGAATGAGGGCATCGTTCCCACTGCGATGCTGGTTGCCAACGATCAGATGGCGCTGGGCGCAATGCGCGCCATTACCGAGTCCGGGCTGCGCGTTGGTGCGGATATCTCGGTAGTGGGATACGACGATACCGAAGACAGCTCATGTTATATCCCGCCGTTAACCACCATCAAACAGGATTTTCGCCTGCTGGGGCAAACCAGCGTGGACCGCTTGCTGCAACTCTCTCAGGGCCAGGCGGTGAAGGGCAATCAGCTGTTGCCCGTCTCACTGGTGAAAAGAAAAACCACCCTGGCGCCCAATACGCAAACCGCCTCTCCCCGCGCGTTGGCCGATTCATTAATGCAGCTGGCACGACAGGTTTCCCGACTGGAAAGCGGGCAGTGAGCGCAACGCAATTAATGTAAGTTAGCTCACTCATTAGGCACCGGGATCTCGACCGATGCCCTTGAGAGCCTTCAACCCAGTCAGCTCCTTCCGGTGGGCGCGGGGCATGACTATCGTCGCCGCACTTATGACTGTCTTCTTTATCATGCAACTCGTAGGACAGGTGCCGGCAGCGCTCTGGGTCATTTTCGGCGAGGACCGCTTTCGCTGGAGCGCGACGATGATCGGCCTGTCGCTTGCGGTATTCGGAATCTTGCACGCCCTCGCTCAAGCCTTCGTCACTGGTCCCGCCACCAAACGTTTCGGCGAGAAGCAGGCCATTATCGCCGGCATGGCGGCCGACGCGCTGGGCTACGTCTTGCTGGCGTTCGCGACGCGAGGCTGGATGGCCTTCCCCATTATGATTCTTCTCGCTTCCGGCGGCATCGGGATGCCCGCGTTGCAGGCCATGCTGTCCAGGCAGGTAGATGACGACCATCAGGGACAGCTTCAAGGATCGCTCGCGGCTCTTACCAGCCTAACTTCGATCATTGGACCGCTGATCGTCACGGCGATTTATGCCGCCTCGGCGAGCACATGGAACGGGTTGGCATGGATTGTAGGCGCCGCCCTATACCTTGTCTGCCTCCCCGCGTTGCGTCGCGGTGCATGGAGCCGGGCCACCTCGACCTGAATGGAAGCCGGCGGCACCTCGCTAACGGATTCACCACTCCAAGAATTGGAGCCAATCAATTCTTGCGGAGAACTGTGAATGCGCAAACCAACCCTTGGCAGAACATATCCATCGCGTCCGCCATCTCCAGCAGCCGCACGCGGCGCATCTCGGGCAGCGTTGGGTCCTGGCCACGGGTGCGCATGATCGTGCTCCTGTCGTTGAGGACCCGGCTAGGCTGGCGGGGTTGCCTTACTGGTTAGCAGAATGAATCACCGATACGCGAGCGAACGTGAAGCGACTGCTGCTGCAAAACGTCTGCGACCTGAGCAACAACATGAATGGTCTTCGGTTTCCGTGTTTCGTAAAGTCTGGAAACGCGGAAGTCAGCGCCCTGCACCATTATGTTCCGGATCTGCATCGCAGGATGCTGCTGGCTACCCTGTGGAACACCTACATCTGTATTAACGAAGCGCTGGCATTGACCCTGAGTGATTTTTCTCTGGTCCCGCCGCATCCATACCGCCAGTTGTTTACCCTCACAACGTTCCAGTAACCGGGCATGTTCATCATCAGTAACCCGTATCGTGAGCATCCTCTCTCGTTTCATCGGTATCATTACCCCCATGAACAGAAATCCCCCTTACACGGAGGCATCAGTGACCAAACAGGAAAAAACCGCCCTTAACATGGCCCGCTTTATCAGAAGCCAGACATTAACGCTTCTGGAGAAACTCAACGAGCTGGACGCGGATGAACAGGCAGACATCTGTGAATCGCTTCACGACCACGCTGATGAGCTTTACCGCAGCTGCCTCGCGCGTTTCGGTGATGACGGTGAAAACCTCTGACACATGCAGCTCCCGGAGACGGTCACAGCTTGTCTGTAAGCGGATGCCGGGAGCAGACAAGCCCGTCAGGGCGCGTCAGCGGGTGTTGGCGGGTG

**Single Point Mutations**

Red, **bold**, and underlined text represents the introduction of the desired single point mutation.

Q37T

GGGGAAATNNCCCCGGGGGGGGGGTTTTTNTTAAAAACAANNTTTTGGAGGGGGNAAAGGAACCCGGGCGTTTCCGTTTTTCAGTTTACCCGCCCCTTTTCCGGGCGGGTTAAATTCCCCAAAGGGCTGCTGGGAAAGCGTNTCTACGCCCAGAATGCCCGCTTCCCCTTTAACCGCCNGGCATCGTTNTGAAGCCCAATTACGTTGCCGTCAGGATTACGACACCGCGCGCACCCATTGAATGAAGCAGAAGCCAATGTCACCGTCGCCAAAGCGGCTGTGAACAGGCGACGATCAATCTGCAATACGCGAATGTCACCTCGCCGATTACGGCGTCAGCGGGAAATCGTCGGTGACCGTCGGCGCACTCGTTACCGCTAATCAGGCAGATTCGCTGGTTACCGTACAACGTCTGGACCCGATTTATGTCGATCTCACGCAGTCGGTGCAAGATTTCTTACGCATGAAAGAAGAGGTCGCCAGTGGGCAAATCAAACAGGTTCAGGGCAGTACGCCAGTACAGCTCAATCTGGAAAATGGTAAACGCTACAGCCAGACCGGCACGCTGAAATTCTCCGACCCGACAGTGGATGAAACCACGGGCTCCGTGACGTTACGGGCGATTTTCCCCAACCCAAATGGTGACTTGCTGCCTGGCATGTACGTCACGGCATTAGTGGATGAAGGTAGCCGCCAGAATGTATTACTGGTGCCGCAGGAAGGCGTCACCCACAACGCCCAGGGTAAAGCAACGGCGCTCATTCTGGATAAAGACGATGTCGTGCAGCTACGCGAAATTGAAGCCAGCAAAGCCATCGGCGACCAGTGGGTCGTCACCTCTGGCTTGCAGGCTGGCGATCGGGTGATCGTTTCCGGTTTGCAACGCATTCGTCCGGGTATCAAAGCACGAGCAATTTCCTCCAGCCAGGAAAACGCCAGCACCGAATCGAAACAATAACGTTGCAGGCTTAAGGGGACTTTCATGGCTAACTATTTTATTGATCGCCCGGTTTTTGCCTGGGTACTTGCCATTATTATGATGCTTGCAGGTGGTCTGGCGATCATGAACTTACCGGTTGCGCAGTATCCG**ACG**ATTGCGCCACCGACCATTACCGTCAGCGCTACCTATCCAGGTGCCGATGCGCAAACGGTAGAAGACTCGGTCACTCAGGTGATTGAGCAAAATATGAATGGGCTTGATGGCCTGATGTACATGTCTTCAACCAGTGATGCGGCGGGCAATGCCTCTATCACTCTGACCTTCGAGACTGGGACATCTCCTGATATCGCACAGGTTCAAGTGCAAAATAAACTGCAACTCGCCT

P100A

ATTGCGCCCCGACCATTACCGTCAGCGCTACCTATCCAGGTGCCGATGCGCAAACGGTAGAAGACTCGGTCACTCAGGTGATTGAGCAAAATATGAATGGGCTTGATGGCCTGATGTACATGTCTTCAACCAGTGATGCGGCGGGCAATGCCTCTATCACTCTGACCTTCGAGACTGGGACATCT**GCG**GATATCGCACAGGTTCAAGTGCAAAATAAACTGCAACTCGCTATGCCTTCATTACCTGAAGCAGTGCAGCAGCAGGGGATTAGCGTCGATAAGTCGAGCAGTAATATCCTGATGGTAGCGGCGTTTATTTCTGATAACGGCAGCCTCAACCAGTACGATATCGCGGACTATGTAGCGTCTAATATCAAAGACCCGCTAAGCCGTACCGCGGGCGTTGGTAGCGTACAACTCTTTGGTTCCGAGTATGCCATGCGTATCTGGCTGGACCCGCAAAAACTCAATAAATATAACCTGGTACCTTCCGATGTTATTTCCCAGATTAAGGTGCAAAACAACCAGATTTCCGGTGGTCAACTGGGTGGCATGCCACAGGCGGCAGACCAGCAGCTAAACGCCTCGATCATTGTGCAGACGCGTCTGCAAACGCCGGAAGAATTTGGCAAAATCCTGTTGAAAGTTCAGCAAGATGGTTCGCAAGTGCTGCTGCGTGATGTCGCTCGCGTCGAACTTGGGGCGGAAGATTATTCCACCGTGGCACGCTATAACGGCAAACCTGCTGCCGGGATCGCCATCAAACTGGCTGCCGGAGCAAACGCCCTGGATACCTCGCGGGCAGTCAAAGAGGAACTGAACCGCTTATCAGCCTATTTCCCGGCAAGTCTGAAGACGGTTTATCCTTACGACACCACGCCGTTTATCGAAATTTCTATTCAGGAAGTTTTCAAAACACTGGTTGAGGCTATCATCCTAGTCTTCCTGGTCATGTATCTGTTTTTGCAGAATTTCCGTGCCACAATCATCCCGACGATTGCCGTACCGGTGGTTATTCTCGGGACGTTTGCGATCTTGTCGGCGGTCGGTTTCACCATCAACACGTTGACTATGTTCGGGATGGTGCTGGCGATAGGGTTACTGGTGGATGACCCATCCTGGTGGTGGAAAACGTCAAGCGTGTCATTGCGGAAGATAAGCTACCGCCAAGGAACGACCCTAAATCGATGGGGCAGATCCAACGGGCCTGGTCGGAATGCCGTTGTCTTTCCCNNNGGTTTATGCCCAGGGCCCTTTTNNACGGGGCAACCCGGGGAATNCCCCCCNTTTCCCCCCCCGCGANATCCCCCCGGGGGGGTT

Q37T/P100A

AAACCCCCCCCCCCCCGGGGNNNGGGTGTTTTCCTCCNNNNCCNNNNCTTGTTGTTATNNGNNGGGACCCCGGGANCCCCTTTTTAAGGGGGGGGGGGGGCCCCCCCCCGGGGGGGTTTTTTTTTTTNNAAAAAANNNTTTAGGGGGGGGAAAAAAAAACGGGGGGGGTTTTTNNNTTTTTTTTTTTTCCCCCCTTCNTCGGGGGGNAAATTCCCCCANNGGGTGGGGGGGAAAAGGCTTTTTTCCCCCCCAAAAACCCNNNCCCCCCTTTTACCCCCCGGGGNNNTGTGGGGAANNNCAAATTTTTTTTCCCCCTTGGGTTTTGGACCCCCGGCCCCCCCNTTNNNNNNAACCNNAACCCAATTTCCCCNNTCCCCAAAGGGGGTNTTAACCGGGGGCGATCAATTTGCAATACGGGAATTTCACCTTGCCGATTACGGGGTTCAGCGGGAAATTGTCGGTGACCGTTGGGGCCATCGTTACCGTTAATCAGGCAGATTCGCTGGTTACCGTACAACGTCTGGACCCGATTTATGTCGATCTCAGGCAGTCGGTGCAAGATTTCTTACGCATGAAAGAAGAGGTCGCCAGTGGGCAAATCAAACAGGTTCAGGGCAGTACGCCAGTACAGCTCAATCTGGAAAATGGTAAACGCTACAGCCAGACCGGCACGCTGAAATTCTCCGACCCGACAGTGGATGAAACCACGGGCTCCGTGACGTTACGGGCGATTTTCCCCAACCCAAATGGTGACTTGCTGCCTGGCATGTACGTCACGGCATTAGTGGATGAAGGTAGCCGCCAGAATGTATTACTGGTGCCGCAGGAAGGCGTCACCCACAACGCCCAGGGTAAAGCAACGGCGCTCATTCTGGATAAAGACGATGTCGTGCAGCTACGCGAAATTGAAGCCAGCAAAGCCATCGGCGACCAGTGGGTCGTCACCTCTGGCTTGCAGGCTGGCGATCGGGTGATCGTTTCCGGTTTGCAACGCATTCGTCCGGGTATCAAAGCACGAGCAATTTCCTCCAGCCAGGAAAACGCCAGCACCGAATCGAAACAATAACGTTGCAGGCTTAAGGGGACTTTCATGGCTAACTATTTTATTGATCGCCCGGTTTTTGCCTGGGTACTTGCCATTATTATGATGCTTGCAGGTGGTCTGGCGATCATGAACTTACCGGTTGCGCAGTATCCG**ACG**ATTGCGCCACCGACCATTACCGTCAGCGCTACCTATCCAGGTGCCGATGCGCAAACGGTAGAAGACTCGGTCACTCAGGTGATTGAGCAAAATATGAATGGGCTTGATGGCCTGATGTACATGTCTTCAACCAGTGATGCGGCGGGCAATGCCTCTATCACTCTGACCTTCGAGACTGGGACATCT**GCG**GATATCGCACAGGTTCAAGTGCAAAATAAACTGCAACTCGCTATGCCTTCATTACNNAAGCANNNNNNGGN

D408A

ACTCGCGGGCAGTCAAAGAGGAACTGAACCGCTTATCAGCCTATTTCCCGGCAAGTCTGAAGACGGTTTATCCTTACGACACCACGCCGTTTATCGAAATTTCTATTCAGGAAGTTTTCAAAACACTGGTTGAGGCTATCATCCTAGTCTTCCTGGTCATGTATCTGTTTTTGCAGAATTTCCGTGCCACAATCATCCCGACGATTGCCGTACCGGTGGTTATTCTCGGGACGTTTGCGATCTTGTCGGCGGTCGGTTTCACCATCAACACGTTGACTATGTTCGGGATGGTGCTGGCGATAGGGTTACTGGTGGAT**GCG**GCCATCGTGGTGGTGGAGAACGTCGAGCGTGTCATTGCGGAAGATAAGCTACCGCCGAAGGAAGCGACGCATAAATCGATGGGGCAGATCCAACGTGCGCTGGTCGGTATTGCCGTTGTTCTTTCCGCAGTGTTTATGCCGATGGCCTTTATGAGCGGTGCAACCGGGGAGATCTACCGCCAGTTCTCCATCACGCTGATCTCCTCCATGCTGCTTTCAGTATTTGTGGCAATGAGCCTGACCCCTGCCCTGTGCGCCACCATTCTGAAAGCCGCGCCGGAAGGCGGTCACAAACCTAACGCCCTGTTCGCACGCTTCAACACGCTGTTTGAAAAATCAACTCAACACTATACCGATAGCACCCGCTCGCTGTTGCGTTGTACCGGTCGCTACATGGTGGTCTACCTGCTGATTTGCGCCGGGATGGCGGTGCTGTTCCTGCGCACGCCGACCTCTTTCTTACCAGAAGAGGATCAGGGGGTATTTATGACCACCGCGCAGTTACCTTCCGGTGCCACCATGGTTAACACCACGAAAGTGCTGCAACAGGTGACGGATTATTATCTGACTAAAGAGAAAGATAATGTCCAGTCGGTGTTTACCGTTGGCGGCTTTGGCTTCAGCGGTCAGGGGCAAAACAACGGCCTGGCGTTTATCAGTCTCAAGCCGTGGTCTGAACGTGTCGGTGAGGAAAACTCGGTTACCGCGATCATTCAGCGGGCAATGATTGCGTTAAGCAGTATCAATAAAGCCGTCGTCTTCCCGTTCAACTTACCCGNGGTGGCTGAC

V610F

NNGGCAGTCAAAGAGGAACTGAACCGCTTATCAGCCTATTTCCCGGCAAGTCTGAAGACGGTTTATCCTTACGACACCACGCCGTTTATCGAAATTTCTATTCAGGAAGTTTTCAAAACACTGGTTGAGGCTATCATCCTAGTCTTCCTGGTCATGTATCTGTTTTTGCAGAATTTCCGTGCCACAATCATCCCGACGATTGCCGTACCGGTGGTTATTCTCGGGACGTTTGCGATCTTGTCGGCGGTCGGTTTCACCATCAACACGTTGACTATGTTCGGGATGGTGCTGGCGATAGGGTTACTGGTGGATGACGCCATCGTGGTGGTGGAGAACGTCGAGCGTGTCATTGCGGAAGATAAGCTACCGCCGAAGGAAGCGACGCATAAATCGATGGGGCAGATCCAACGTGCGCTGGTCGGTATTGCCGTTGTTCTTTCCGCAGTGTTTATGCCGATGGCCTTTATGAGCGGTGCAACCGGGGAGATCTACCGCCAGTTCTCCATCACGCTGATCTCCTCCATGCTGCTTTCAGTATTTGTGGCAATGAGCCTGACCCCTGCCCTGTGCGCCACCATTCTGAAAGCCGCGCCGGAAGGCGGTCACAAACCTAACGCCCTGTTCGCACGCTTCAACACGCTGTTTGAAAAATCAACTCAACACTATACCGATAGCACCCGCTCGCTGTTGCGTTGTACCGGTCGCTACATGGTGGTCTACCTGCTGATTTGCGCCGGGATGGCGGTGCTGTTCCTGCGCACGCCGACCTCTTTCTTACCAGAAGAGGATCAGGGGGTATTTATGACCACCGCGCAGTTACCTTCCGGTGCCACCATGGTTAACACCACGAAAGTGCTGCAACAGGTGACGGATTATTATCTGACTAAAGAGAAAGATAATGTCCAGTCGGTGTTTACC**TTT**GGCGGCTTTGGCTTCAGCGGTCAGGGGCAAAACAACGGCCTGGCGTTTATCAGTCTCAAGCCGTGGTCTGAACGTGTCGGTGAGGAAAACTCGGTTACCGCGATCATTCAGCGGGCAATGATTGCGTTAAGCAGTATCAATAAAGCCGTCGTCTTCCCGTTCAACTTACCCGCGGTGGCTGAACTGGGTACCGCGTCAGGTTTTGAAATGGAACTGCTGGACAACGGTAACCTGGGGCACAAAAAACTAACCCAGGCCCGAAACAAACTT
